# Supplementary material for: Qualitative study of practices and attitudes towards physical activity among prediabetic men and women in urban and rural Malawi
Source: BMJ Open. 2023 Jan 17;13(1):e058261. doi: 10.1136/bmjopen-2021-058261 (PMC9853122; doi:10.1136/bmjopen-2021-058261)
Supplement: Supplementary data [file bmjopen-2021-058261supp001.pdf]

Supplementary materials

Physical activity focus group topic guide

1. Tell me about when you are physically active/do exercise in your daily life. What sort of things do you do? Where? Why? (Prompt for work, travel and leisure activity)
2. How would you say your activity levels compare with your parents/grandparents? Why is this? (Prompt changes to work, leisure and travel – moving to different place)
3. Have your own activity levels changed over the years? Why is this?
4. What are the benefits to you of being physically active? (Prompt: fitness, strength, flexibility) how important are these to you/other people in your community? Why is this?
5. What do you understand by exercise?
6. Do you ever do exercise? What do you do and where? Who do you do it with? What motivates you to do exercise?
7. Tell me about any social/community occasions which involve physical activity/exercise?
8. What do you know about physical activity and health? ([Prompt: Benefits and risks of being physically active, including in relation to diabetes) How do you know this? (Prompt: where did this knowledge come from?)
9. How could you do more physical activity in your daily life? What might stop you doing this?

Demonstrate and lead an exercise session including resistance exercises. Ask the participants what they think of them as they do them.

10. Afterwards, ask: which of these exercises might you do again (if any)? Why have you chosen these? What would help you do these regularly? What would stop you doing them regularly?
11. Which of the exercises would you not like to do again? Why is this?

Table S1: Detailed description and example extracts from the nine broad themes reported

Theme	Description	Example extracts
Context		
1. Types of physical activity undertaken	Activities participants reported doing that made them physically active or inactive	*The physical work which I usually do is, I like going to the lake and launch the fish nets. When I come back home, I make sure that outside the house is clean, but if it's not clean I tell the children to sweep the ground. Sometimes when we are in rainy season, I wake up early in the morning going to the farm garden, I do weeding – a little bit in maize garden because I don't do for a long time. Usually my work is fishing, I got in the morning going to the lake and arrange the fishing nets and go into the lake.* (Male, 76, rural, hypertension reported)<br><br>*In the morning I take my bath then I prepare to go to work, and mostly I do my work while seated working on the computer, if I move around that means I'm just delivering file to my boss's office, when I knock off from work I use a car off I go home.* (Male, 50, urban, no health conditions reported)
2. Changes in physical activity over time	How physical activity has changed from generation to generation.	*in the past people were digging manually by using a hoe while nowadays we are using animal plough whenever we want to cultivate any kind of crop. The other thing is, they [*past generations*] were carrying things on their head while nowadays we carry everything in an oxcart or in a vehicle … they like building structures while nowadays we like modern structures by hiring other people to do for us while we are sitting down. …* (Male, 25, rural, no health conditions reported)
Influences		
3. Physical activity and health	How participants perceive that being physically active influences people's health	*If I stay idle, diabetes disease will affect me because there is nothing I am doing. Any kind of disease can affect you if you are just staying idle but if I am physically active, I might be free from diseases and be strong*. (Female, 60, rural, hypertension reported)
4. Motivations	Participants' reasons for undertaking different physical activities	*The reason we are physically active especially here on earth, it's because we want to eat. Without this, there won't be anything to eat*. (Female, 56, rural, diabetes reported)
5. Barriers to physical activity	Factors hindering involvement in physical activity	*It's what we said that sometimes its laziness because you can plan to do the exercise, but you may end up doing other things*. (Female, 60, rural, hypertension reported)<br><br>*[the] gym is too far from where I live, I live [in one place] and gym is in [another place], mostly I fail to go to gym, and when I*

**Theme**	**Description**	**Example extracts**
		go to gym am supposed to pay a certain fee which makes me just give up on gym, but if I had all the needed weights at home I could have been doing exercises at home. (Male, 38, urban, no health condition reported)
6. Technology and physical activity	How technological advancements affect physical activity	*Technology is pushing us to be…. the same with those who live in rural areas…. let's talk of phones, even maize mills are everywhere nowadays, they no longer use mortar and pestle*. (Female, 34, urban, no health condition reported)
7. Social norms	How other people's views/commonly held opinions and expectations influence physical activity	*… My neighbor's asked me why I draw water by myself when I have children, I tell them it's part of my exercises*. (Female, 54, urban, no health conditions reported)<br>*There are some other exercises that women cannot manage to do like stretching your legs you cannot manage that…* (Female, 58, urban, hypertension reported)
8. Social support	People and social institutions providing support for physical activities	*At our church we have our own team as [name of church] women we play against the youths for practices, but when we have a match, we play against another [name of church] church*. (Female, 59, urban, hypertension reported)
Solutions		
9. Ways of improving physical activity	Suggested ways of improving physical activity	*I think a person should join more sports groups, working can be one of them and cycling can be one of them; you can also join groups which can motivate you to be moving or running and make you sweat.* (Male, 25, rural, no health conditions reported)

Table S2 Types of physical activity described by participants within the household, occupational, transport and recreational domains of the ecological model

Household*	Who				Occupational*	Who				Transport	Who				Recreational	Who			
	wr	wu	mr	mu		wr	wu	mr	mu		wr	wu	mr	mu		wr	wu	mr	mu
Chores (sweeping, mopping)	x	x	-	x	Farming (planting, weeding, harvesting)	x	-	x	-	Walking	x	x	x	x	Dance	x	x	x	-
Food shopping	x	x	x	-	Looking after livestock	-	-	x	-	Cycling	-	-	-	x	Team sports	x	x	x	x
Collecting farm produce for meals	x	-	-	-	Fishing on lake	-	-	x	-	Public transport	x	-	x	x	Playing with children	x	x	x	x
Cooking	x	x	-	x	Running shops/ market stalls	-	x	-	x	(Bicycle) taxi	-	-	x	x	Jogging	-	x	x	x
Food processing (pounding maize)	x	-	-	-	Service industry	-	-	-	x	Personal car	-	x	x	x	Cycling	-	-	x	x
Washing clothes	x	x	-	-	Office job	-	-	-	x	Transport goods (by car/ox cart)	-	x	x	-	Gym/exercise classes	-	x	-	x
Fetching water	x	x	-	-											Doing exercises at home	x	x	-	x
Cutting wood	-	-	x	-															
Carrying wood	x	-	-	-															
Gardening	-	x	x	x															
Moulding bricks	-	-	x	-															
Digging pit latrines	-	-	x	-															
Car washing, maintenance	-	-	-	x															
Feeding livestock	-	x	-	-															

*In the rural setting there appeared to be some overlap between household and occupational physical activities. wr = rural women, wu = urban women, mr = rural men, mu = urban men

Table S3: Broad themes and sub-themes summarising influences and their impact on participants' physical activity, mapped to ecological model levels and domains

Theme	Sub-theme	Who				Ecological Model		Impact (+ve/-ve)
		wr	wu	mr	mu	Level	Domain	
3) Health	Recognition of health/wellbeing benefits	x	x	x	x	Individual	General	+ve
3) Health	Weight loss	x	x	x	x	Individual	General	+ve
3) Health	Recognition of physical strength benefits	x	x	x	x	Individual	General	+ve
4) Motivations	Family responsibilities	x	-	-	-	Interpersonal	Household	+ve
4) Motivations	Work responsibilities	x	-	x	-	Interpersonal	Occupational	+ve
5) Barriers	Work responsibilities	-	x	-	x	Interpersonal	Recreational	-ve
5) Barriers	Family responsibilities	x	x	x	x	Interpersonal	Recreational	-ve
5) Barriers	Lack of time	-	x	-	x	Individual	Recreational	-ve
5) Barriers	Sense of urgency	-	x	-	x	Individual	General	-ve
5) Barriers	Age	x	x	x	x	Individual	General	-ve
5) Barriers	Ill health	x	x	x	x	Individual	General	-ve
5) Barriers	Fear of injury	x	x	x	x	Individual	General	-ve
5) Barriers	Poor diet	x	x	x	x	Individual	General	-ve
5) Barriers	Lack of exercise knowledge	x	-	-	-	Individual	Recreational	-ve
5) Barriers	Laziness	x	x	x	x	Individual	General	-ve
6) Technology	Maize mills	x	x	-	-	Environment	Household	-ve
6) Technology	Farming tools/machinery	-	-	x	-	Environment	Occupational	-ve
7) Social norms	Pressure to make money	-	-	-	x	Interpersonal	Occupational	-ve
7) Social norms	Wealth	x	x	x	x	Interpersonal	Household/Transport	-ve
7) Social norms	Availability of motorised transport	x	x	x	x	Environment	Transport	-ve
7) Social norms	People making fun of you (e.g., cycling)	-	-	-	x	Interpersonal	Transport	-ve
7) Social norms	Gender (e.g., at gym)	-	x	-	-	Interpersonal	Recreational	-ve
7) Social norms	Expectations that children will help	x	x	x	-	Interpersonal	Household	-ve
7) Social norms	Children making fun of you	x	x	-	-	Interpersonal	Recreational	-ve
8) Social support	Playing with children	x	x	x	x	Interpersonal	Recreational	+ve
8) Social support	Team sports/exercising with others	x	x	x	x	Interpersonal	Recreational	+ve

wr = rural women, wu = urban women, mr = rural men, mu = urban men
