# Supplementary material for: Qualitative study of practices and attitudes towards physical activity among prediabetic men and women in urban and rural Malawi
Source: BMJ Open. 2023 Jan 17;13(1):e058261. doi: 10.1136/bmjopen-2021-058261 (PMC9853122; doi:10.1136/bmjopen-2021-058261)
Supplement: Author's manuscript [file bmjopen-2021-058261.draft_revisions.pdf]

BMJ Open is committed to open peer review. As part of this commitment we make the peer review history of every article we publish publicly available.

When an article is published we post the peer reviewers' comments and the authors' responses online. We also post the versions of the paper that were used during peer review. These are the versions that the peer review comments apply to.

The versions of the paper that follow are the versions that were submitted during the peer review process. They are not the versions of record or the final published versions. They should not be cited or distributed as the published version of this manuscript.

BMJ Open is an open access journal and the full, final, typeset and author-corrected version of record of the manuscript is available on our site with no access controls, subscription charges or pay-per-view fees (http://bmjopen.bmj.com).

If you have any questions on BMJ Open's open peer review process please email info.bmjopen@bmj.com

BMJ Open

# A qualitative study of practices and attitudes towards physical activity among men and women in urban and rural Malawi

| | |
|---|---|
| Journal: | *BMJ Open* |
| Manuscript ID | bmjopen-2021-058261 |
| Article Type: | Original research |
| Date Submitted by the Author: | 14-Oct-2021 |
| Complete List of Authors: | Banda, Jethro; Malawi Epidemiology and Intervention Research Unit<br>Bunn, Christopher; University of Glasgow, School of Social and Political Sciences, Institute of Health and Wellbeing; Malawi Epidemiology and Intervention Research Unit<br>Crampin, Amelia; London School of Hygiene and Tropical Medicine; Malawi Epidemiology and Intervention Research Unit<br>Gill, Jason; University of Glasgow, Institute of Cardiovascular and Medical Sciences<br>Gray, Cindy; University of Glasgow, School of Social and Political Sciences, Institute of Health and Wellbeing |
| Keywords: | General diabetes < DIABETES & ENDOCRINOLOGY, PUBLIC HEALTH, QUALITATIVE RESEARCH |
| | |

SCHOLARONE™
Manuscripts

*I, the Submitting Author has the right to grant and does grant on behalf of all authors of the Work (as defined in the below author licence), an exclusive licence and/or a non-exclusive licence for contributions from authors who are: i) UK Crown employees; ii) where BMJ has agreed a CC-BY licence shall apply, and/or iii) in accordance with the terms applicable for US Federal Government officers or employees acting as part of their official duties; on a worldwide, perpetual, irrevocable, royalty-free basis to BMJ Publishing Group Ltd ("BMJ") its licensees and where the relevant Journal is co-owned by BMJ to the co-owners of the Journal, to publish the Work in this journal and any other BMJ products and to exploit all rights, as set out in our licence.*

*The Submitting Author accepts and understands that any supply made under these terms is made by BMJ to the Submitting Author unless you are acting as an employee on behalf of your employer or a postgraduate student of an affiliated institution which is paying any applicable article publishing charge ("APC") for Open Access articles. Where the Submitting Author wishes to make the Work available on an Open Access basis (and intends to pay the relevant APC), the terms of reuse of such Open Access shall be governed by a Creative Commons licence – details of these licences and which Creative Commons licence will apply to this Work are set out in our licence referred to above.*

*Other than as permitted in any relevant BMJ Author's Self Archiving Policies, I confirm this Work has not been accepted for publication elsewhere, is not being considered for publication elsewhere and does not duplicate material already published. I confirm all authors consent to publication of this Work and authorise the granting of this licence.*

For peer review only

# A qualitative study of practices and attitudes towards physical activity among men and women in urban and rural Malawi

Mr Jethro Banda, Malawi Epidemiology and Intervention Research Unit, Lilongwe, Malawi. Jethro.Banda@meiru.mw

Dr Christopher Bunn PhD, School of Social and Political Sciences, Institute of Health and Wellbeing, University of Glasgow, UK and Malawi Epidemiology and Intervention Research Unit, Lilongwe, Malawi. Christopher.Bunn@glasgow.ac.uk

Professor Amelia Crampin MBChB DTM&H MSc FFPHM, Malawi Epidemiology and Intervention Research Unit, Lilongwe, Malawi, Institute of Health and Wellbeing, University of Glasgow, UK and London School of Hygiene and Tropical Medicine, London, UK. Mia.Crampin@lshtm.ac.uk

Professor Jason MR Gill PhD, Institute of Cardiovascular and Medical Sciences, University of Glasgow, UK. Jason.Gill@glasgow.ac.uk

Professor Cindy M Gray* PhD, School of Social and Political Sciences, Institute of Health and Wellbeing, University of Glasgow, UK. cindy.gray@glasgow.ac.uk

*Corresponding author

For peer review only

## ABSTRACT

**Introduction** Malawi is experiencing a decline in physical activity levels, particularly in urban areas. As physical inactivity is associated with increased risk of non-communicable diseases, such as type 2 diabetes and hypertension, interventions are needed to promote physical activity. This study aimed to generate the in-depth understanding of physical activity practices and attitudes required to inform physical activity interventions in Malawi.

**Methods** Focus groups (N=4) were conducted with men and women in urban (men N=6, women N=9) and rural (men N=8, women N=9) Malawi, who had been identified as having pre-diabetes 3-5 years previously. Thematic analysis identified 10 themes relating to physical activity practices and attitudes, and the ecological model was used to classify the types of physical activity that people engaged with and identify opportunities for intervention.

**Results** Participants reported undertaking different types of physical activity across all ecological model domains (household, occupational, transport, recreational). Rural participants reported more vigorous physical activities than urban participants, and women reported more household activities than men. Many participants recognized a need to promote physical activity in Malawi, and the health benefits of doing so, including the importance of physical activity in helping them stay strong to maintain physical functioning. Barriers to being physically active included competing priorities (especially for urban men), societal expectations around wealth, use of motorized transport, lack of accessible facilities for women, aging and ill health.

**Conclusions** Physical activity is declining in Malawi as working and transport practices change in response to economic development, making the promotion of


For peer review only

alternative forms of physical activity a public health priority. Interventions promoting the personal benefits/value of physical activity for all ages, and routine and group-based exercising, as well as investment in accessible recreational facilities (including for women) and active travel infrastructure should be urgently considered to improve physical activity levels in Malawi.

**Keywords** Physical activity, facilitators, barriers, interventions, ecological model, qualitative, sub-Saharan Africa, Malawi.

**Strengths and limitations of this study**

- This is the first in-depth exploration of physical activity among community-dwelling adults in Malawi.
- Conducting separate focus groups with men and women in urban and rural settings allowed systematic comparison of urban/rural and gender differences in physical activity.
- Participants had previously been told they were at high risk of developing diabetes and may been more informed about the importance of physical activity for their health than the general adult population of Malawi.



For peer review only - http://bmjopen.bmj.com/site/about/guidelines.xhtml

**What is already known?**

- Increasing levels of physical inactivity among men and women in Malawi are becoming a public health concern as morbidity and mortality from non-communicable diseases increase.

**What are the new findings?**

- Men and women in both urban and rural Malawi acknowledge the decline in physical activity and possible impact on their health.
- Societal pressures, time constraints (particularly for urban men), aging and ill-health emerged as important barriers to being physically active.

**What do the new findings imply?**

- Increasing physical activity in Malawi requires intervening at multiple levels.
- Individual and interpersonal-level initiatives should focus on the personal benefits and value of routine and recreational group-based physical activity
- Environmental/policy level investment is needed to increase availability of fully accessible physical activity facilities and active travel infrastructure such as pavements.

For peer review only

## INTRODUCTION

Lack of physical activity is thought to be the fourth leading risk factor for premature mortality globally.[1] The World Health Organization (WHO) recommends that adults undertake 150 minutes of moderate-intensity or 75 minutes of vigorous-intensity physical activity a week for optimal health.[2] However, a quarter of adults fail to meet these guidelines worldwide.[2] In sub-Saharan Africa, physical activity levels have historically been high, but increased urbanization and adoption of Westernized lifestyles mean physical inactivity is increasing.[3-6] In Malawi, the prevalence of physical inactivity rose from 10% in 2011[7] to 14% in 2016,[8] with some studies reporting lower physical activity levels in urban settings;[9] for example, a WHO population survey reported 24% of people in urban areas were inactive compared with 9% in rural areas.[10] Gender differences are less clear: while population surveys report that men in Malawi are generally more physically active than women;[10 11] however, some studies in specific urban and rural settings show that women are more active than men.[12 13]

The prevalence of non-communicable diseases (NCD), such as diabetes, cardiovascular disease, chronic respiratory disease and cancer, is on the rise in Sub-Saharan Africa.[14 15] Deaths from NCD in low and middle income countries are forecast to increase from 31.5 million in 2016 to 41.9 million in 2030 and to 56.6 million by 2045.[16] Recent estimates suggest that 32% of deaths in Malawi are associated with NCD.[1] Although being physically active helps both prevention and management of NCD,[17-19] currently Malawi has no physical activity public health campaigns.[1]

Promotion of behaviour change for physical activity requires detailed understanding of current local practices and attitudes, as well as the different contexts in which physical



For peer review only - http://bmjopen.bmj.com/site/about/guidelines.xhtml

activity is undertaken. However, only a handful of qualitative studies exploring practices and attitudes towards physical activity in sub-Saharan Africa have been conducted. Interviews and focus group discussions with urban women in Ghana revealed a desire for more social (including group-based) physical activity, and that lack of time and concerns about injury (e.g., doing age-appropriate exercises) were other barriers to being physically active.[20] Three studies with women in urban South Africa[21-23] showed that although participants were aware of the physical and mental health benefits of physical activity (including weight loss); lack of time, money and information, perceptions of local crime, and negative influences from other people, including abuse from men for wearing "exercise" clothing, were barriers to their being more physically active. Focus groups with men and women with suspected type 2 diabetes, suspected pre-diabetes or who were obese in urban and rural Uganda suggested that people were willing to become more active to improve their health, and some described doing this by embedding physical activity within their normal routines.[24] This included washing, cooking, walking and cycling in urban areas, and more vigorous activities such as gardening, bush clearing, fetching water, splitting firewood and pounding food in rural areas. However, any gender differences were not explored.

Two qualitative studies on physical activity have been conducted in Malawi in men and women with diabetes. In one study, interviews and focus groups with diabetes patients attending health education classes at a rural private hospital revealed that people were aware of the importance of both routine and leisure (recreational) physical activity for their health; however, no gender comparisons were reported.[25] In a separate study, focus group discussions with patients in urban and semi-urban areas reported both positive and negative social influences. Specifically, while ridicule from other people



For peer review only - http://bmjopen.bmj.com/site/about/guidelines.xhtml

hindered their participation in some types of physical activity, encouragement from health workers and family members (including taking part in household activities) helped them remain active.[26]

Despite declining physical activity levels[6 15] and the implication of this for NCD prevention and management,[17-19] there is currently very little evidence about the practices and attitudes of adults in Malawi towards physical activity. As physical activity may differ according to gender and place of residence,[2 27-30] the aim of this qualitative study was to compare and contrast accounts of physical activity in the daily lives of men and women who had previously been identified as having pre-diabetes (a population in which physical activity interventions to reduce risk are likely to be targeted) in urban and rural settings in Malawi, to understand the factors influencing the amount and types of physical activity undertaken.

## METHODS

### Setting

Data collection took place in Lilongwe (urban) and Karonga (rural) in March 2019. Participants were identified from a sample of adults previously identified as having pre-diabetes within a larger MRC-funded study (MR/R019428/1; NHSRC protocol 18/01/1951) investigating links between nutritional intake and physical, metabolic and inflammatory phenotypes in pre-diabetic (n=50) and normoglycaemic (n=50) adult men and women. This study was itself nested in Malawi Epidemiology and Intervention Unit population studies, where fasting blood glucose was measured in NCD surveys conducted in 2013-2016. Anyone with elevated blood glucose levels was informed of their status immediately after the survey.

For peer review only

## Participants

Four focus groups were held with men and women separately in the urban and rural locations. They included a total of 32 participants (urban N=15, 6 men, 9 women; rural N=17, 8 men, 9 women). At the time of the current study, some participants reported progression from their pre-diabetic status at the last NCD survey to diabetes and/or hypertension.

## Participant and public involvement

At the start of the MRC-funded study, stakeholder engagement meetings with community leaders, community members, healthcare professionals, policy makers and patient group were held in both rural and urban locations to raise awareness of the research and gather feedback on the design and implementation of data collection procedures, including the focus group discussions reported here.

## Data collection and management

The focus groups lasted between 90 and 120 minutes and were audio recorded with participant consent. A topic guide (see Supplementary materials, Table 1) was used to focus discussion around participants' personal accounts of physical activity in their daily lives, their knowledge of physical activity and health (including the benefits of being physically active), perceived barriers and facilitators to being physically active (both personal and among others in their community) and their perceptions of changes in physical activity over time. The topic guide was developed in English by CB, CMG and Hazel Namadingo (HN), all experienced qualitative researchers, then translated into Chichewa for urban focus groups and into Chitumbuka for rural focus groups. The urban focus groups were conducted by HN; the rural focus groups were conducted by



For peer review only - http://bmjopen.bmj.com/site/about/guidelines.xhtml

Cecelia Nyirenda, an experienced field worker. The facilitators were fluent in the local languages and familiar with the issues under discussion.

The audio recordings, together with field notes taken during the focus groups, were transcribed directly into English by experienced field workers. HN and JB then quality assured the transcripts while listening to the audio recordings, and made changes where necessary.

**Data analysis**

The quality-assured transcripts were analyzed using a reflective thematic approach.[31 32] JB, CMG and CB read through the transcripts line by line to identify preliminary codes relating to participants' accounts, knowledge, barriers and facilitators of physical activity, and any emerging areas of interest (such as changes in physical activity over time in Malawi). The three authors then met to agree a coding framework consisting of 10 broad themes: 1) Types of physical activity undertaken; 2) Changes in physical activity over time; 3) Physical activity and health; 4) Motivations; 5) Barriers and facilitators; 6) Social norms; 7) Social support; 8) Technology and physical activity; 9) Sources of information; and 10) Ways of improving physical activity. A detailed description of each theme is provided in Supplementary materials. JB applied the coding framework to all transcripts using NVivo11 software to organize the data. The coded transcripts were reviewed by CMG, and any disagreements resolved through discussion. JB and CMG developed a detailed descriptive summary of each broad theme, which included careful comparison of differences in accounts between men and women and between urban and rural focus groups.

The ecological model of physical activity[33 34] was then applied as a framework to characterize the different types of physical activity participants engaged in. The



For peer review only - http://bmjopen.bmj.com/site/about/guidelines.xhtml

ecological model constructs physical activity as influenced by interacting factors operating at different levels: individual, interpersonal, environmental (social, built and natural) and policy.[34] It further organizes physical activity around four active living domains: household, occupational, transport and recreational[33] which were used to report the data. The findings presented below are illustrated by extracts with anonymised participant identifiers that include gender, age, setting (urban/rural) and any self-reported health conditions at the time of the current study.

## RESULTS

As Table 1 shows, 50% (16/32) of the focus group participants were aged 50 and above. On average, urban participants were slightly younger (43.1 years, SD=12.3) than their rural counterparts (51.6 years, SD=16.8), and length of stay was much shorter in the urban (17.9 years, SD=7.2) than the rural (36.2 years, SD=17.9) setting. Overall, 53% (17/32) had not been educated beyond primary school, but urban participants were better educated than rural participants (73%, 11/15 of urban participants had secondary school or higher education compared with only 24%, 4/17 of rural participants). Most urban participants (80%, 12/15) were either self-employed or salaried, whereas almost all rural participants (94%, 16/17) were involved in subsistence activities. Over half (66%, 21/32) were married, 25% (8/32, all women) reported being divorced/widowed and 9% (3/32, all men) had never married. The participants had all been identified as having pre-diabetes during previous demographic surveillance study visits between 2012 and 2017; some (9%, 3/32: one urban woman, one rural woman and one rural man) reported progression to diabetes, and 34.4% (11/32: two of whom also reported diabetes) reported a subsequent hypertension diagnosis.



For peer review only - http://bmjopen.bmj.com/site/about/guidelines.xhtml

**Table 1 Focus group participant characteristics**

| | All (N=32) (%) | Urban (N=15) (46.9%) | | | Rural (N=17) (53.1%) | | |
|---|---|---|---|---|---|---|---|
| | | Total | Female | Male | Total | Female | Male |
| **Sex** | | | | | | | |
| Male | 14(43.8) | 6(40.0) | - | 6(100) | 8(47.1) | - | 8(100) |
| Female | 18(56.3) | 9(60.0) | 9(100) | - | 9(52.9) | 9(100) | - |
| **Age** | | | | | | | |
| Age (years)[a] | 47.6(15.3) | 43.1(12.3) | 46.7(11.9) | 37.7(11.7) | 51.6(16.8) | 54.4(10.9) | 48.5(22.1) |
| ≤35 | 8(25.0) | 5(33.3) | 3(33.3) | 2(33.3) | 3(17.6) | 0(0.0) | 3(37.5) |
| 36-49 | 8(25.0) | 4(26.7) | 2(22.2) | 2(33.3) | 4(23.5) | 3(33.3) | 1(12.5) |
| ≥50 | 16(50.0) | 6(40.0) | 4(44.4) | 2(33.3) | 10(58.8) | 6(66.7) | 4(50.0) |
| **Length of stay** | | | | | | | |
| Length of stay (years)[a] | 27.7(16.6) | 17.9(7.2) | 20.1(8.4) | 14.7(3.0) | 36.2(17.9) | 29.8(12.6) | 43.5(21.1) |
| 6-10 years | 3(9.4) | 2(13.3) | 2(22.2) | 0(0.0) | 1(5.9) | 1(11.1) | 0(0.0) |
| 11-15 years | 5(15.6) | 5(33.3) | 1(11.1) | 4(66.7) | 0(0.0) | 0(0.0) | 0(0.0) |
| 16-20 years | 5(15.6) | 3(20.0) | 1(11.1) | 2(33.3) | 2(11.8) | 2(22.2) | 0(0.0) |
| ≥ 20 years | 19(59.4) | 5(33.3) | 5(55.6) | 0(0.0) | 14(82.3) | 8(88.9) | 6(75.0) |
| **Highest education** | | | | | | | |
| Primary school | 17(53.1) | 4(26.7) | 4(44.4) | 0(0.0) | 13(76.5) | 8(88.9) | 5(62.5) |
| Secondary and higher | 15(46.9) | 11(73.3) | 5(55.6) | 6(100.0) | 4(23.5) | 1(11.1) | 3(37.5) |
| **Employment** | | | | | | | |
| Not working | 2(6.3) | 2(13.3) | 2(22.2) | 0(0.0) | 0(0.0) | 0(0.0) | 0(0.0) |
| Subsistence | 17(53.1) | 1(6.7) | 1(11.1) | 0(0.0) | 16(94.1) | 8(88.9) | 8(100.0) |
| Self-employed | 8(25.0) | 8(53.3) | 5(55.6) | 3(50.0) | 0(0.0) | 0(0.0) | 0(0.0) |
| Salaried | 5(15.6) | 4(26.7) | 1(11.1) | 3(50.0) | 1(5.9) | 1(11.1) | 0(0.0) |
| **Marital Status** | | | | | | | |
| Never married | 3(9.4) | 1(6.7) | 0(0.0) | 1(16.7) | 2(11.8) | 0(0.0) | 2(25.0) |
| Married | 21(65.6) | 11(73.3) | 6(66.7) | 5(83.3) | 10(58.8) | 4(44.4) | 6(75.0) |
| Widowed/ divorced | 8(25.0) | 3(20.0) | 3(33.3) | 0(0.0) | 5(29.4) | 5(55.6) | 0(0.0) |
| **Reported health condition** | | | | | | | |
| Pre-diabetic only | 20(62.5) | 11(73.3) | 5(55.6) | 6(100.0) | 9(52.9) | 5(55.6) | 4(50.0) |
| Diabetic only | 1(3.1) | 0(0.0) | 0(0.0) | 0(0.0) | 1(5.9) | 1(11.1) | 0(0.0) |
| Hypertensive only | 9(28.1) | 3(20.0) | 3(33.3) | 0(0.0) | 6(35.3) | 3(33.3) | 3(37.5) |
| Diabetic and hypertensive | 2(6.3) | 1(6.7) | 1(11.1) | 0(0.0) | 1(5.9) | 0(0.0) | 1(12.5) |

Data are presented as n (%), or [a] mean (SD)



For peer review only - http://bmjopen.bmj.com/site/about/guidelines.xhtml

The following sections present participants' accounts of physical activity in their daily lives. In particular, they focus on what people do when they are physically active, the decline in physical activity over time, and factors (including social attitudes) influencing physical activity.

**What do adults in Malawi do when they are physically active?**

Our analysis of the types of physical activity undertaken showed that Malawian adults were active across all four domains of the ecological model.[33] Men and women in both urban and rural settings reported involvement in a range of household, occupational, transport and recreational activities. These are now described in turn.

*Household physical activity*

Overall, women reported doing more household physical activity than men. In both urban and rural settings, women talked about taking the lead in housework:

> *As a woman you are supposed to do physical activities which are household chores, we sweep around the surrounding, we prepare breakfast, we mop the house, then we go to the market to buy relish and some other things when you come back, time is already up for lunch, you start preparing lunch…* (Female, 59, urban, hypertension reported)

Rural women gave similar accounts of their household activities, but also described routine pounding of rice and cassava for flour, which is a vigorous physical activity. Men also reported some household-related physical activities, with those reported by rural men tending to be highly active:



For peer review only - http://bmjopen.bmj.com/site/about/guidelines.xhtml

*As for me I am physically active in the morning such as, cutting wood, weeding [gardening] at home […] In the afternoon I rest and thereafter around 4 pm I start again clearing outside the house.... During summer season I cut firewood…* (Male, 74, rural, hypertension reported)

By comparison, urban men described much lower and less strenuous forms of household activities, such as car washing/maintenance and cooking. Our analysis therefore supports the characterisation of rural Malawians as more active than their urban counterparts and suggests urban males are the least active group.

*Occupational physical activity*

For rural participants, most work appeared to involve effortful physical activity. Both women and men reported daily involvement in farming activities, including planting, weeding and harvesting their crops. One woman said:

*A person has a lot of work to do, like in this rainy season we do farming. Instead of sleeping you think of going to the garden to do farming activities […]. In the afternoon if you left some ridges which you didn't plant the seeds, you go back and plant the stems.* (Female, 53, rural, no health condition reported)

More than half of rural men described fishing on Lake Malawi, both to provide food for their families and to sell to make money. Like farming, fishing involved vigorous physical activity, including launching and rowing their boat and landing the catch. In contrast, urban men talked about running shops and market stalls, working in the service industry and having office jobs, which, as this man describes, were often largely sedentary:



For peer review only - http://bmjopen.bmj.com/site/about/guidelines.xhtml

*Mostly I do my work while seated working on the computer, if I move around that means I'm just delivering files to my boss' office, when I knock off from work I use a car…* (Male, 50, urban, no health condition reported)

Many urban women participants did not work outside the home (although they reported becoming involved in farming activities when they visited their villages). The exceptions were two women who had their own businesses. One of these described how running her market stall involved heavy physical work:

*…I sell timber at [local] market, I cut the timber myself I bought an electric saw blade, I have a wheelbarrow I use it to carry sand when the rains fall I push it myself as part of exercise.* (Female, 49, urban, no health condition reported)

In general, therefore, people in rural Malawi appear to be involved in more intense occupational physical activity compared to those in urban areas.

*Transport-related physical activity*

Most rural participants reported that walking was still their main mode of getting from place to place. Many described walking to work (their farm or to go fishing), to the market and to visit friends or family. Urban participants also reported walking as part of their daily routines, but to a lesser extent than their rural counterparts. Many preferred to use public transport, taxis (including bicycle taxis) or their own cars instead of walking:

*I cannot go to some places by foot while I have a car. I would prefer to fill my car with fuel than walking, despite that we are making our bodies not to be physically active.* (Male, 50, urban, no health condition reported)



For peer review only - http://bmjopen.bmj.com/site/about/guidelines.xhtml

Importantly, despite alternative (less active) forms of transport being less available outside towns and cities in Malawi, there was evidence of the existence of a similar preference among rural participants:

> *Nowadays it's different from the past, in the past our parents could walk by foot from here to South Africa but currently if I am going to Jetty [less than 5 km] I prefer to use a car.* (Male, 55, rural, hypertension reported)

Therefore, although both men and women living in urban areas appear to walk less in their daily lives, a desire to use other forms of transport (where possible) is also apparent among rural Malawians.

*Recreational physical activity*

Participants in both rural and urban communities reported involvement in a range of recreational physical activities, including traditional dancing (apart from urban men); jogging (although this was infrequently reported by rural women), cycling for urban men (rural men only reported cycling for transport), playing with their children (mainly women), and team sports (e.g., netball for women and football for men). Football was highly popular among both urban and rural men, and its importance as both a recreational and social activity is illustrated by this account from one younger rural man describing how he and his friends often improvise so they can enjoy a game of football after work:

> *During rainy season we like playing football in a club but in the summer season we do farming in marsh gardens and after that we play football using a ball made from plastic papers and rubbers.* (Male, 24, rural, no health condition reported)



For peer review only

A number of urban (but not rural) participants described going to the gym or attending exercise classes. As one woman highlighted, involvement in formal types of exercising helped to compensate for the largely inactive lifestyles that people living in towns and cities often led.

> *… here in town we find gym to be our solution in making our bodies active while in the villages they work hence making their bodies active....* (Female, 60, urban, hypertension reported)

Our analysis therefore suggests that many Malawians already undertake recreational physical activities, even in rural areas, where levels of physical activity are generally high. Importantly, some among urban participants recognize the need to do recreational physical activity as other types of physical activity decline.

**General decline in physical activity in Malawi over time**

In general, people living in rural areas reported doing more strenuous physical activity within the home and at work, and also walking more than those living in the city. Most participants also felt that physical activity levels within the general population were decreasing in Malawi due to technological advancements including mechanical maize mills, farming tools/machines and mobile phones. Almost all were aware that being less active placed them at a disadvantage compared to previous generations:

> *Our parents were walking long distances with heavy luggage on their heads, up to 10 km on foot, even farming they could do by themselves, without employing other people to help them but nowadays we can't manage all these. Our bodies are weak while our parents were strong.* (Female, 30, urban, no health condition reported)



For peer review only - http://bmjopen.bmj.com/site/about/guidelines.xhtml

The seemingly inevitable decline in household, occupational and transport-related physical activity, suggests the promotion of physical activity (including recreational) should become a public health priority in Malawi. In order to develop effective strategies to encourage people to be more active, it is important to understand what supports people in Malawi to be physically active, and what might prevent them from doing so.

## Factors influencing physical activity in Malawian adults

*Individual-level influences on physical activity*

Participants in all focus groups recognized that being physically active was important for maintaining their general health and wellbeing. Some specifically described how physical activity was good for releasing tension, blood circulation and maintaining flexibility. These views were particularly common among rural women.

> *If the blood wasn't moving properly, the exercise helps the blood to move very well.* (Female, 56, rural, diabetes reported)

As the above extract suggests, there was clear recognition of the direct health benefits of being active. Discussions within all focus groups provided specific examples of participants undertaking physical activity to either prevent future illnesses or improve an existing health condition:

> *As for me I started to do exercises regularly when I was told that I am pre-diabetic* [following participation in the 2013-16 NCD survey] *and each time I go to the hospital diabetes [blood sugar] is high[er] - this motivated me to do more exercises to reduce my BP and blood sugar level.* (Female, 58, urban, hypertension reported)



For peer review only - http://bmjopen.bmj.com/site/about/guidelines.xhtml

The importance of staying strong and avoiding weakness or frailty was articulated in all focus groups. Most participants recognized and valued the role of physical activity in gaining and maintaining physical strength:

> *When a person is doing physical exercise, the body gains strength; if the body is weak and doesn't feel good, but after doing exercise the body feels well.* (Female, 64, rural, no health condition reported)

Physical activity was also viewed by a few men and women from both urban and rural focus groups as a way of losing weight. Some described their personal experience of weight loss (and associated health benefits) as a result of being more physically active:

> *Before I was diagnosed with diabetes, I was overweight but after the physical activities I do activities such as; digging, working in rice farm, and I found that my weight has been reduced but also sugar level has gone down and I was told not to continue testing my sugar. So, I realize that it's because of exercises.* (Male, 67, rural, diabetes and hypertension reported)

Despite a clear recognition of the potential health benefits of physical activity, a number of factors emerged as getting in the way of people being more physically active. Urban men in particular complained how the multiple, competing pressures they experienced in their daily lives left little time room for recreational physical activity. As one participant suggested, making money is the number one priority for many men living in towns and cities:

> *Being physically active is a very good thing and it helps a lot of things in our bodies, but it seems here in town we are busy making money. Taking care of family and feeding our parents make us forget everything about ourselves, we*



For peer review only - http://bmjopen.bmj.com/site/about/guidelines.xhtml

*can't have time to do exercises, which is very bad.* (Male, 50, urban, no health condition reported)

The negative impact of work and family responsibilities on physical activity was a dominant discourse in the urban focus groups, and the importance of family responsibilities also emerged in the rural focus groups. However, as livelihood and physical activity are still often closely linked in rural communities, as one man had experienced, family responsibilities may lead to increased (rather than reduced) opportunities for physical activity:

*In the past I was leaving everything for my father to do, while I was just staying, but after some time he was diagnosed with high blood pressure and other diseases. So I said to my father that he should not work anymore but I should be the one doing physical work while he should be resting.* (Male, 31, rural, no health condition reported)

The above extract illustrates a prevalent view among many participants that age and ill health were barriers to being physically active. Reports of decreased physical activity with age were common in all focus groups, and some older participants described how fear of injury prevented their involvement in some recreational activities they viewed as high risk:

*All these things we do at home because we are aged and we can't participate in netball which the young ladies are doing. We can't play with them because we fear to be pulled down.* (Female, 60, rural, hypertension reported)



For peer review only - http://bmjopen.bmj.com/site/about/guidelines.xhtml

Although some participants cited specific barriers to being physically active, such as work and family commitments, age and fear of injury, others reflected that they had no real excuse:

> *it's almost a year now without going to gym this has really made me not to be physically active … I have just developed laziness, I don't know why, I used to do press ups, lifting weight, but now I can't do that anymore.* (Male, 38, urban, no health condition reported)

Interestingly, this tendency to blame oneself for being "lazy" was evident across all focus groups, even among rural men and women, where daily physical activity levels were often extremely high.

*Interpersonal-level influences on physical activity*

As reported above, wealth generation emerged as a priority among the urban focus groups, with people feeling they had little time for recreational physical activity. It was also clear that having money in Malawi is associated with societal expectations that further limit people's opportunities for physical activity. First, people with means tend to employ other people to do the physical work (e.g., household chores and farming) that they and their family previously had to do for themselves. This trend was particularly evident in the urban discussions:

> *… maybe the only difference [from people living in rural areas] is farming, they use their own hands… we living here in town we will search for piece-work [casual work] men to do that for us and then pay them later, that's why our life seem to be weak.* (Female, 34, urban, no health condition reported)



For peer review only - http://bmjopen.bmj.com/site/about/guidelines.xhtml

Second, it was evident that walking from place to place is not something that Malawians who can afford other forms of transport do through choice. One urban man clearly described how as he earned more money, the amount of physical activity he expended in getting to work gradually declined:

> *I was walking to and from [work], then I got a promotion and I started using public transport when going to work, because I had managed to get another promotion I started going to work using company car and now that I have my own car I use it as well. I have completely forgotten walking, just because I have money and everything and this has contributed to my low level of physical activity.* (Male, 50, urban, no health condition reported)

Similarly, rural women described how being able to take their maize for processing at the local mill, instead of doing it themselves at home, was a symbol of their social standing:

> *…at present I can take 50 kgs of maize and start pounding but people will get surprised of me why I am doing such activity while am having maize mill closer to me, so instead of doing such activity I try hard to get money and take the maize to the maize mill.* (Female, 56, rural, diabetes reported)

Expectations of what other people would think or say also emerged as a strong influence on the amount and types of physical activity people did, particularly among urban participants. One man described how being made fun of had put him off cycling:

> *I take my bike to the market, people laugh at me sometimes because I am overweight and when they see me riding a bicycle they say I will break the bike… because they're used in seeing me in the car. What people say it can*



For peer review only - http://bmjopen.bmj.com/site/about/guidelines.xhtml

*discourage you from doing exercises*. (Male, 23, urban, no health condition reported)

Some urban women described how gender norms limited the types of physical activity that was appropriate for women to do, and how the predominantly male clientele in many gyms made them feel uncomfortable:

*In [local gym] women cannot manage it is full of the youths and men.* (Female, 49, urban, no health condition reported)

Children were reported as having both negative and positive influences on people's (mainly women's) physical activity. For example, one rural woman reported how neighbours questioned why she continued to fetch water from the well, when she had children to do it for her. Other women recounted experiences of children laughing when they engaged in recreational physical activity. However, as the following extract illustrates, this did not necessarily prevent them from doing it, but at home rather than in public:

*Sometimes, we feel shy to go to the school ground to do sports because of our small children who get surprised when we are playing by mocking us "mum is playing", so you can also do this at home.* (Female, 56, rural, diabetic)

In addition, many rural women spoke about how their children and grandchildren provided an opportunity for recreational physical activity by joining them in playing sports and other (traditional) games:

*I invite my grandchildren to play Jingle* [traditional physical game] *because if I just sit down I feel like my legs are tied up, so I jump a little bit and my*



For peer review only - http://bmjopen.bmj.com/site/about/guidelines.xhtml

*grandchildren also do the same.* (Female, 67, rural, no health condition reported)

Finally, involvement in team sports (e.g., football, netball) organized by local community groups, including churches, was viewed as very beneficial. One urban woman said that knowing other people were depending on her was far more motivational than doing physical activity alone:

*I think doing on our own is what makes us fail to exercise. Whenever we feel tired we stop the routine, but creating groups in our locations can strengthen the desire of doing exercises. If we can just be 15 we can always be meeting, and one may feel bad to be absent a single day because you feel your friends will be missing your part if it is a team.* (Female, 49, urban, no health condition reported)

Therefore, our analysis shows that although other people often had a negative influence on people's physical activity, social forms of recreational physical activity were clearly helpful in encouraging people to be active in both urban and rural settings. Other suggestions to promote recreational and other types of physical activity were mainly targeted at an individual level. These included providing more information on the benefits of physical activity and making physical activity part of normal routine (in both urban and rural focus groups), promoting good time management (particularly in urban focus groups), and importantly, ensuring that people find activities they enjoy doing.

*What the heart has liked, it can't be stopped. Whenever you wake up you will do it.* (Female, 42, rural, hypertension reported)



For peer review only - http://bmjopen.bmj.com/site/about/guidelines.xhtml

## DISCUSSION

This qualitative study demonstrates that men and women previously identified as having pre-diabetes in rural Malawi tend to engage in more vigorous physical activity in their daily lives than their urban counterparts. Recreational physical activities were practiced in both settings, but tended to focus around social interaction and having fun in the rural setting, whilst there was a move towards more structured forms of exercise and gym attendance in the urban setting. Involvement in recreational physical activities reflected a recognition of the need to remain active as the amount of physical activity involved in everyday living decreases in Malawi. Indeed, the understanding that physical activity is important for maintaining good health was widespread, and there was a clear recognition from both men and women of the role of physical activity in maintaining strength. However, aging and ill health emerged as perceived barriers to being active across all focus groups, and these were compounded by lack of time due to competing priorities, wealth accumulation and negative social influences, particularly in the urban setting. Nevertheless, participants were critical of their own and others' perceived "laziness" and highlighted the importance of doing things with other people (including children) to motivate them to do recreational physical activity.

The expected finding that rural participants described doing more vigorous physical activity than urban participants echoes the results of a previous focus group study with men and women in urban and rural Uganda.[24] It reflects in part the nature of work people are involved in, with most rural occupations, such as farming and fishing, involving heavy physical effort. In addition, the growth of motorized transportation in cities, already evident in more developed sub-Saharan Africa countries like South Africa,[35] now also appears to be negatively impacting on physical activity in urban



For peer review only - http://bmjopen.bmj.com/site/about/guidelines.xhtml

499 Malawi. As Malawi continues to develop economically and personal wealth increases,[36 37] it is likely that (in part due to social norms) requirements for occupational, transport and domestic physical activity will reduce,[38] meaning that recreational physical activity will become increasingly important to help people maintain healthy levels of physical activity.[39] The current study revealed that people in Malawi are already involved in a range of recreational physical activities; however, urban residents seemed more reliant on structured, facility-dependent exercising than rural residents. These findings suggest the benefits of less formal (and potentially more social) types of physical activity, such as dancing, walking and being active with family members[21 40-43] should be promoted within urban areas in Malawi.

509 Gender differences in type of physical activity, in particular women doing more household physical activity than men, reflects the fact that the traditional gender divide, with women bearing the main responsibility for domestic and family activities, remains in Malawian society.[44] Interestingly, however, the role of physical activity in maintaining strength as people age was recognized by women as well as men. The importance of undertaking regular strength-based physical activity is gaining prominence in developed countries. For example, the latest 2019 UK physical activity guidelines for adults now prioritize the recommendation to do exercises to build muscle, bone and joint strength at least twice a week[45] (in previous UK guidance the main messaging centered around accumulating at least 150 minutes moderate and vigorous intensity physical activity, and strength exercises were secondary[46]). The value of strength based-exercises in maintaining functionality and avoiding frailty,[47] is likely to be more salient (and therefore potentially motivational) in Malawi, where as elsewhere in sub-Saharan Africa, older people often play a central role in bringing up children and



For peer review only - http://bmjopen.bmj.com/site/about/guidelines.xhtml

running small holdings, where younger family members have moved to seek work in cities, for example.[48]

Both men and women in the current study reported a desire to lose weight as a motivator for physical activity. This finding is interesting given the body of previous research indicating that a larger body size is socially desirable among men and women in Malawi and other sub-Saharan African countries,[49 50] and that lower body weights are often associated with HIV/AIDS and other diseases, stress and poverty.[24 51 52] The recognition that physical activity aids weight management may suggest that in Malawi, as elsewhere in sub-Saharan Africa,[23 53] Westernization is leading to a shift from traditional weight preferences to a smaller body size. An alternative explanation may be the fact that their exposure to health promotion information through involvement in previous health research encouraged our participants to provide views that would be socially acceptable from a public health perspective. Focus group with people with suspected diabetes in Uganda, who may also have been exposed to health messaging, similarly revealed negative views about obesity, whereas focus groups with people without diabetes viewed excess body weight as a sign of success.[24]

Many of the barriers to being physically active reported here, were similar to those found in previous studies in Sub-Saharan Africa. These include negative social influences and lack of social support,[20-22 26] lack of time, [20 21 23] and fear of injury.[20] In South Africa, issues around personal safety and crime emerged as major barriers to physical activity, particularly for women.[21 22] However, participants in the current study presented no such concerns. This may reflect the fact Malawi currently experiences lower crime rates than South Africa and most other sub-Saharan African countries,[54]



For peer review only - http://bmjopen.bmj.com/site/about/guidelines.xhtml

**Table 2 Strategies to improve physical activity levels in Malawi**

| Ecological model level | Barrier/opportunity | Strategy |
|---|---|---|
| Individual | **Barriers** | |
| | • Ill health and fear of injury<br>• Age | 1. Information on health benefits of physical activity.[2 55]<br>2. Information on exercising safely, including as people age.[2 56 57] |
| | • Time constraints due to competing priorities | 3. Promoting forms of physical activity that can fit into daily routines i.e., walking.[56-58] |
| | **Opportunities** | |
| | • Maintaining health and wellbeing<br>• Losing weight<br>• Maintaining strength | See 1 and 2 |
| | • Willingness to exercise at home | 4. Information on easy to do exercises, which can be done alone without support. [56 57] |
| | • Recognition of importance of enjoyment | 5. Promotion of a range of activities (including offering taster sessions) to allow people to find something they enjoy doing.[2 58] |
| Interpersonal | **Barriers** | |
| | • Discouragement (including gender-based) from others<br>• Wealth-related societal expectations | 6. Information campaigns to normalize and promote the value of different forms of physical activity, including for both women and men.[2 55] |
| | • Family responsibilities | 7. Encourage family involvement in physical activity to motivate each other. [55 59]<br>See also 3 and 4 |
| | **Opportunities** | |
| | • Playing with children | 8. Promote the benefits (including bonding) associated with exercising with children.[60] |
| | • Recognition of the value of group-based activities | 9. Encourage group based physical activity.[56-58]<br>See also 5, 7 and 8 |
| Environment | **Barriers** | |
| | • Gyms dominated by men | 10. Set aside female only time slots in gyms.[57]<br>11. Invest in recreational infrastructure such as parks and gyms accessible by both men and women. [2 39 57]<br>12. Encourage people (including those in urban areas) to exercise outdoors using footpaths and parks.[39]<br>See also 6 |
| | **Opportunities** | |
| | • Churches, schools, community organizations | 13. Encourage church, school and community facilities to promote (social) physical activities.[2 57] |
| | • People engage in sedentary activities at workplaces | 14. Workplace initiatives to improve physical activity (e.g., walking breaks; gym membership) with incentives for participation.[2 39 55 56 58 61]<br>15. Encourage people to park a walking distance from workplace.[56] |
| Policy | **Barriers** | |
| | • Use of private cars | 16. Improve quality and accessibility of public transport systems. [57]<br>17. Improve availability of footpaths and cycling and other active transport infrastructure. [2 39 55 57 58 62] |



For peer review only - http://bmjopen.bmj.com/site/about/guidelines.xhtml

546 but also that our participants were often older and perhaps felt less at risk than young
547 women and school-aged girls might have done.[63-65]

548 Importantly, almost all participants recognized the need to improve physical activity in
549 Malawi. Table 2 summarizes potential strategies at different levels of the ecological
550 model of physical activity[33 34] for consideration in future interventions. The strategies
551 reflect the barriers and opportunities identified by participants and previous evidence
552 on the effectiveness of embedding physical activity within daily routines,[56] highlighting
553 the personal benefits/value of physical activity,[2 55] group based exercising,[59] and
554 promoting workplace based physical activity.[39 61 66]

555 To our knowledge, this is the first in-depth exploration of urban/rural and gender
556 differences in physical activity among Malawian adults, and, as such, provides
557 important new evidence of men and women's experiences of, and attitudes towards,
558 physical activity in Malawi. However, the study is not without limitations. Importantly,
559 the participants had all been told they were at high risk of developing diabetes following
560 their participation in previous health research. They had all been advised to attend
561 their local health clinic or hospital, where they may have received information about
562 the importance of physical activity for their health. They may therefore have been more
563 likely to try to include different forms of physical activity in their daily lives than the
564 general adult population of Malawi.

## 565 CONCLUSION

566 This study provides detailed insights into the physical activity views and practices of
567 men and women in urban and rural Malawi, which are important to inform future
568 interventions to increase physical activity. The reported decline in routine physical
569 activity is likely to continue as Malawi undergoes social and economic transition,



For peer review only - http://bmjopen.bmj.com/site/about/guidelines.xhtml

highlighting the need to promote recreational forms of physical activity. The findings suggest that in addition to information campaigns to raise awareness of the benefits of physical activity for all ages, the importance of finding forms of physical activity that people enjoy and the value of (and opportunities for) being physically active with other people, including children and grandchildren, should also be promoted. Finally, public investment in recreational facilities that are fully accessible for women, as well as men, and in active travel infrastructure will also be required to ensure that healthy levels of physical activity continue to be part of everyday life in Malawi.

## Acknowledgments

The authors would like to thank: Hazel Namadingo for her input while employed by MEIRU in designing the topic guide, conducting the focus groups in Lilongwe and quality assuring all transcripts; Cecelia Nyirenda for conducting the focus groups and undertaking the transcription and translation in Karonga; and Maisha Nyasulu for the transcription and translation in Lilongwe.

## Contributors

CMG, CB, JMG and MC designed and conducted the study and acquired the data. JB, CMG and CB conceived and designed the analyses. JB and CMG interpreted the data and contributed to drafting the manuscript. All authors contributed to revising the manuscript critically for important intellectual content and all authors approved the version of the manuscript to be published.

## Funding

This study was funded by the Medical Research Council (MR/R019428/1). The funder had no influence on the analysis plan, results presented or decision to publish.



For peer review only - http://bmjopen.bmj.com/site/about/guidelines.xhtml

For peer review only

593 **Competing interests**

594 None declared.

595 **Participant consent for publication**

596 Not required.

597 **Ethics approval**

598 The study was approved by the National Health Sciences Research Committee in
599 Malawi (18/01/1951) and University of Glasgow College of Medical, Veterinary and
600 Life Sciences Ethics Committee (200170173) in the UK. All participants provided
601 written informed consent prior to their participation in the focus groups.

602 **Data availability statement**

603 Data are available on request from the corresponding author

For peer review only



For peer review only - http://bmjopen.bmj.com/site/about/guidelines.xhtml

## REFERENCES

1. World Health Organization. Noncommunicable Diseases Progress Monitor 2020: World Health Organization, 2020.

2. World Health Organization. Physical Activity Factsheet. 2018. Available: https://www.who.int/en/news-room/fact-sheets/detail/physical-activity. [accessed January 2020].

3. Darling AM, Sunguya B, Ismail A, et al. Gender differences in nutritional status, diet and physical activity among adolescents in eight countries in sub-Saharan Africa. *Trop Med Int Health* 2020;**25**(1):33-43.

4. Delisle H, Ntandou-Bouzitou G, Agueh V, et al. Urbanisation, nutrition transition and cardiometabolic risk: the Benin study. *Br J Nutr* 2012;**107**(10):1534-44.

5. Kirunda BE, Wamani H, Fadnes LT, et al. Objectively Assessed Physical Activity and Associated Factors Among Adults in Peri-Urban and Rural Eastern Uganda: A Population-based Study. *J Phys Act Health* 2016;**13**(11):1243-54.

6. Barr AL, Young EH, Sandhu MS. Objective measurement of physical activity: improving the evidence base to address non-communicable diseases in Africa. *BMJ Glob Health* 2018;**3**(5):e001044.

7. World Health Organization. Noncommunicable diseases country profiles 2011: World Health Organization, 2011.

8. World Health Organization. Noncommunicable diseases country profiles 2018: World Health Organization, 2018.

9. Divala OH, Amberbir A, Ismail Z, et al. The burden of hypertension, diabetes mellitus, and cardiovascular risk factors among adult Malawians in HIV care: consequences for integrated services. *BMC Public Health* 2016;**16**(1):1243-43.



For peer review only - http://bmjopen.bmj.com/site/about/guidelines.xhtml

10. Msyamboza KP, Ngwira B, Dzowela T, et al. The burden of selected chronic non-communicable diseases and their risk factors in Malawi: nationwide STEPS survey. *PLoS One* 2011;**6**(5):e20316.

11. World Health Organization. Insufficient physical activity: prevalence of insufficient physical activity among adults. 2018. Available: http://gamapserver.who.int/gho/interactive_charts/ncd/risk_factors/physical_inactivity/atlas.html?indicator=i1&date=Male. [accessed March 2020].

12. Price AJ, Crampin AC, Amberbir A, et al. Prevalence of obesity, hypertension, and diabetes, and cascade of care in sub-Saharan Africa: a cross-sectional, population-based study in rural and urban Malawi. *Lancet Diabetes Endocrinol* 2018;**6**(3):208-22.

13. Soares ALG, Banda L, Amberbir A, et al. Sex and area differences in the association between adiposity and lipid profile in Malawi. *BMJ Glob Health* 2019;**4**(5):e001542-e42.

14. Bigna JJ, Noubiap JJ. The rising burden of non-communicable diseases in sub-Saharan Africa. *Lancet Glob Health* 2019;**7**(10):e1295-e96.

15. Gowshall M, Taylor-Robinson SD. The increasing prevalence of non-communicable diseases in low-middle income countries: the view from Malawi. *Int J Gen Med* 2018;**11**:255-64.

16. World Health Organization. Projections of mortality and causes of death, 2016 to 2060. 2018. Available: https://www.who.int/healthinfo/global_burden_disease/projections/en/. [accessed January 2020].

17. Anderson E, Durstine JL. Physical activity, exercise, and chronic diseases: A brief review. *Sports Med Health Sci* 2019;**1**(1):3-10.



For peer review only - http://bmjopen.bmj.com/site/about/guidelines.xhtml

18. Warburton DER, Nicol CW, Bredin SSD. Health benefits of physical activity: the evidence. *Can Med Assoc J* 2006;**174**(6):801.

19. Milton K, Macniven R, Bauman A. Review of the epidemiological evidence for physical activity and health from low- and middle-income countries. *Glob Public Health* 2014;**9**(4):369-81.

20. Tuakli-Wosornu YA, Rowan M, Gittelsohn J. Perceptions of physical activity, activity preferences and health among a group of adult women in urban Ghana: a pilot study. *Ghana Med J* 2014;**48**(1):3-13.

21. Watson ED, Norris SA, Draper CE, et al. "Just because you're pregnant, doesn't mean you're sick!" A qualitative study of beliefs regarding physical activity in black South African women. *BMC Pregnancy Childbirth* 2016;**16**(1):174-74.

22. Ware LJ, Prioreschi A, Bosire E, et al. Environmental, Social, and Structural Constraints for Health Behavior: Perceptions of Young Urban Black Women During the Preconception Period-A Healthy Life Trajectories Initiative. *J Nutr Educ Behav* 2019;**51**(8):946-57.

23. Phillips EA, Comeau DL, Pisa PT, et al. Perceptions of diet, physical activity, and obesity-related health among black daughter-mother pairs in Soweto, South Africa: a qualitative study. *BMC Public Health* 2016;**16**(1):750.

24. Mayega RW, Etajak S, Rutebemberwa E, et al. 'Change means sacrificing a good life': perceptions about severity of type 2 diabetes and preventive lifestyles among people afflicted or at high risk of type 2 diabetes in Iganga Uganda. *BMC Public Health* 2014;**14**(1):864.

25. Ogunrinu T, Gamboa-Maldonado T, Ngewa RN, et al. A qualitative study of health education experiences and self-management practices among patients with type 2

For peer review only

diabetes at Malamulo Adventist Hospital in Thyolo District, Malawi. *Malawi Med J* 2017;**29**(2):118-23.

26. Mphwanthe G, Carolan M, Earnesty D, et al. Perceived barriers and facilitators to diet and physical activity among adults diagnosed with type 2 diabetes in Malawi. *Glob Public Health* 2021;**16**(3):469-84.

27. Malambo P, Kengne AP, Lambert EV, et al. Prevalence and socio-demographic correlates of physical activity levels among South African adults in Cape Town and Mount Frere communities in 2008-2009. *Arch Public Health* 2016;**74**:54-54.

28. Guthold R, Louazani SA, Riley LM, et al. Physical activity in 22 African countries: results from the World Health Organization STEPwise approach to chronic disease risk factor surveillance. *Am J Prev Med* 2011;**41**(1):52-60.

29. Hoare E, Stavreski B, Jennings GL, et al. Exploring Motivation and Barriers to Physical Activity among Active and Inactive Australian Adults. *Sports (Basel)* 2017;**5**(3):47.

30. Chaabane S, Chaabna K, Abraham A, et al. Physical activity and sedentary behaviour in the Middle East and North Africa: An overview of systematic reviews and meta-analysis. *Sci Rep* 2020;**10**(1):9363.

31. Braun V, Clarke V. Successful Qualitative Research: a practical guide for beginners. London: SAGE, 2013.

32. Braun V, Clarke V. Using thematic analysis in psychology. *Qual Res Psychol* 2006;**3**:77-101.

33. Sallis JF, Owen N, Fisher EB. Ecological Models of Health Behaviours. In: Glanz K, Rimer BK, Viswanath K, eds. Health Behaviour and Health Education: Theory, Research and Practice. 4th ed. San Francisco: Jossey-Bass, 2008:465-85.



For peer review only - http://bmjopen.bmj.com/site/about/guidelines.xhtml

34. Bauman AE, Reis RS, Sallis JF, et al. Correlates of physical activity: why are some people physically active and others not? *Lancet* 2012;**380**(9838):258-71.

35. Luke R. Car ownership perceptions and intentions amongst South African students. *J Transp Geogr* 2018;**66**:135-43.

36. National Planning Commission. Malawi's vision: An inclusively wealthy and self-reliant nation. Lilongwe, Malawi: National Planning Commission, 2020.

37. Government of Malawi. Malawi Growth and Development Strategy III: Building a Productive, Competitive and Resilient Nation. Lilongwe, Malawi: Government of Malawi, 2017.

38. Ng SW, Popkin BM. Time use and physical activity: a shift away from movement across the globe. *Obes Rev* 2012;**13**(8):659-80.

39. Guthold R, Stevens GA, Riley LM, et al. Worldwide trends in insufficient physical activity from 2001 to 2016: a pooled analysis of 358 population-based surveys with 1.9 million participants. *Lancet Glob Health* 2018;**6**(10):e1077-e86.

40. Chaabane S, Chaabna K, Doraiswamy S, et al. Barriers and Facilitators Associated with Physical Activity in the Middle East and North Africa Region: A Systematic Overview. *J Environ Res Public Health* 2021;**18**(4).

41. Harrison AL, Taylor NF, Shields N, et al. Attitudes, barriers and enablers to physical activity in pregnant women: a systematic review. *J Physiother* 2018;**64**(1):24-32.

42. Saligheh M, McNamara B, Rooney R. Perceived barriers and enablers of physical activity in postpartum women: a qualitative approach. *BMC Pregnancy Childbirth* 2016;**16**(1):131.



For peer review only - http://bmjopen.bmj.com/site/about/guidelines.xhtml

43. Beune EJAJ, Haafkens JA, Agyemang C, et al. Inhibitors and enablers of physical activity in multiethnic hypertensive patients: qualitative study. *J Hum Hypertens* 2010;**24**(4):280-90.

44. National Statistical Office, ICF. Malawi Demographic and Health Survey 2015-16. Zomba, Malawi, and Rockville, Maryland, USA: NSO and ICF, 2017.

45. Department of Health and Social Care. UK Chief Medical Officers' Physical Activity Guidelines. UK: Department of Health and Social Care, 2019:66.

46. Department of Health and Social Care. Start Active, Stay Active: A report on physical activity from the four home countries' Chief Medical Officers, 2011.

47. Hillsdon M, Foster C. What are the health benefits of muscle and bone strengthening and balance activities across life stages and specific health outcomes? *J Frailty Sarcopenia Falls* 2018;**3**(2):66-73.

48. Aboderin IAG, Beard JR. Older people's health in sub-Saharan Africa. *Lancet* 2015;**385**(9968):e9-e11.

49. Flax VL, Thakwalakwa C, Phuka JC, et al. Body size preferences and food choice among mothers and children in Malawi. *Matern Child Nutr* 2020;**16**(4):e13024.

50. Okoro EO, Oyejola BA, Etebu EN, et al. Body size preference among Yoruba in three Nigerian communities. *Eat Weight Disord* 2014;**19**(1):77-88.

51. Matoti-Mvalo T, Puoane T. Perceptions of body size and its association with HIV/AIDS. *South Afr J Clin Nutr* 2011;**24**(1):40-45.

52. Puoane T, Tsolekile L, Steyn N. Perceptions about body image and sizes among Black African girls living in Cape Town. *Ethn Dis* 2010;**20**(1):29-34.

53. Benkeser RM, Biritwum R, Hill AG. Prevalence of overweight and obesity and perception of healthy and desirable body size in urban, Ghanaian women. *Ghana Med J* 2012;**46**(2):66-75.

For peer review only



For peer review only - http://bmjopen.bmj.com/site/about/guidelines.xhtml

54. Institute for Economics and Peace. Global Peace Index 2020: Measuring Peace in a Complex World. Sydney, 2020.

55. Tuso P. Strategies to Increase Physical Activity. *Perm J* 2015;**19**(4):84-88.

56. Centers for Disease Control and Prevention. Overcoming Barriers to Physical Activity. 2020. Available: https://www.cdc.gov/physicalactivity/basics/adding-pa/barriers.html. [accessed September 2020].

57. Heath GW, Parra DC, Sarmiento OL, et al. Evidence-based intervention in physical activity: lessons from around the world. *Lancet* 2012;**380**(9838):272-81.

58. Ferguson-Stegall L, Robb JD. Effective Strategies to Increase Physical Activity in the Working Years. *ACSMs Health Fit J* 2019;**23**(5).

59. Brandt CJ, Clemensen J, Nielsen JB, et al. Drivers for successful long-term lifestyle change, the role of e-health: a qualitative interview study. *BMJ Open* 2018;**8**(3):e017466.

60. Paquette D, Dumont C. Is father–child rough-and-tumble play associated with attachment or activation relationships? *Early Child Development and Care* 2013;**183**(6):760-73.

61. Centers for Disease Control and Prevention. Worksite Physical Activity. September 28, 2017 2017. Available: https://www.cdc.gov/physicalactivity/worksite-pa/index.htm. [accessed September 2020].

62. Brown DR, Heath GW, Martin SL, editors. *Promoting Physical Activity: A Guide for Community Action*. 2 ed. Champaign, IL: Human Kinetics, 2010.

63. Kayira J. Malawi: Rape, defilement cases horrify a nation. 2020. Available: https://face2faceafrica.com/article/malawi-rape-defilement-cases-horrify-a-nation. [accessed August 2021].



For peer review only - http://bmjopen.bmj.com/site/about/guidelines.xhtml

64. Masiye M. Defilement, Rape Up to 43% in Eastern Region. 2020. Available: https://zodiakmalawi.com/nw/national-news/66-news-in-southern-region/2893-defilement-rape-up-to-43-in-eastern-region. [accessed August 2021].

65. Sapuli D. Malawi has over 2100 child rapists in its prisons. 2020. Available: https://malawi24.com/2020/10/30/malawi-has-over-2100-child-rapists-in-its-prisons/. [accessed August 2021].

66. World Health Organization. Global action plan on physical activity 2018–2030: more active people for a healthier world: World Health Organization, 2018.

For peer review only



For peer review only - http://bmjopen.bmj.com/site/about/guidelines.xhtml

# SUPPLEMENTARY MATERIALS

## Physical activity focus group topic guide

1. Tell me about when you are physically active/do exercise in your daily life. What sort of things do you do? Where? Why? (Prompt for work, travel and leisure activity)

2. How would you say your activity levels compare with your parents/grandparents? Why is this? (Prompt changes to work, leisure and travel – moving to different place)

3. Have your own activity levels changed over the years? Why is this?

4. What are the benefits to you of being physically active? (Prompt: fitness, strength, flexibility) how important are these to you/other people in your community? Why is this?

5. What do you understand by exercise?

6. Do you ever do exercise? What do you do and where? Who do you do it with? What motivates you to do exercise?

7. Tell me about any social/community occasions which involve physical activity/exercise?

8. What do you know about physical activity and health? ([Prompt: Benefits and risks of being physically active, including in relation to diabetes) How do you know this? (Prompt: where did this knowledge come from?)

9. How could you do more physical activity in your daily life? What might stop you doing this?

Demonstrate and lead an exercise session including resistance exercises. Ask the participants what they think of them as they do them.

10. Afterwards, ask: which of these exercises might you do again (if any)? Why have you chosen these? What would help you do these regularly? What would stop you doing them regularly?

11. Which of the exercises would you not like to do again? Why is this?

For peer review only

**Table 1 Description of the 10 broad themes**

| Theme | Description |
|---|---|
| 1. Types of physical activity undertaken | Activities participants reported doing which in turn made them physically active, these included recreational and for survival. |
| 2. Changes in physical activity over time | How physical activities have changed over time from generation to generation, and individually. |
| 3. Physical activity and health | How physical activities influences one's health and how they are affected by one's health |
| 4. Motivations | Captured participant's reasons and motivations for being involved in different physical activities. |
| 5. Barrier and facilitators | Different factors facilitating and/or hindering their involvement in physical activities |
| 6. Social norms | Peoples views and acceptability/rejection of different physical activities |
| 7. Social support | People and social institutions providing support for physical activities |
| 8. Technology and physical activity | How technological advancements have affected physical activities (e.g., transportation, farming machinery, maize mills) |
| 9. Sources of information | Where participant's received their information on physical activities |
| 10. Ways of improving physical activity | Suggested ways of improving physical activity |



For peer review only - http://bmjopen.bmj.com/site/about/guidelines.xhtml

# Standards for Reporting Qualitative Research (SRQR)*

http://www.equator-network.org/reporting-guidelines/srqr/

## Title and abstract

| | Page/line no(s). |
|---|---|
| **Title** - Concise description of the nature and topic of the study Identifying the study as qualitative or indicating the approach (e.g., ethnography, grounded theory) or data collection methods (e.g., interview, focus group) is recommended | Page 1, lines 1-2 |
| **Abstract** - Summary of key elements of the study using the abstract format of the intended publication; typically includes background, purpose, methods, results, and conclusions | Page 2-3, lines 19-47 |

## Introduction

| | |
|---|---|
| **Problem formulation** - Description and significance of the problem/phenomenon studied; review of relevant theory and empirical work; problem statement | Pages 5-7, lines 62-115 |
| **Purpose or research question** - Purpose of the study and specific objectives or questions | Page 7, lines 115-121 |

## Methods

| | |
|---|---|
| **Qualitative approach and research paradigm** - Qualitative approach (e.g., ethnography, grounded theory, case study, phenomenology, narrative research) and guiding theory if appropriate; identifying the research paradigm (e.g., postpositivist, constructivist/ interpretivist) is also recommended; rationale** | Pages 9-10, lines 163, 178-184 |
| **Researcher characteristics and reflexivity** - Researchers' characteristics that may influence the research, including personal attributes, qualifications/experience, relationship with participants, assumptions, and/or presuppositions; potential or actual interaction between researchers' characteristics and the research questions, approach, methods, results, and/or transferability | Pages 8-9, lines 152-157 |
| **Context** - Setting/site and salient contextual factors; rationale** | Pages 7, lines 124-132 |
| **Sampling strategy** - How and why research participants, documents, or events were selected; criteria for deciding when no further sampling was necessary (e.g., sampling saturation); rationale** | Pages 7-8, lines 125-129, 134-138 |
| **Ethical issues pertaining to human subjects** - Documentation of approval by an appropriate ethics review board and participant consent, or explanation for lack thereof; other confidentiality and data security issues | Page 30, lines 597 - 601 |
| **Data collection methods** - Types of data collected; details of data collection procedures including (as appropriate) start and stop dates of data collection and analysis, iterative process, triangulation of sources/methods, and modification of procedures in response to evolving study findings; rationale** | Page 8-9, lines, 134-135, 146-157 |
| **Data collection instruments and technologies** - Description of instruments (e.g., interview guides, questionnaires) and devices (e.g., audio recorders) used for data collection; if/how the instrument(s) changed over the course of the study | Supplementary materials lines 2-28 |
| **Units of study** - Number and relevant characteristics of participants, documents, or events included in the study; level of participation (could be reported in results) | Page 8, lines 135-138. Pages 10-11, lines 188-203, Table 1 |



For peer review only - http://bmjopen.bmj.com/site/about/guidelines.xhtml

| | |
|---|---|
| **Data processing** - Methods for processing data prior to and during analysis, including transcription, data entry, data management and security, verification of data integrity, data coding, and anonymization/de-identification of excerpts | Page 9-10, lines 158-161, 184-186 |
| **Data analysis** - Process by which inferences, themes, etc., were identified and developed, including the researchers involved in data analysis; usually references a specific paradigm or approach; rationale** | Pages 9-10, lines 163-184 |
| **Techniques to enhance trustworthiness** - Techniques to enhance trustworthiness and credibility of data analysis (e.g., member checking, audit trail, triangulation); rationale** | Page 9, lines 159-175 |

**Results/findings**

| | |
|---|---|
| **Synthesis and interpretation** - Main findings (e.g., interpretations, inferences, and themes); might include development of a theory or model, or integration with prior research or theory | Pages 12-23, lines 206-474 |
| **Links to empirical data** - Evidence (e.g., quotes, field notes, text excerpts, photographs) to substantiate analytic findings | Pages 12-23, lines 215-474 |

**Discussion**

| | |
|---|---|
| **Integration with prior work, implications, transferability, and contribution(s) to the field** - Short summary of main findings; explanation of how findings and conclusions connect to, support, elaborate on, or challenge conclusions of earlier scholarship; discussion of scope of application/generalizability; identification of unique contribution(s) to scholarship in a discipline or field | Pages 24-28, lines 475-554 |
| **Limitations** - Trustworthiness and limitations of findings | Page 3, lines 50-58. Page 28, lines 555-566 |

**Other**

| | |
|---|---|
| **Conflicts of interest** - Potential sources of influence or perceived influence on study conduct and conclusions; how these were managed | Pages 29, line 593-594 |
| **Funding** - Sources of funding and other support; role of funders in data collection, interpretation, and reporting | Page 29, lines 590-592 |

*The authors created the SRQR by searching the literature to identify guidelines, reporting standards, and critical appraisal criteria for qualitative research; reviewing the reference lists of retrieved sources; and contacting experts to gain feedback. The SRQR aims to improve the transparency of all aspects of qualitative research by providing clear standards for reporting qualitative research.

**The rationale should briefly discuss the justification for choosing that theory, approach, method, or technique rather than other options available, the assumptions and limitations implicit in those choices, and how those choices influence study conclusions and transferability. As appropriate, the rationale for several items might be discussed together.

**Reference:**

O'Brien BC, Harris IB, Beckman TJ, Reed DA, Cook DA. **Standards for reporting qualitative research: a synthesis of recommendations.** *Academic Medicine*, Vol. 89, No. 9 / Sept 2014
DOI: 10.1097/ACM.0000000000000388



For peer review only - http://bmjopen.bmj.com/site/about/guidelines.xhtml

BMJ Open

# A qualitative study of practices and attitudes towards physical activity among pre-diabetic men and women in urban and rural Malawi

| | |
|---|---|
| Journal: | *BMJ Open* |
| Manuscript ID | bmjopen-2021-058261.R1 |
| Article Type: | Original research |
| Date Submitted by the Author: | 08-Aug-2022 |
| Complete List of Authors: | Banda, Jethro; Malawi Epidemiology and Intervention Research Unit<br>Bunn, Christopher; University of Glasgow, School of Social and Political Sciences, Institute of Health and Wellbeing; Malawi Epidemiology and Intervention Research Unit<br>Crampin, Amelia; London School of Hygiene and Tropical Medicine; Malawi Epidemiology and Intervention Research Unit<br>Gill, Jason; University of Glasgow, Institute of Cardiovascular and Medical Sciences<br>Gray, Cindy; University of Glasgow, School of Social and Political Sciences, Institute of Health and Wellbeing |
| <b>Primary Subject Heading</b>: | Public health |
| Secondary Subject Heading: | Global health, Qualitative research |
| Keywords: | General diabetes < DIABETES & ENDOCRINOLOGY, PUBLIC HEALTH, QUALITATIVE RESEARCH |
| | |

SCHOLARONE™
Manuscripts

For peer review only - http://bmjopen.bmj.com/site/about/guidelines.xhtml

*I, the Submitting Author has the right to grant and does grant on behalf of all authors of the Work (as defined in the below author licence), an exclusive licence and/or a non-exclusive licence for contributions from authors who are: i) UK Crown employees; ii) where BMJ has agreed a CC-BY licence shall apply, and/or iii) in accordance with the terms applicable for US Federal Government officers or employees acting as part of their official duties; on a worldwide, perpetual, irrevocable, royalty-free basis to BMJ Publishing Group Ltd ("BMJ") its licensees and where the relevant Journal is co-owned by BMJ to the co-owners of the Journal, to publish the Work in this journal and any other BMJ products and to exploit all rights, as set out in our licence.*

*The Submitting Author accepts and understands that any supply made under these terms is made by BMJ to the Submitting Author unless you are acting as an employee on behalf of your employer or a postgraduate student of an affiliated institution which is paying any applicable article publishing charge ("APC") for Open Access articles. Where the Submitting Author wishes to make the Work available on an Open Access basis (and intends to pay the relevant APC), the terms of reuse of such Open Access shall be governed by a Creative Commons licence – details of these licences and which Creative Commons licence will apply to this Work are set out in our licence referred to above.*

*Other than as permitted in any relevant BMJ Author's Self Archiving Policies, I confirm this Work has not been accepted for publication elsewhere, is not being considered for publication elsewhere and does not duplicate material already published. I confirm all authors consent to publication of this Work and authorise the granting of this licence.*

For peer review only

# A qualitative study of practices and attitudes towards physical activity among pre-diabetic men and women in urban and rural Malawi

Mr Jethro Banda, Malawi Epidemiology and Intervention Research Unit, Lilongwe, Malawi. Jethro.Banda@meiru.mw

Dr Christopher Bunn PhD, School of Social and Political Sciences, Institute of Health and Wellbeing, University of Glasgow, UK and Malawi Epidemiology and Intervention Research Unit, Lilongwe, Malawi. Christopher.Bunn@glasgow.ac.uk

Professor Amelia Crampin MBChB DTM&H MSc FFPHM, Malawi Epidemiology and Intervention Research Unit, Lilongwe, Malawi, Institute of Health and Wellbeing, University of Glasgow, UK and London School of Hygiene and Tropical Medicine, London, UK. Mia.Crampin@lshtm.ac.uk

Professor Jason MR Gill PhD, Institute of Cardiovascular and Medical Sciences, University of Glasgow, UK. Jason.Gill@glasgow.ac.uk

Professor Cindy M Gray* PhD, School of Social and Political Sciences, Institute of Health and Wellbeing, University of Glasgow, UK. cindy.gray@glasgow.ac.uk

*Corresponding author



For peer review only - http://bmjopen.bmj.com/site/about/guidelines.xhtml

## ABSTRACT

**Introduction** Malawi, like other Sub-Saharan African countries, is experiencing a decline in physical activity levels, particularly in urban areas. As physical inactivity is associated with increased risk of non-communicable diseases, such as type 2 diabetes, interventions are needed to promote physical activity. This study aimed to generate the in-depth understanding of physical activity practices and attitudes required to inform physical activity interventions in Malawi.

**Methods** Focus groups (N=4) with men and women in urban (men N=6, women N=9) and rural (men N=8, women N=9) Malawi, who had been identified as having pre-diabetes 3-5 years previously. Thematic analysis identified 10 themes relating to physical activity practices and attitudes, and the ecological model was used to characterize different types of physical activity people engaged in and the factors influencing them.

**Results** Participants reported undertaking different types of physical activity across all ecological model domains (household, occupational, transport, recreational). Rural participants reported more vigorous physical activities than urban participants, and women reported more household activities than men. Many participants recognized a need to promote physical activity in Malawi, and the health benefits of doing so, including the importance of physical activity in helping them stay strong to maintain physical functioning. Barriers to being physically active included competing priorities (especially for urban men), societal expectations around wealth, use of motorized transport, lack of accessible facilities for women, aging and ill health.

**Conclusions** Physical activity is declining in Malawi as working and transport practices change in response to economic development, making promotion of




For peer review only - http://bmjopen.bmj.com/site/about/guidelines.xhtml

alternative forms of physical activity a public health priority. Multi-level interventions emphasizing the personal benefits/value of physical activity for all ages, and routine and group-based exercising, as well as investment in accessible recreational facilities (including for women) and active travel infrastructure should be urgently considered to improve physical activity levels in Malawi.

**Keywords** Physical activity, facilitators, barriers, interventions, ecological model, qualitative, Sub-Saharan Africa, Malawi.

**Strengths and limitations of this study**

- This is the first in-depth exploration of physical activity among community-dwelling adults in Malawi.
- Conducting separate focus groups with men and women in urban and rural settings allowed systematic comparison of urban/rural and gender differences in physical activity.
- Participants had previously been told they were at high risk of developing diabetes and may been more informed about the importance of physical activity for their health than the general adult population of Malawi.

For peer review only

| What is already known? |
|---|
| • Increasing levels of physical inactivity among men and women in Malawi are becoming a public health concern as morbidity and mortality from non-communicable diseases increase. |
| **What are the new findings?** |
| • Men and women in both urban and rural Malawi acknowledge the decline in physical activity and possible impact on their health. • Societal pressures, time constraints (particularly for urban men), aging and ill-health emerged as important barriers to being physically active. |
| **What do the new findings imply?** |
| • Increasing physical activity in Malawi requires intervening at multiple levels. • Individual and interpersonal-level initiatives should focus on the personal benefits and value of routine and recreational group-based physical activity • Environmental/policy level investment is needed to increase availability of fully accessible physical activity facilities and active travel infrastructure such as pavements. |

For peer review only

## INTRODUCTION

Lack of physical activity is thought to be the fourth leading risk factor for premature mortality globally.[1] The World Health Organization (WHO) recommends that adults undertake 150 minutes of moderate-intensity or 75 minutes of vigorous-intensity physical activity a week for optimal health.[2] However, a quarter of adults fail to meet these guidelines worldwide.[2] In Sub-Saharan Africa, physical activity levels have historically been high, but increased urbanization and adoption of Westernized lifestyles mean physical inactivity is increasing.[3-6] In Malawi, the most recent WHO country profiles published in 2011 and 2018 demonstrate that the prevalence of physical inactivity rose from 10% to 14% in the eight years to 2016.[7,8] In addition, some studies have reported lower physical activity levels in Malawi's urban areas,[9] with one population survey reporting that 24% of urban residents were inactive compared with 9% of rural residents.[10] Gender differences are less clear: while population surveys report that men in Malawi are generally more physically active than women;[10 11] some studies in specific urban and rural settings show that women are more active than men.[12 13]

The prevalence of non-communicable diseases (NCD), such as diabetes, cardiovascular disease, chronic respiratory disease and cancer, is on the rise in Sub-Saharan Africa.[14 15] Deaths from NCD in low and middle income countries are forecast to increase from 31.5 million in 2016 to 41.9 million in 2030 and to 56.6 million by 2045.[16] Recent estimates suggest that 32% of deaths in Malawi are associated with NCD.[1] Although being physically active helps both prevention and management of NCD,[17-19] currently Malawi has no physical activity public health campaigns.[1]

Promotion of behaviour change for physical activity requires detailed understanding of current local practices and attitudes, as well as the different contexts in which physical

For peer review only



For peer review only - http://bmjopen.bmj.com/site/about/guidelines.xhtml

activity is undertaken. However, only a handful of qualitative studies exploring practices and attitudes towards physical activity in Sub-Saharan Africa have been conducted. Interviews and focus group discussions with urban women in Ghana revealed a desire for more social (including group-based) physical activity, and that lack of time and concerns about injury (e.g., doing age-appropriate exercises) were other barriers to being physically active.[20] Three studies with women in urban South Africa[21-23] showed that although participants were aware of the physical and mental health benefits of physical activity (including weight loss); lack of time, money and information, perceptions of local crime, and negative influences from other people, including abuse from men for wearing "exercise" clothing, were barriers to their being more physically active. Focus groups with men and women with suspected type 2 diabetes, suspected pre-diabetes or who were obese in urban and rural Uganda suggested that people were willing to become more active to improve their health, and some described doing this by embedding physical activity within their normal routines.[24] This included washing, cooking, walking and cycling in urban areas, and more vigorous activities such as gardening, bush clearing, fetching water, splitting firewood and pounding food in rural areas. However, any gender differences were not explored.

Two qualitative studies on physical activity have been conducted in Malawi in men and women with diabetes. In one study, interviews and focus groups with diabetes patients attending health education classes at a rural private hospital revealed that people were aware of the importance of both routine and leisure (recreational) physical activity for their health; however, no gender comparisons were reported.[25] In a separate study, focus group discussions with patients in urban and semi-urban areas reported both positive and negative social influences. Specifically, while ridicule from other people



For peer review only - http://bmjopen.bmj.com/site/about/guidelines.xhtml

hindered their participation in some types of physical activity, encouragement from health workers and family members (including taking part in household activities) helped them remain active.[26]

Despite declining physical activity levels[6 15] and the implication of this for NCD prevention and management,[17-19] there is currently very little evidence about the practices and attitudes of adults in Malawi towards physical activity. As physical activity (and its determinants) may differ according to gender and place of residence,[2 27-30] the aim of this qualitative study was to compare and contrast accounts of practices and attitudes towards physical activity among Malawian men and women in urban and rural settings who had previously been identified as having pre-diabetes. This new evidence will be useful in informing the development of effective physical activity interventions in Malawi.

## METHODS

### Study design

To elicit accounts of physical activity in everyday life among urban and rural Malawians living with pre-diabetes, we used a focus group-based research design. Focus groups were chosen to enable participants to develop rich accounts of physical activity in their communities in an interactive setting that encouraged them to elaborate and examine the narratives offered by fellow participants.[31] This approach was framed within a pragmatist philosophy,[32] which positions participants' narratives as neither objects of knowledge which enable prediction nor as the 'truth' of those the participants. Rather, the narratives are seen as offering insight into the 'vocabularies' of physical activity that are used by participants and which can be engaged with when seeking change.

### Setting and recruitment



For peer review only - http://bmjopen.bmj.com/site/about/guidelines.xhtml

Data collection took place in Lilongwe (urban) and Karonga (rural) in March 2019. Participants were recruited from a sample of adults previously identified (during participation in an NCD survey between 2013 and 2016)[12] as having pre-diabetes. All were part of a larger MRC-funded study (MR/R019428/1; NHSRC protocol 18/01/1951) investigating links between nutritional intake and physical, metabolic and inflammatory phenotypes in pre-diabetic (n=50) and normoglycaemic (n=50) men and women. During the informed consent process for this larger study, participants were asked if they would be willing to participate in a focus group if selected. No refusals were recorded during this process, and participants from the 'pre-diabetic' sub-group were purposively sampled to achieve variation in age.

**Participants**

Four focus groups were held with men and women separately in the urban and rural locations. They included a total of 32 participants (urban N=15, 6 men, 9 women; rural N=17, 8 men, 9 women).  At the time of the current study, some participants reported progression from their pre-diabetic status at the last NCD survey to diabetes and/or hypertension.

**Participant and public involvement**

At the start of the MRC-funded study, stakeholder engagement meetings with community leaders, community members, healthcare professionals, policy makers and patient group were held in both rural and urban locations to raise awareness of the research and gather feedback on the design and implementation of data collection procedures, including the focus group discussions reported here.

**Data collection and management**



For peer review only - http://bmjopen.bmj.com/site/about/guidelines.xhtml

The focus groups lasted between 90 and 120 minutes and were audio recorded with participant consent. A semi-structured topic guide (see Supplementary materials) was developed informed by our pragmatist approach[32] to focus discussion around participants' personal accounts of physical activity in their daily lives, their understandings of physical activity and health (including the benefits of being physically active), perceived barriers and facilitators to being physically active (both personal and among others in their community) and their perceptions of changes in physical activity over time. The topic guide was developed in English by CB, CMG and Hazel Namadingo (HN), all experienced qualitative researchers, then translated into Chichewa for urban focus groups and into Chitumbuka for rural focus groups by fluent Chichewa and Chitumbuka speakers. The urban focus groups were conducted by HN; the rural focus groups were conducted by Cecelia Nyirenda, an experienced field worker. The facilitators were fluent in the local languages and familiar with the issues under discussion.

The audio recordings, together with field notes taken during the focus groups, were transcribed directly into English by experienced field workers fluent in Chichewa and Chitumbuka. HN (native Chichewa speaker) and JB (native Chichewa speaker and fluent in Chitumbuka) then quality assured the transcripts while listening to the audio recordings, and made changes where necessary.

**Data analysis**

The quality-assured transcripts were analyzed using a reflexive thematic approach.[33 34] JB, CMG and CB read through the transcripts line by line to familiarize themselves with the data and identify initial codes relating to participants' accounts, knowledge, barriers and facilitators of physical activity, and any emerging areas of interest (such as changes in physical activity over time in Malawi). The three authors then met to



For peer review only - http://bmjopen.bmj.com/site/about/guidelines.xhtml

discuss and combine their initial codes into 10 broad semantic themes to reflect participants' accounts of physical activity. Two of these described the context of physical activity in Malawi – 1) Types of physical activity undertaken; 2) Changes in physical activity over time. Six described perceived influences on physical activity – 3) Physical activity and health; 4) Motivations for physical activity; 5) Barriers to physical activity; 6) Technology and physical activity; 7) Social norms; 8) Social support. One described potential solutions – 9) Ways of improving physical activity, and a final theme – 10) Sources of information, which is not elaborated in this paper. A detailed description of each broad theme, together with example extracts, is provided in Supplementary materials (Table S1).

JB then applied the broad themes to all transcripts using NVivo11 software to organize the data, and a reflective and iterative approach to identify all data within each theme and consider whether the themes adequately reflected the meanings represented in the data or needed refined (e.g., by identifying 'Other' potentially relevant extracts for inclusion in the analysis). The resultant coded transcripts were reviewed by CMG, any disagreements resolved through discussion and all extracts coded as 'Other' jointly examined to decide whether they represented a new broad theme, belonged to an existing theme or could be discarded. All were either assigned to an existing theme or discarded. JB then further refined the broad themes into sub-themes to produce a detailed narrative account of each broad theme, which included careful reading, re-reading and detailed notetaking using an adapted one sheet of paper (OSOP) approach[35] to compare similarities and differences in accounts between men and women in urban and rural settings, and to capture interactions within the focus groups. The ecological model of physical activity[36 37] was then applied as a framework to characterize the different types of physical activity participants engaged in and the



For peer review only - http://bmjopen.bmj.com/site/about/guidelines.xhtml

factors influencing them. The ecological model constructs physical activity as influenced by interacting factors operating at different levels: individual, interpersonal, environmental (social, built and natural) and policy.[37] It further organizes physical activity around four active living domains: household, occupational, transport and recreational[36] which were used to report the data. Again, an adapted OSOP approach[35] was used to compare men's/women's and urban/rural accounts. The findings presented below are illustrated by extracts with anonymised participant identifiers that include gender, age range (30-39, 40-49, 50-59, 60-69), setting (urban/rural) and any self-reported health conditions at the time of the current study.

## RESULTS

As Table 1 shows, 50% (16/32) of participants were aged 50 and above. On average, urban participants were slightly younger (43.1 years, SD=12.3) than their rural counterparts (51.6 years, SD=16.8), and length of stay was much shorter in the urban (17.9 years, SD=7.2) than the rural (36.2 years, SD=17.9) setting. Overall, 53% (17/32) had not been educated beyond primary school, but urban participants were better educated than rural participants (73%, 11/15 of urban participants had secondary school or higher education compared with only 24%, 4/17 of rural participants). Most urban participants (80%, 12/15) were either self-employed or salaried, whereas almost all rural participants (94%, 16/17) were involved in subsistence activities. Over half (66%, 21/32) were married, 25% (8/32, all women) reported being divorced/widowed and 9% (3/32, all men) had never married. The participants had all been identified as having pre-diabetes during the 2013-16 NCD survey; some (9%, 3/32: one urban woman, one rural woman and one rural man)



For peer review only - http://bmjopen.bmj.com/site/about/guidelines.xhtml

reported progression to diabetes, and 34.4% (11/32: two of whom also reported diabetes) reported a subsequent hypertension diagnosis.

**Table 1 Focus group participant characteristics**

| | All (N=32) (%) | Urban (N=15) (46.9%) | | | Rural (N=17) (53.1%) | | |
|---|---|---|---|---|---|---|---|
| | | Total | Female | Male | Total | Female | Male |
| **Sex** | | | | | | | |
| Male | 14(43.8) | 6(40.0) | - | 6(100) | 8(47.1) | - | 8(100) |
| Female | 18(56.2) | 9(60.0) | 9(100) | - | 9(52.9) | 9(100) | - |
| **Age** | | | | | | | |
| Age (years)[a] | 47.6(15.3) | 43.1(12.3) | 46.7(11.9) | 37.7(11.7) | 51.6(16.8) | 54.4(10.9) | 48.5(22.1) |
| ≤35 | 8(25.0) | 5(33.3) | 3(33.3) | 2(33.3) | 3(17.7) | 0(0.0) | 3(37.5) |
| 36-49 | 8(25.0) | 4(26.7) | 2(22.2) | 2(33.3) | 4(23.5) | 3(33.3) | 1(12.5) |
| ≥50 | 16(50.0) | 6(40.0) | 4(44.5) | 2(33.3) | 10(58.8) | 6(66.7) | 4(50.0) |
| **Length of stay** | | | | | | | |
| Length of stay (years)[a] | 27.7(16.6) | 17.9(7.2) | 20.1(8.4) | 14.7(3.0) | 36.2(17.9) | 29.8(12.6) | 43.5(21.1) |
| 6-10 years | 3(9.4) | 2(13.3) | 2(22.2) | 0(0.0) | 1(5.9) | 1(11.1) | 0(0.0) |
| 11-15 years | 5(15.6) | 5(33.3) | 1(11.1) | 4(66.7) | 0(0.0) | 0(0.0) | 0(0.0) |
| 16-20 years | 5(15.6) | 3(20.0) | 1(11.1) | 2(33.3) | 2(11.8) | 2(22.2) | 0(0.0) |
| ≥ 20 years | 19(59.4) | 5(33.3) | 5(55.6) | 0(0.0) | 14(82.3) | 8(88.9) | 6(75.0) |
| **Highest education** | | | | | | | |
| Primary school | 17(53.1) | 4(26.7) | 4(44.4) | 0(0.0) | 13(76.5) | 8(88.9) | 5(62.5) |
| Secondary and higher | 15(46.9) | 11(73.3) | 5(55.6) | 6(100.0) | 4(23.5) | 1(11.1) | 3(37.5) |
| **Employment** | | | | | | | |
| Not working | 2(6.3) | 2(13.3) | 2(22.2) | 0(0.0) | 0(0.0) | 0(0.0) | 0(0.0) |
| Subsistence | 17(53.1) | 1(6.7) | 1(11.1) | 0(0.0) | 16(94.1) | 8(88.9) | 8(100.0) |
| Self-employed | 8(25.0) | 8(53.3) | 5(55.6) | 3(50.0) | 0(0.0) | 0(0.0) | 0(0.0) |
| Salaried | 5(15.6) | 4(26.7) | 1(11.1) | 3(50.0) | 1(5.9) | 1(11.1) | 0(0.0) |
| **Marital Status** | | | | | | | |
| Never married | 3(9.4) | 1(6.7) | 0(0.0) | 1(16.7) | 2(11.8) | 0(0.0) | 2(25.0) |
| Married | 21(65.6) | 11(73.3) | 6(66.7) | 5(83.3) | 10(58.8) | 4(44.4) | 6(75.0) |
| Widowed/ divorced | 8(25.0) | 3(20.0) | 3(33.3) | 0(0.0) | 5(29.4) | 5(55.6) | 0(0.0) |
| **Reported health condition** | | | | | | | |
| Pre-diabetic only | 20(62.5) | 11(73.3) | 5(55.6) | 6(100.0) | 9(52.9) | 5(55.6) | 4(50.0) |
| Diabetic only | 1(3.1) | 0(0.0) | 0(0.0) | 0(0.0) | 1(5.9) | 1(11.1) | 0(0.0) |
| Hypertensive only | 9(28.1) | 3(20.0) | 3(33.3) | 0(0.0) | 6(35.3) | 3(33.3) | 3(37.5) |
| Diabetic and hypertensive | 2(6.3) | 1(6.7) | 1(11.1) | 0(0.0) | 1(5.9) | 0(0.0) | 1(12.5) |

Data are presented as n (%), or [a] mean (SD)

For peer review only



For peer review only - http://bmjopen.bmj.com/site/about/guidelines.xhtml

The following sections present participants' accounts of physical activity in their daily lives. In particular, they focus on the context in relation to the types of physical activity people described doing and how these have changed over time, and the factors influencing physical activity.

**What do adults in Malawi do when they are physically active?**

Our analysis of the types of physical activity undertaken showed that Malawian adults were active across all four domains of the ecological model.[36] Men and women in both urban and rural settings reported involvement in a range of household, occupational, transport and recreational activities (see also Supplementary materials Table S2). These are now elaborated in turn.

*Household physical activity*

Overall, women reported doing more household physical activity than men. In both urban and rural settings, women appeared to take the lead in housework and preparing meals:

> *As a woman you are supposed to do physical activities which are household chores, we sweep around the surrounding, we prepare breakfast, we mop the house, then we go to the market to buy relish and some other things when you come back, time is already up for lunch, you start preparing lunch…* (Female, 50-59, urban, hypertension reported)

Rural women also described carrying wood, collecting food from their farms for meals and pounding maize or cassava for flour. Likewise, rural men reported undertaking vigorous household activities, including digging and cutting wood:

> *As for me I am physically active in the morning, such as cutting wood, weeding [gardening] at home […] In the afternoon I rest and thereafter around 4 pm I*



For peer review only - http://bmjopen.bmj.com/site/about/guidelines.xhtml

*start again clearing outside the house...* (Male, 70-79, rural, hypertension reported)

By comparison, urban men described less strenuous household activities, such as car washing and maintenance. Some also described involvement in household chores and cooking:

*I sweep the surrounding, I mop, I cook.*

Facilitator: *Do you stay alone?*

*No, I live with my relatives, but I help them* [with] *household chores.* (Male, 40-49, urban, no health conditions reported)

*Occupational physical activity*

For rural participants, most work appeared to involve effortful physical activity. Both women and men reported daily involvement in farming activities, including planting, weeding and harvesting their crops. One woman said:

*A person has a lot of work to do, like in this rainy season we do farming. Instead of sleeping you think of going to the garden to do farming activities […]. In the afternoon if you left some ridges which you didn't plant the seeds, you go back and plant the stems.* (Female, 50-59, rural, no health condition reported)

As this extract indicates, there was some overlap between household and occupational physical activities in the accounts of rural participants. Similarly, many rural men described fishing on Lake Malawi both to provide food for their families and to sell to make money. Like farming, fishing involves vigorous physical activity, including launching and rowing boats and landing catches. In contrast, urban men talked about running shops and market stalls, working in the service industry and having office jobs, which, as one man described, were often largely sedentary:



For peer review only - http://bmjopen.bmj.com/site/about/guidelines.xhtml

*Mostly I do my work while seated working on the computer, if I move around that means I'm just delivering files to my boss' office, when I knock off from work I use a car…* (Male, 50-59, urban, no health condition reported)

Many urban women participants did not work outside the home. The exceptions were two women who had their own businesses. One of these described how running her market stall involved heavy physical work:

*…I sell timber at [local] market, I cut the timber myself I bought an electric saw blade, I have a wheelbarrow I use it to carry sand when the rains fall, I push it myself as part of exercise.* (Female, 40-49, urban, no health condition reported)

*Transport-related physical activity*

Most rural participants reported that walking was still their main mode of getting from place to place. Many described walking to work (their farm or to go fishing), to the market and to visit friends or family. Urban participants also reported walking as part of their daily routines, but to a lesser extent than their rural counterparts. Many preferred to use public transport, taxis (including bicycle taxis) or their own cars instead of walking:

*I cannot go to some places by foot while I have a car. I would prefer to fill my car with fuel than walking, despite that we are making our bodies not to be physically active.* (Male, 50-59, urban, no health condition reported)

Importantly, despite motorised transport often being less available outside towns and cities in Malawi, some rural participants also described avoiding walking where possible:



For peer review only - http://bmjopen.bmj.com/site/about/guidelines.xhtml

*Nowadays it's different from the past, in the past our parents could walk by foot from here to South Africa but currently if I am going to Jetty [less than 5 km] I prefer to use a car.* (Male, 50-59, rural, hypertension reported)

*Recreational physical activity*

Both rural and urban participants reported involvement in a range of recreational physical activities, including traditional dancing (apart from urban men); jogging (apart from rural women), cycling (men only), playing with their children (mainly women, but also mentioned by men) and team sports (e.g., netball for women and football for men). Football was highly popular among both urban and rural men, and its importance as a recreational activity is illustrated by this account from one younger rural man describing how he and his friends often improvise so they can enjoy a game of football after work:

*During rainy season we like playing football in a club but in the summer season we do farming in marsh gardens and after that we play football using a ball made from plastic papers and rubbers.* (Male, 20-29, rural, no health condition reported)

A number of urban (but not rural) participants described going to the gym or attending exercise classes. As one woman highlighted, involvement in formal types of exercising helped to compensate for the largely inactive lifestyles that people living in towns and cities often led:

*… here in town we find the gym to be our solution in making our bodies active while in the villages they work hence making their bodies active....* (Female, 60-69, urban, hypertension reported)

*Decline in physical activity in Malawi over time*



For peer review only - http://bmjopen.bmj.com/site/about/guidelines.xhtml

In general, people living in rural areas reported doing more strenuous physical activity within the home and at work, and also walking more than those living in the city. However, most participants felt that physical activity levels within the general population were decreasing in Malawi. They were also aware that being less active placed them at a disadvantage compared to previous generations:

> *Our parents were walking long distances with heavy luggage on their heads, up to 10 km on foot, even farming they could do by themselves, without employing other people to help them but nowadays we can't manage all these. Our bodies are weak while our parents were strong.* (Female, 30-39, urban, no health condition reported)

**Factors influencing physical activity in Malawian adults**

The seemingly inevitable decline in household, occupational and transport-related physical activity, suggests the promotion of physical activity (including recreational) should become a public health priority in Malawi. In order to inform the development of effective physical activity interventions, the following sections explore factors that support and prevent people in Malawi from being physically active (see also Supplementary materials Table S3).

*Health*

Participants in all focus groups agreed that being physically active was important for their general health and wellbeing. Some participants described how their diagnosis as being prediabetic in the 2013-16 NCD survey had acted as a catalyst for them to become more active:

> *As for me I started to do exercises regularly when I was told that I am pre-diabetic and each time I go to the hospital diabetes [blood sugar] is high[er] - this*



For peer review only - http://bmjopen.bmj.com/site/about/guidelines.xhtml

*motivated me to do more exercises to reduce my BP and blood sugar level.* (Female, 50-59, urban, hypertension reported)

Discussions within all focus groups also raised the role of physical activity in losing weight. One man directly attributed the health improvements he had experienced following his diagnosis to the weight he had lost through being active:

> *Before I was diagnosed with diabetes, I was overweight, but after the physical activities – I do activities such as digging, working in rice farm, and I found that my weight has been reduced but also sugar level has gone down and I was told not to continue testing my sugar. So, I realize that it's because of exercises.* (Male, 60-69, rural, diabetes and hypertension reported)

Others described how being physically active helped with releasing tension and maintaining flexibility. Importantly, as the following extract illustrates, there was general agreement in all focus groups about the need to do physical activity to remain strong:

> *When a person is doing physical exercise, the body gains strength; if the body was weak and doesn't feel good, but after doing exercise the body feels well.* (Female, 60-69, rural, no health conditions reported)
>
> Facilitator: *Is there anything else on the reasons we are told to do physical exercise? […]*
>
> *… if you just stay idle, you don't have strength in your body* (Female, 60-69, rural, hypertension reported)

*Motivations and barriers to being physically active*

In addition to the consensus around health benefits, rural participants described necessity – having to provide and care for their families – as their main motivator for



For peer review only - http://bmjopen.bmj.com/site/about/guidelines.xhtml

being physically active. As one woman explained, livelihood and physical activity are still often inextricably linked in rural communities:

> *The reason we are physically active especially here on earth, it's because we want to eat. Without this, there won't be anything to eat*. (Female, 50-59, rural, diabetes reported)

Family and work responsibilities also featured in the urban focus group discussions, but here their negative impact on physical activity was the dominant discourse. For example, urban men agreed the multiple, competing pressures they experienced in their daily lives left little time room for recreational physical activity. As one participant suggested, making money is the number one priority for many men living in towns and cities:

> *Being physically active is a very good thing and it helps a lot of things in our bodies, but it seems here in town we are busy making money. Taking care of family and feeding our parents make us forget everything about ourselves, we can't have time to do exercises, which is very bad.* (Male, 50-59, urban, no health condition reported)

The move from active to more sedentary occupations and lifestyles, and the pressure to work hard to make money to provide for the family, were therefore major barriers to being physically active among urban participants. Other common barriers that were mentioned in all focus groups included poor diet, age and fear of injury, which as one rural participant explained prevented some older women from taking part in local recreational activities:

> *All these things we do at home because we are aged and we can't participate in netball which the young ladies are doing. We can't play with them because we fear to be pulled down.* (Female, 60-69, rural, hypertension reported)



For peer review only - http://bmjopen.bmj.com/site/about/guidelines.xhtml

In addition, the view that people who were experiencing ill-health should avoid being active was widespread, particularly, as the following extract illustrates, when illness was combined with age:

> *I was leaving everything for my father to do, while I was just staying, but after some time he was diagnosed with high blood pressure and other diseases. So I said to my father that he should not work anymore but I should be the one doing physical work while he should be resting.* (Male, 30-39, rural, no health condition reported)

However, some participants admitted they could not always blame external factors for their inactivity. Many talked about 'laziness' and 'developing laziness' as their only excuse:

> Facilitator: *What do you think would stop you from doing [exercises] regularly?*
>
> *Laziness.* (Female, 50-59, urban, no health conditions reported)
>
> *Same… laziness* (Female, 40-49, urban, no health conditions reported)

Interestingly, this tendency to describe oneself "lazy", which appeared to have negative connotations and to be associated with risk of disease and weakness, was evident across all focus groups, even among rural men and women, where daily physical activity levels were often extremely high.

*Technology*

The negative influence of technological advancements on physical activity emerged as an independent theme in our analysis. With the exception of the increased availability of motorized transport (which is discussed further in the next section), technology mainly seemed to be having an impact in rural areas. Rural men described how new tools and machinery had taken some of the hard work out of farming, and



For peer review only - http://bmjopen.bmj.com/site/about/guidelines.xhtml

rural women reported how being able to take their maize for processing at the local mill, instead of doing it themselves at home, was a symbol of their social standing:

> *…at present I can take 50 kgs of maize and start pounding but people will get surprised of me why I am doing such activity while am having maize mill closer to me, so instead of doing such activity I try hard to get money and take the maize to the maize mill.* (Female, 50-59, rural, diabetes reported)

*Social norms*

The societal value of wealth generation was discussed in all focus groups, and, as the previous extract suggests, having money in Malawi is associated with social expectations that further limit people's opportunities for physical activity. For example, those who can afford it tend to employ other people to do the physical work (e.g., household chores and farming) that they and their family previously had to do for themselves. This trend was particularly evident in the urban discussions:

> *… maybe the only difference [from people living in rural areas] is farming, they use their own hands… we living here in town we will search for piece-work [casual work] men to do that for us and then pay them later, that's why our life seem to be weak.* (Female, 30-39, urban, no health condition reported)

It was also evident that walking from place to place is not something that Malawians who can afford other forms of transport do through choice. One urban man clearly described how as he earned more money, the amount of physical activity he expended in getting to work gradually declined:

> *I was walking to and from [work], then I got a promotion and I started using public transport when going to work, because I had managed to get another promotion I started going to work using company car and now that I have my*



For peer review only - http://bmjopen.bmj.com/site/about/guidelines.xhtml

*own car I use it as well. I have completely forgotten walking, just because I have money and everything and this has contributed to my low level of physical activity.* (Male, 50-59, urban, no health condition reported)

Expectations of what other people would think or say emerged as a strong influence on the amount and types of physical activity people did, particularly among urban participants. One man described how being made fun of because of his weight had put him off cycling:

*I take my bike to the market, people laugh at me sometimes because I am overweight and when they see me riding a bicycle they say I will break the bike… because they're used in seeing me in the car. What people say it can discourage you from doing exercises*. (Male, 20-29, urban, no health condition reported)

Likewise, some urban women described how gender and age-related norms limited the types of physical activity that was appropriate for women to do, and how the predominantly [younger] male clientele in many gyms made them feel uncomfortable:

*In [local gym] women cannot manage it is full of the youths and men.* (Female, 40-49, urban, no health condition reported)

Children were also reported as having a negative influence on people's (mainly women's) physical activity. Some women recounted experiences of children laughing when they engaged in recreational physical activity. Others described strong social expectations that children should help with physical household chores:

*My neighbors asked me why I draw water by myself when I have children, I tell them it's part of my exercises.* (Female, 50-59, urban, no health conditions reported).



For peer review only - http://bmjopen.bmj.com/site/about/guidelines.xhtml

*Social support*

Despite children's negative impact on some aspects of physical activity, many participants (particularly women) spoke about how their children and grandchildren helped them be active by playing sports and other (traditional) games with them:

> *I invite my grandchildren to play Jingle* [traditional physical game] *because if I just sit down I feel like my legs are tied up, so I jump a little bit and my grandchildren also do the same.* (Female, 60-69, rural, no health condition reported)

Similarly, involvement in sports (e.g., football, netball) organized by local community groups, including churches, was seen as beneficial participants in all focus groups. One urban woman said that knowing other people were depending on her was far more motivational than doing physical activity alone:

> *I think doing on our own is what makes us fail to exercise. Whenever we feel tired we stop the routine, but creating groups in our locations can strengthen the desire of doing exercises. If we can just be 15 we can always be meeting, and one may feel bad to be absent a single day because you feel your friends will be missing your part if it is a team.* (Female, 40-49, urban, no health condition reported)

**Suggestions to help Malawian adults increase physical activity**

Our analysis shows that although other people often had a negative influence on people's physical activity, social forms of recreational physical activity were clearly helpful in encouraging people to be active in both urban and rural settings. Other suggestions to promote recreational and other types of physical activity were mainly targeted at an individual level. These included providing more information on the

For peer review only

benefits of physical activity, and making physical activity part of normal routine (in both urban and rural focus groups), promoting good time management (particularly in urban focus groups), and importantly, ensuring that people find activities they enjoy doing.

> *What the heart has liked, it can't be stopped. Whenever you wake up you will do it*. (Female, 40-49, rural, hypertension reported)

## DISCUSSION

This qualitative study demonstrates that men and women previously identified as having pre-diabetes in rural Malawi tend to engage in more vigorous physical activity in their daily lives than their urban counterparts. Recreational physical activities were practiced in both settings, but tended to focus around social interaction and having fun in the rural setting, whilst there was a move towards more structured forms of exercise, including the use of gym facilities, in the urban setting. Involvement in recreational physical activities reflected a recognition of the need to remain active as the amount of physical activity involved in everyday living decreases in Malawi. Indeed, the understanding that physical activity is important for maintaining good health was widespread. However, aging, ill health and fear of injury emerged as perceived individual level barriers to being active across all focus groups, and these were compounded by lack of time due to interpersonal level barriers such as competing family and work responsibilities, wealth accumulation and negative social influences, particularly in the urban setting.

Although quantitative studies have previously demonstrated differences in physical activity between urban and rural areas of Malawi,[9 10] to our knowledge, this is the first in-depth qualitative exploration of the factors underpinning urban/rural and gender differences in physical activity among Malawian adults. It suggests that the urban/rural



For peer review only - http://bmjopen.bmj.com/site/about/guidelines.xhtml

differences in part reflect the nature of work people in rural areas are involved in and necessity, with most rural occupations, such as farming and fishing, and household chores involving high levels of physical effort (although this may be changing with the advent of new technologies). In addition, the growth of motorized transportation in cities, already evident in more developed Sub-Saharan Africa countries like South Africa,[38] now also appears to be negatively impacting on physical activity in urban Malawi. However, it is important to note that social expectations surrounding wealth mean that a preference to use motorized transport rather than walking, where (financially) possible, was also evident among rural participants.

Gender differences in type of physical activity, in particular women doing more household physical activity than men, reflects the fact that the traditional gender divide (with women bearing the main responsibility for domestic and family activities) remains in Malawian society.[39] Interestingly, however, the role of physical activity in maintaining strength as people age was recognized by women as well as men. The importance of doing regular strength-based physical activity has long been recognized in WHO physical activity guidance,[40 41] and has gained prominence in the latest UK physical activity guidelines in an effort to increase uptake.[42] The value of strength based-exercises in maintaining functionality and avoiding frailty,[43] is likely to be more salient (and therefore potentially motivational) in Malawi, where as elsewhere in Sub-Saharan Africa, older people often play a central role in bringing up children and running small holdings, where younger family members have moved to seek work in cities.[44]

Both men and women in the current study reported a desire to lose weight as a motivator for physical activity. This finding contradicts much previous research suggesting that a larger body size is socially desirable among men and women in Malawi and other Sub-Saharan African countries,[45 46] and that a lower body weight is



For peer review only - http://bmjopen.bmj.com/site/about/guidelines.xhtml

often associated with HIV/AIDS and other diseases, stress and poverty.[24 47 48] The recognition that physical activity aids weight management may suggest that in Malawi, as elsewhere in Sub-Saharan Africa,[23 49] Westernization is leading to a shift from traditional weight preferences to a smaller body size. An alternative explanation may be the fact that our participants' exposure to health promotion information through involvement in the 2013-16 NCD survey encouraged our participants to provide views that would be socially acceptable from a public health perspective. Focus group with people with suspected diabetes in Uganda, who may also have been exposed to health messaging, similarly revealed negative views about obesity, whereas focus groups with people without diabetes viewed excess body weight as a sign of success.[24]

Many of the barriers to being physically active reported in our study were similar to those found in previous studies in Sub-Saharan Africa. These include negative social influences,[20-22 26] lack of time, [20 21 23] and fear of injury.[20] In South Africa, issues around personal safety and crime emerged as major barriers to physical activity, particularly for women.[21 22] However, our participants presented no such concerns. This may reflect the fact Malawi currently experiences lower crime rates than most other Sub-Saharan African countries,[50] but also that our participants were older and perhaps felt less at risk than the younger participants in the other studies may have done.[51-53]

Importantly, almost all participants recognized the need to improve physical activity in Malawi. Table 2 summarizes potential strategies to address barriers at different levels of the ecological model[36 37] for consideration in future interventions. The strategies reflect the barriers and opportunities identified by participants and previous evidence on the effectiveness of embedding physical activity within daily routines,[54] highlighting



For peer review only - http://bmjopen.bmj.com/site/about/guidelines.xhtml

**Table 2 Strategies to improve physical activity levels in Malawi mapped against individual, interpersonal, environment/organisational and policy level barriers**

| Ecological model level | Barrier/opportunity | Strategy |
|---|---|---|
| Individual | **Barriers**<br>• Ill health and fear of injury<br>• Age | 1. Information on health benefits of physical activity.[2 55]<br>2. Information on exercising safely, including as people age.[2 54 56] |
| | **Opportunities**<br>• Maintaining health and wellbeing<br>• Losing weight<br>• Maintaining strength | See 1 and 2 |
| | • Willingness to exercise at home<br>• Recognition of importance of enjoyment | 3. Information on easy to do exercises, which can be done alone without support. [54 56]<br>4. Promotion of a range of activities (including offering taster sessions) to allow people to find something they enjoy doing.[2 57] |
| Interpersonal | **Barriers**<br>• Discouragement (including gender-based) from others<br>• Wealth-related societal expectations<br>• Family responsibilities<br>• Time constraints due to competing priorities | 5. Information campaigns to normalize and promote the value of different forms of physical activity, including for both women and men.[2 55]<br>6. Encourage family involvement in physical activity to motivate each other. [55 58]<br>7. Promoting forms of physical activity that can fit into daily routines i.e., walking.[54 56 57]<br>See also 4 |
| | **Opportunities**<br>• Playing with children<br>• Recognition of the value of group-based activities | 8. Promote the benefits (including bonding) associated with exercising with children.[59]<br>9. Encourage group based physical activity.[54 56 57]<br>See also 4, 6 and 8 |
| Environment/ organizational | **Barriers**<br>• Urban gyms dominated by [young] men<br>• Fewer formal physical activity facilities in rural areas | 10. Set aside female only and other inclusive time slots in gyms.[56]<br>11. Invest in recreational infrastructure such as parks and gyms accessible by both men and women in rural, as well as urban, areas. [2 56 60]<br>12. Encourage people (including those in urban areas) to exercise outdoors using footpaths and parks.[60]<br>See also 5 |
| | **Opportunities**<br>• Churches, schools, community organizations | 13. Encourage church, school and community facilities to promote (social) physical activities.[2 56] |
| | • People engage in sedentary activities at workplaces | 14. Workplace initiatives to improve physical activity (e.g., walking breaks; gym membership) with incentives for participation.[2 54 55 57 60 61]<br>15. Encourage people to park a walking distance from workplace.[54] |
| Policy | **Barriers**<br>• Growth in use of private cars | 16. Improve quality and accessibility of public transport systems. [56]<br>17. Improve availability of footpaths and invest in cycling and other active transport infrastructure. [2 55-57 60 62] |

For peer review only



For peer review only - http://bmjopen.bmj.com/site/about/guidelines.xhtml

the personal benefits/value of physical activity,[2 55] group based exercising,[58] and promoting workplace based physical activity.[60 61 63]

As Malawi continues to develop economically,[64 65] it is likely that (in part due to the societal expectations associated with personal wealth) requirements for occupational, transport and household physical activity will reduce,[66] meaning that recreational physical activity will become increasingly important to help people maintain healthy levels of physical activity.[60] The current study revealed that people in Malawi are already involved in a range of recreational physical activities. However, urban residents described engaging more in structured, facility-dependent exercising (although there was evidence that some groups felt excluded from gyms), whilst physical activity facilities were not mentioned by rural participants. These findings suggest that work needs to be done in ensuring equality of opportunity in relation to physical activity in Malawi. This would include both investment in accessible physical activity facilities in rural areas, but also the promotion of less formal activities, such as dancing, walking and being active with family members,[21 67-70] in urban areas, to ensure that everyone can find a form of physical activity they enjoy.

This study had a number of strengths. First, its design (separate focus groups with men and women in urban and rural settings) allowed systematic comparison of the similarities and differences in men and women's experiences of, and attitudes towards, physical activity in urban and rural Malawi. In addition, the team approach to identifying and defining the analytic themes enhanced the robustness (validity) of our results.[71] However, it was not without limitations. Importantly, the participants had been told they were at high risk of developing diabetes following their participation in previous health research. All had been advised to attend their local health clinic or hospital, where they may have received information about the



For peer review only - http://bmjopen.bmj.com/site/about/guidelines.xhtml

importance of physical activity for their health. They were also older and some were dealing with other health conditions, so may have been more likely than the general adult population of Malawi to try to include different forms of physical activity in their daily lives. In addition, whilst focus groups were chosen to support the development of rich accounts of physical activity by enabling participants to build on each other's accounts, there is potential that they may also adjust their own responses to conform to what they perceive as the views of other group members, thus limiting the richness of the data collected.[72]

## CONCLUSION

This study provides detailed insights into the physical activity views and practices of men and women in urban and rural Malawi, which are important to inform future interventions to increase physical activity. The reported decline in routine physical activity is likely to continue as Malawi, like other countries in the Sub-Saharan Africa region, undergoes social and economic transition, highlighting the need to promote recreational forms of physical activity. The findings suggest that in addition to information campaigns to raise awareness of the benefits of physical activity for all ages, the importance of finding forms of physical activity that people enjoy and the value of (and opportunities for) being physically active with other people, including children and grandchildren, should also be promoted. Finally, public investment in recreational facilities in rural and urban areas that are fully accessible for women, as well as men, and in active travel infrastructure may also be required to ensure that healthy levels of physical activity continue to be part of everyday life in Malawi.

## Acknowledgments

The authors would like to thank: Hazel Namadingo for her input while employed by MEIRU in designing the topic guide, conducting the focus groups in Lilongwe and

For peer review only



For peer review only - http://bmjopen.bmj.com/site/about/guidelines.xhtml

quality assuring all transcripts; Cecelia Nyirenda for conducting the focus groups and undertaking the transcription and translation in Karonga; and Maisha Nyasulu for the transcription and translation in Lilongwe.

**Contributors**

CMG, CB, JMG and AC designed and conducted the study and acquired the data. JB, CMG and CB conceived and designed the analyses. JB and CMG interpreted the data and contributed to drafting the manuscript. All authors contributed to revising the manuscript critically for important intellectual content and all authors approved the version of the manuscript to be published.

**Funding**

This study was funded by the Medical Research Council (MR/R019428/1). The funder had no influence on the analysis plan, results presented or decision to publish.

**Competing interests**

None declared.

**Participant consent for publication**

Not required.

**Ethics approval**

The study was approved by the National Health Sciences Research Committee in Malawi (18/01/1951) and University of Glasgow College of Medical, Veterinary and Life Sciences Ethics Committee (200170173) in the UK. All participants provided written informed consent prior to their participation in the focus groups.

**Data availability statement**

Data are available on request from the corresponding author.

For peer review only



For peer review only - http://bmjopen.bmj.com/site/about/guidelines.xhtml

## REFERENCES

1. World Health Organization. Noncommunicable Diseases Progress Monitor 2020: World Health Organization, 2020.

2. World Health Organization. Physical Activity Factsheet. 2018. Available: https://www.who.int/en/news-room/fact-sheets/detail/physical-activity. [accessed January 2020].

3. Darling AM, Sunguya B, Ismail A, et al. Gender differences in nutritional status, diet and physical activity among adolescents in eight countries in sub-Saharan Africa. *Trop Med Int Health* 2020;**25**(1):33-43.

4. Delisle H, Ntandou-Bouzitou G, Agueh V, et al. Urbanisation, nutrition transition and cardiometabolic risk: the Benin study. *Br J Nutr* 2012;**107**(10):1534-44.

5. Kirunda BE, Wamani H, Fadnes LT, et al. Objectively Assessed Physical Activity and Associated Factors Among Adults in Peri-Urban and Rural Eastern Uganda: A Population-based Study. *J Phys Act Health* 2016;**13**(11):1243-54.

6. Barr AL, Young EH, Sandhu MS. Objective measurement of physical activity: improving the evidence base to address non-communicable diseases in Africa. *BMJ Glob Health* 2018;**3**(5):e001044.

7. World Health Organization. Noncommunicable diseases country profiles 2011: World Health Organization, 2011.

8. World Health Organization. Noncommunicable diseases country profiles 2018: World Health Organization, 2018.

9. Divala OH, Amberbir A, Ismail Z, et al. The burden of hypertension, diabetes mellitus, and cardiovascular risk factors among adult Malawians in HIV care: consequences for integrated services. *BMC Public Health* 2016;**16**(1):1243-43.

For peer review only

10. Msyamboza KP, Ngwira B, Dzowela T, et al. The burden of selected chronic non-communicable diseases and their risk factors in Malawi: nationwide STEPS survey. *PLoS One* 2011;**6**(5):e20316.

11. World Health Organization. Insufficient physical activity: prevalence of insufficient physical activity among adults. 2018. Available: http://gamapserver.who.int/gho/interactive_charts/ncd/risk_factors/physical_inactivity/atlas.html?indicator=i1&date=Male. [accessed March 2020].

12. Price AJ, Crampin AC, Amberbir A, et al. Prevalence of obesity, hypertension, and diabetes, and cascade of care in sub-Saharan Africa: a cross-sectional, population-based study in rural and urban Malawi. *Lancet Diabetes Endocrinol* 2018;**6**(3):208-22.

13. Soares ALG, Banda L, Amberbir A, et al. Sex and area differences in the association between adiposity and lipid profile in Malawi. *BMJ Glob Health* 2019;**4**(5):e001542-e42.

14. Bigna JJ, Noubiap JJ. The rising burden of non-communicable diseases in sub-Saharan Africa. *Lancet Glob Health* 2019;**7**(10):e1295-e96.

15. Gowshall M, Taylor-Robinson SD. The increasing prevalence of non-communicable diseases in low-middle income countries: the view from Malawi. *Int J Gen Med* 2018;**11**:255-64.

16. World Health Organization. Projections of mortality and causes of death, 2016 to 2060. 2018. Available: https://www.who.int/healthinfo/global_burden_disease/projections/en/. [accessed January 2020].

17. Anderson E, Durstine JL. Physical activity, exercise, and chronic diseases: A brief review. *Sports Med Health Sci* 2019;**1**(1):3-10.



For peer review only - http://bmjopen.bmj.com/site/about/guidelines.xhtml

18. Warburton DER, Nicol CW, Bredin SSD. Health benefits of physical activity: the evidence. *Can Med Assoc J* 2006;**174**(6):801.

19. Milton K, Macniven R, Bauman A. Review of the epidemiological evidence for physical activity and health from low- and middle-income countries. *Glob Public Health* 2014;**9**(4):369-81.

20. Tuakli-Wosornu YA, Rowan M, Gittelsohn J. Perceptions of physical activity, activity preferences and health among a group of adult women in urban Ghana: a pilot study. *Ghana Med J* 2014;**48**(1):3-13.

21. Watson ED, Norris SA, Draper CE, et al. "Just because you're pregnant, doesn't mean you're sick!" A qualitative study of beliefs regarding physical activity in black South African women. *BMC Pregnancy Childbirth* 2016;**16**(1):174-74.

22. Ware LJ, Prioreschi A, Bosire E, et al. Environmental, Social, and Structural Constraints for Health Behavior: Perceptions of Young Urban Black Women During the Preconception Period-A Healthy Life Trajectories Initiative. *J Nutr Educ Behav* 2019;**51**(8):946-57.

23. Phillips EA, Comeau DL, Pisa PT, et al. Perceptions of diet, physical activity, and obesity-related health among black daughter-mother pairs in Soweto, South Africa: a qualitative study. *BMC Public Health* 2016;**16**(1):750.

24. Mayega RW, Etajak S, Rutebemberwa E, et al. 'Change means sacrificing a good life': perceptions about severity of type 2 diabetes and preventive lifestyles among people afflicted or at high risk of type 2 diabetes in Iganga Uganda. *BMC Public Health* 2014;**14**(1):864.

25. Ogunrinu T, Gamboa-Maldonado T, Ngewa RN, et al. A qualitative study of health education experiences and self-management practices among patients with type 2



For peer review only - http://bmjopen.bmj.com/site/about/guidelines.xhtml

diabetes at Malamulo Adventist Hospital in Thyolo District, Malawi. *Malawi Med J* 2017;**29**(2):118-23.

26. Mphwanthe G, Carolan M, Earnesty D, et al. Perceived barriers and facilitators to diet and physical activity among adults diagnosed with type 2 diabetes in Malawi. *Glob Public Health* 2021;**16**(3):469-84.

27. Malambo P, Kengne AP, Lambert EV, et al. Prevalence and socio-demographic correlates of physical activity levels among South African adults in Cape Town and Mount Frere communities in 2008-2009. *Arch Public Health* 2016;**74**:54-54.

28. Guthold R, Louazani SA, Riley LM, et al. Physical activity in 22 African countries: results from the World Health Organization STEPwise approach to chronic disease risk factor surveillance. *Am J Prev Med* 2011;**41**(1):52-60.

29. Hoare E, Stavreski B, Jennings GL, et al. Exploring Motivation and Barriers to Physical Activity among Active and Inactive Australian Adults. *Sports (Basel)* 2017;**5**(3):47.

30. Chaabane S, Chaabna K, Abraham A, et al. Physical activity and sedentary behaviour in the Middle East and North Africa: An overview of systematic reviews and meta-analysis. *Sci Rep* 2020;**10**(1):9363.

31. Leung FH, Savithiri R. Spotlight on focus groups. *Canadian family physician Medecin de famille canadien* 2009;**55**(2):218-9.

32. Morgan DL. Paradigms Lost and Pragmatism Regained: Methodological Implications of Combining Qualitative and Quantitative Methods. *Journal of Mixed Methods Research* 2007;**1**(1):48-76.

33. Braun V, Clarke V. Successful Qualitative Research: a practical guide for beginners. London: SAGE, 2013.

For peer review only



For peer review only - http://bmjopen.bmj.com/site/about/guidelines.xhtml

34. Braun V, Clarke V. Using thematic analysis in psychology. *Qual Res Psychol* 2006;**3**:77-101.

35. Ziebland S, McPherson A. Making sense of qualitative data analysis: an introduction with illustrations from DIPEx (personal experiences of health and illness). *Medical education* 2006;**40**(5):405-14.

36. Sallis JF, Owen N, Fisher EB. Ecological Models of Health Behaviours. In: Glanz K, Rimer BK, Viswanath K, eds. Health Behaviour and Health Education: Theory, Research and Practice. 4th ed. San Francisco: Jossey-Bass, 2008:465-85.

37. Bauman AE, Reis RS, Sallis JF, et al. Correlates of physical activity: why are some people physically active and others not? *Lancet* 2012;**380**(9838):258-71.

38. Luke R. Car ownership perceptions and intentions amongst South African students. *J Transp Geogr* 2018;**66**:135-43.

39. National Statistical Office, ICF. Malawi Demographic and Health Survey 2015-16. Zomba, Malawi, and Rockville, Maryland, USA: NSO and ICF, 2017.

40. World Health Organization. Global Recommendations on Physical Activity for Health. Geneva: World Health Organization, 2010.

41. World Health Organization. WHO guidelines on physical activity and sedentary behaviour. Geneva: World Health Organisation, 2020.

42. Department of Health and Social Care. UK Chief Medical Officers' Physical Activity Guidelines. UK: Department of Health and Social Care, 2019:66.

43. Hillsdon M, Foster C. What are the health benefits of muscle and bone strengthening and balance activities across life stages and specific health outcomes? *J Frailty Sarcopenia Falls* 2018;**3**(2):66-73.

44. Aboderin IAG, Beard JR. Older people's health in sub-Saharan Africa. *Lancet* 2015;**385**(9968):e9-e11.



For peer review only - http://bmjopen.bmj.com/site/about/guidelines.xhtml

45. Flax VL, Thakwalakwa C, Phuka JC, et al. Body size preferences and food choice among mothers and children in Malawi. *Matern Child Nutr* 2020;**16**(4):e13024.

46. Okoro EO, Oyejola BA, Etebu EN, et al. Body size preference among Yoruba in three Nigerian communities. *Eat Weight Disord* 2014;**19**(1):77-88.

47. Matoti-Mvalo T, Puoane T. Perceptions of body size and its association with HIV/AIDS. *South Afr J Clin Nutr* 2011;**24**(1):40-45.

48. Puoane T, Tsolekile L, Steyn N. Perceptions about body image and sizes among Black African girls living in Cape Town. *Ethn Dis* 2010;**20**(1):29-34.

49. Benkeser RM, Biritwum R, Hill AG. Prevalence of overweight and obesity and perception of healthy and desirable body size in urban, Ghanaian women. *Ghana Med J* 2012;**46**(2):66-75.

50. Institute for Economics and Peace. Global Peace Index 2020: Measuring Peace in a Complex World. Sydney, 2020.

51. Kayira J. Malawi: Rape, defilement cases horrify a nation. 2020. Available: https://face2faceafrica.com/article/malawi-rape-defilement-cases-horrify-a-nation. [accessed August 2021].

52. Masiye M. Defilement, Rape Up to 43% in Eastern Region. 2020. Available: https://zodiakmalawi.com/nw/national-news/66-news-in-southern-region/2893-defilement-rape-up-to-43-in-eastern-region. [accessed August 2021].

53. Sapuli D. Malawi has over 2100 child rapists in its prisons. 2020. Available: https://malawi24.com/2020/10/30/malawi-has-over-2100-child-rapists-in-its-prisons/. [accessed August 2021].

54. Centers for Disease Control and Prevention. Overcoming Barriers to Physical Activity. 2020. Available: https://www.cdc.gov/physicalactivity/basics/adding-pa/barriers.html. [accessed September 2020].

For peer review only



For peer review only - http://bmjopen.bmj.com/site/about/guidelines.xhtml

55. Tuso P. Strategies to Increase Physical Activity. *Perm J* 2015;**19**(4):84-88.

56. Heath GW, Parra DC, Sarmiento OL, et al. Evidence-based intervention in physical activity: lessons from around the world. *Lancet* 2012;**380**(9838):272-81.

57. Ferguson-Stegall L, Robb JD. Effective Strategies to Increase Physical Activity in the Working Years. *ACSMs Health Fit J* 2019;**23**(5).

58. Brandt CJ, Clemensen J, Nielsen JB, et al. Drivers for successful long-term lifestyle change, the role of e-health: a qualitative interview study. *BMJ Open* 2018;**8**(3):e017466.

59. Paquette D, Dumont C. Is father–child rough-and-tumble play associated with attachment or activation relationships? *Early Child Development and Care* 2013;**183**(6):760-73.

60. Guthold R, Stevens GA, Riley LM, et al. Worldwide trends in insufficient physical activity from 2001 to 2016: a pooled analysis of 358 population-based surveys with 1.9 million participants. *Lancet Glob Health* 2018;**6**(10):e1077-e86.

61. Centers for Disease Control and Prevention. Worksite Physical Activity. September 28, 2017 2017. Available: https://www.cdc.gov/physicalactivity/worksite-pa/index.htm. [accessed September 2020].

62. Brown DR, Heath GW, Martin SL, editors. *Promoting Physical Activity: A Guide for Community Action*. 2 ed. Champaign, IL: Human Kinetics, 2010.

63. World Health Organization. Global action plan on physical activity 2018–2030: more active people for a healthier world: World Health Organization, 2018.

64. National Planning Commission. Malawi's vision: An inclusively wealthy and self-reliant nation. Lilongwe, Malawi: National Planning Commission, 2020.



For peer review only - http://bmjopen.bmj.com/site/about/guidelines.xhtml

65. Government of Malawi. Malawi Growth and Development Strategy III: Building a Productive, Competitive and Resilient Nation. Lilongwe, Malawi: Government of Malawi, 2017.

66. Ng SW, Popkin BM. Time use and physical activity: a shift away from movement across the globe. *Obes Rev* 2012;**13**(8):659-80.

67. Chaabane S, Chaabna K, Doraiswamy S, et al. Barriers and Facilitators Associated with Physical Activity in the Middle East and North Africa Region: A Systematic Overview. *J Environ Res Public Health* 2021;**18**(4).

68. Harrison AL, Taylor NF, Shields N, et al. Attitudes, barriers and enablers to physical activity in pregnant women: a systematic review. *J Physiother* 2018;**64**(1):24-32.

69. Saligheh M, McNamara B, Rooney R. Perceived barriers and enablers of physical activity in postpartum women: a qualitative approach. *BMC Pregnancy Childbirth* 2016;**16**(1):131.

70. Beune EJAJ, Haafkens JA, Agyemang C, et al. Inhibitors and enablers of physical activity in multiethnic hypertensive patients: qualitative study. *J Hum Hypertens* 2010;**24**(4):280-90.

71. Morse JM. Critical Analysis of Strategies for Determining Rigor in Qualitative Inquiry. *Qualitative health research* 2015;**25**(9):1212-22.

72. Carey MA, Smith MW. Capturing the Group Effect in Focus Groups: A Special Concern in Analysis. *Qualitative health research* 1994;**4**(1):123-27.



For peer review only - http://bmjopen.bmj.com/site/about/guidelines.xhtml

## Supplementary materials

## Physical activity focus group topic guide

1. Tell me about when you are physically active/do exercise in your daily life. What sort of things do you do? Where? Why? (Prompt for work, travel and leisure activity)
2. How would you say your activity levels compare with your parents/grandparents? Why is this? (Prompt changes to work, leisure and travel – moving to different place)
3. Have your own activity levels changed over the years? Why is this?
4. What are the benefits to you of being physically active? (Prompt: fitness, strength, flexibility) how important are these to you/other people in your community? Why is this?
5. What do you understand by exercise?
6. Do you ever do exercise? What do you do and where? Who do you do it with? What motivates you to do exercise?
7. Tell me about any social/community occasions which involve physical activity/exercise?
8. What do you know about physical activity and health? ([Prompt: Benefits and risks of being physically active, including in relation to diabetes) How do you know this? (Prompt: where did this knowledge come from?)
9. How could you do more physical activity in your daily life? What might stop you doing this?

Demonstrate and lead an exercise session including resistance exercises. Ask the participants what they think of them as they do them.

10. Afterwards, ask: which of these exercises might you do again (if any)? Why have you chosen these? What would help you do these regularly? What would stop you doing them regularly?
11. Which of the exercises would you not like to do again? Why is this?

For peer review only

## Table S1: Detailed description and example extracts from the nine broad themes reported

| Theme | Description | Example extracts |
|---|---|---|
| *Context* | | |
| 1. Types of physical activity undertaken | Activities participants reported doing that made them physically active or inactive | *The physical work which I usually do is, I like going to the lake and launch the fish nets. When I come back home, I make sure that outside the house is clean, but if it's not clean I tell the children to sweep the ground. Sometimes when we are in rainy season, I wake up early in the morning going to the farm garden, I do weeding – a little bit in maize garden because I don't do for a long time. Usually my work is fishing, I got in the morning going to the lake and arrange the fishing nets and go into the lake.* (Male, 76, rural, hypertension reported)<br><br>*In the morning I take my bath then I prepare to go to work, and mostly I do my work while seated working on the computer, if I move around that means I'm just delivering file to my boss's office, when I knock off from work I use a car off I go home.* (Male, 50, urban, no health conditions reported) |
| 2. Changes in physical activity over time | How physical activity has changed from generation to generation. | *in the past people were digging manually by using a hoe while nowadays we are using animal plough whenever we want to cultivate any kind of crop. The other thing is, they* [past generations] *were carrying things on their head while nowadays we carry everything in an oxcart or in a vehicle … they like building structures while nowadays we like modern structures by hiring other people to do for us while we are sitting down. …* (Male, 25, rural, no health conditions reported) |
| *Influences* | | |
| 3. Physical activity and health | How participants perceive that being physically active influences people's health | *If I stay idle, diabetes disease will affect me because there is nothing I am doing. Any kind of disease can affect you if you are just staying idle but if I am physically active, I might be free from diseases and be strong*. (Female, 60, rural, hypertension reported) |
| 4. Motivations | Participants' reasons for undertaking different physical activities | *The reason we are physically active especially here on earth, it's because we want to eat. Without this, there won't be anything to eat*. (Female, 56, rural, diabetes reported) |
| 5. Barriers to physical activity | Factors hindering involvement in physical activity | *It's what we said that sometimes its laziness because you can plan to do the exercise, but you may end up doing other things*. (Female, 60, rural, hypertension reported)<br><br>*[the] gym is too far from where I live, I live [in one place] and gym is in [another place], mostly I fail to go to gym, and when I* |

| Theme | Description | Example extracts |
|---|---|---|
| | | *go to gym am supposed to pay a certain fee which makes me just give up on gym, but if I had all the needed weights at home I could have been doing exercises at home*. (Male, 38, urban, no health condition reported) |
| 6. Technology and physical activity | How technological advancements affect physical activity | *Technology is pushing us to be…. the same with those who live in rural areas…. let's talk of phones, even maize mills are everywhere nowadays, they no longer use mortar and pestle*. (Female, 34, urban, no health condition reported) |
| 7. Social norms | How other people's views/commonly held opinions and expectations influence physical activity | *… My neighbor's asked me why I draw water by myself when I have children, I tell them it's part of my exercises*. (Female, 54, urban, no health conditions reported)<br>*There are some other exercises that women cannot manage to do like stretching your legs you cannot manage that…* (Female, 58, urban, hypertension reported) |
| 8. Social support | People and social institutions providing support for physical activities | *At our church we have our own team as [name of church] women we play against the youths for practices, but when we have a match, we play against another [name of church] church*. (Female, 59, urban, hypertension reported) |
| *Solutions* | | |
| 9. Ways of improving physical activity | Suggested ways of improving physical activity | *I think a person should join more sports groups, working can be one of them and cycling can be one of them; you can also join groups which can motivate you to be moving or running and make you sweat.* (Male, 25, rural, no health conditions reported) |

For peer review only

**Table S2 Types of physical activity described by participants within the household, occupational, transport and recreational domains of the ecological model**

| Household* | Who | | | | Occupational* | Who | | | | Transport | Who | | | | Recreational | Who | | | |
|---|---|---|---|---|---|---|---|---|---|---|---|---|---|---|---|---|---|---|---|
| | wr | wu | mr | mu | | wr | wu | mr | mu | | wr | wu | mr | mu | | wr | wu | mr | mu |
| Chores (sweeping, mopping) | x | x | - | x | Farming (planting, weeding, harvesting) | x | - | x | - | Walking | x | x | x | x | Dance | x | x | x | - |
| Food shopping | x | x | x | - | Looking after livestock | - | - | x | - | Cycling | - | - | - | x | Team sports | x | x | x | x |
| Collecting farm produce for meals | x | - | - | - | Fishing on lake | - | - | x | - | Public transport | x | - | x | x | Playing with children | x | x | x | x |
| Cooking | x | x | - | x | Running shops/ market stalls | - | x | - | x | (Bicycle) taxi | - | - | x | x | Jogging | - | x | x | x |
| Food processing (pounding maize) | x | - | - | - | Service industry | - | - | - | x | Personal car | - | x | x | x | Cycling | - | - | x | x |
| Washing clothes | x | x | - | - | Office job | - | - | - | x | Transport goods (by car/ox cart) | - | x | x | - | Gym/exercise classes | - | x | - | x |
| Fetching water | x | x | - | - | | | | | | | | | | | Doing exercises at home | x | x | - | x |
| Cutting wood | - | - | x | - | | | | | | | | | | | | | | | |
| Carrying wood | x | - | - | - | | | | | | | | | | | | | | | |
| Gardening | - | x | x | x | | | | | | | | | | | | | | | |
| Moulding bricks | - | - | x | - | | | | | | | | | | | | | | | |
| Digging pit latrines | - | - | x | - | | | | | | | | | | | | | | | |
| Car washing, maintenance | - | - | - | x | | | | | | | | | | | | | | | |
| Feeding livestock | - | x | - | - | | | | | | | | | | | | | | | |

*In the rural setting there appeared to be some overlap between household and occupational physical activities. wr = rural women, wu = urban women, mr = rural men, mu = urban men

For peer review only

**Table S3: Broad themes and sub-themes summarising influences and their impact on participants' physical activity, mapped to ecological model levels and domains**

| Theme | Sub-theme | Who | | | | Ecological Model | | Impact (+ve/-ve) |
|---|---|---|---|---|---|---|---|---|
| | | wr | wu | mr | mu | Level | Domain | |
| 3) Health | Recognition of health/wellbeing benefits | x | x | x | x | Individual | General | +ve |
| 3) Health | Weight loss | x | x | x | x | Individual | General | +ve |
| 3) Health | Recognition of physical strength benefits | x | x | x | x | Individual | General | +ve |
| 4) Motivations | Family responsibilities | x | - | - | - | Interpersonal | Household | +ve |
| 4) Motivations | Work responsibilities | x | - | x | - | Interpersonal | Occupational | +ve |
| 5) Barriers | Work responsibilities | - | x | - | x | Interpersonal | Recreational | -ve |
| 5) Barriers | Family responsibilities | x | x | x | x | Interpersonal | Recreational | -ve |
| 5) Barriers | Lack of time | - | x | - | x | Individual | Recreational | -ve |
| 5) Barriers | Sense of urgency | - | x | - | x | Individual | General | -ve |
| 5) Barriers | Age | x | x | x | x | Individual | General | -ve |
| 5) Barriers | Ill health | x | x | x | x | Individual | General | -ve |
| 5) Barriers | Fear of injury | x | x | x | x | Individual | General | -ve |
| 5) Barriers | Poor diet | x | x | x | x | Individual | General | -ve |
| 5) Barriers | Lack of exercise knowledge | x | - | - | - | Individual | Recreational | -ve |
| 5) Barriers | Laziness | x | x | x | x | Individual | General | -ve |
| 6) Technology | Maize mills | x | x | - | - | Environment | Household | -ve |
| 6) Technology | Farming tools/machinery | - | - | x | - | Environment | Occupational | -ve |
| 7) Social norms | Pressure to make money | - | - | - | x | Interpersonal | Occupational | -ve |
| 7) Social norms | Wealth | x | x | x | x | Interpersonal | Household/Transport | -ve |
| 7) Social norms | Availability of motorised transport | x | x | x | x | Environment | Transport | -ve |
| 7) Social norms | People making fun of you (e.g., cycling) | - | - | - | x | Interpersonal | Transport | -ve |
| 7) Social norms | Gender (e.g., at gym) | - | x | - | - | Interpersonal | Recreational | -ve |
| 7) Social norms | Expectations that children will help | x | x | x | - | Interpersonal | Household | -ve |
| 7) Social norms | Children making fun of you | x | x | - | - | Interpersonal | Recreational | -ve |
| 8) Social support | Playing with children | x | x | x | x | Interpersonal | Recreational | +ve |
| 8) Social support | Team sports/exercising with others | x | x | x | x | Interpersonal | Recreational | +ve |

wr = rural women, wu = urban women, mr = rural men, mu = urban men

For peer review only

# Standards for Reporting Qualitative Research (SRQR)*

http://www.equator-network.org/reporting-guidelines/srqr/

## Title and abstract

| | Page/line no(s). |
|---|---|
| **Title** - Concise description of the nature and topic of the study Identifying the study as qualitative or indicating the approach (e.g., ethnography, grounded theory) or data collection methods (e.g., interview, focus group) is recommended | Page 1, lines 1-2 |
| **Abstract** - Summary of key elements of the study using the abstract format of the intended publication; typically includes background, purpose, methods, results, and conclusions | Page 2-3, lines 20-47 |

## Introduction

| | |
|---|---|
| **Problem formulation** - Description and significance of the problem/phenomenon studied; review of relevant theory and empirical work; problem statement | Pages 5-7, lines 62-116 |
| **Purpose or research question** - Purpose of the study and specific objectives or questions | Page 7, lines 116-122 |

## Methods

| | |
|---|---|
| **Qualitative approach and research paradigm** - Qualitative approach (e.g., ethnography, grounded theory, case study, phenomenology, narrative research) and guiding theory if appropriate; identifying the research paradigm (e.g., postpositivist, constructivist/ interpretivist) is also recommended; rationale** | Page 7 lines 125-133<br>Page 9, lines 159-160 |
| **Researcher characteristics and reflexivity** - Researchers' characteristics that may influence the research, including personal attributes, qualifications/experience, relationship with participants, assumptions, and/or presuppositions; potential or actual interaction between researchers' characteristics and the research questions, approach, methods, results, and/or transferability | Page 9, lines 165-176 |
| **Context** - Setting/site and salient contextual factors; rationale** | Page 8, lines 135-141 |
| **Sampling strategy** - How and why research participants, documents, or events were selected; criteria for deciding when no further sampling was necessary (e.g., sampling saturation); rationale** | Page 8, lines 136-150 |
| **Ethical issues pertaining to human subjects** - Documentation of approval by an appropriate ethics review board and participant consent, or explanation for lack thereof; other confidentiality and data security issues | Pages 8-9, lines 141-142, 158-159; Pages 30-31, lines 638-641 |
| **Data collection methods** - Types of data collected; details of data collection procedures including (as appropriate) start and stop dates of data collection and analysis, iterative process, triangulation of sources/methods, and modification of procedures in response to evolving study findings; rationale** | Pages 7-8, lines 125-135; Page 9 158-171 |
| **Data collection instruments and technologies** - Description of instruments (e.g., interview guides, questionnaires) and devices (e.g., audio recorders) used for data collection; if/how the instrument(s) changed over the course of the study | Supplementary material<br>Page 9, lines 158-171 |
| **Units of study** - Number and relevant characteristics of participants, documents, or events included in the study; level of participation (could be reported in results) | Page 8, lines 136-150; Pages 11-12, lines 218-232; Page 12, Table 1 |



For peer review only - http://bmjopen.bmj.com/site/about/guidelines.xhtml

| | |
|---|---|
| **Data processing** - Methods for processing data prior to and during analysis, including transcription, data entry, data management and security, verification of data integrity, data coding, and anonymization/de-identification of excerpts | Page 9, lines 172-182; Page 11, lines 213-216 |
| **Data analysis** - Process by which inferences, themes, etc., were identified and developed, including the researchers involved in data analysis; usually references a specific paradigm or approach; rationale** | Pages 9-11, lines 178-213 |
| **Techniques to enhance trustworthiness** - Techniques to enhance trustworthiness and credibility of data analysis (e.g., member checking, audit trail, triangulation); rationale** | Pages 9-10, lines 178-201 |

**Results/findings**

| | |
|---|---|
| **Synthesis and interpretation** - Main findings (e.g., interpretations, inferences, and themes); might include development of a theory or model, or integration with prior research or theory | Pages 13-24, lines 238-502; Supplementary materials Tables S2 and S3 |
| **Links to empirical data** - Evidence (e.g., quotes, field notes, text excerpts, photographs) to substantiate analytic findings | Pages 13-24, lines 238-502; Supplementary materials, Table S1 |

**Discussion**

| | |
|---|---|
| **Integration with prior work, implications, transferability, and contribution(s) to the field -** Short summary of main findings; explanation of how findings and conclusions connect to, support, elaborate on, or challenge conclusions of earlier scholarship; discussion of scope of application/generalizability; identification of unique contribution(s) to scholarship in a discipline or field | Pages 24-28, lines 504-587 |
| **Limitations** - Trustworthiness and limitations of findings | Page 3, lines 51-58 Pages 28-29, lines 588-603 |

**Other**

| | |
|---|---|
| **Conflicts of interest** - Potential sources of influence or perceived influence on study conduct and conclusions; how these were managed | Page 30, line 634 |
| **Funding** - Sources of funding and other support; role of funders in data collection, interpretation, and reporting | Page 30, lines 631-632 |

*The authors created the SRQR by searching the literature to identify guidelines, reporting standards, and critical appraisal criteria for qualitative research; reviewing the reference lists of retrieved sources; and contacting experts to gain feedback. The SRQR aims to improve the transparency of all aspects of qualitative research by providing clear standards for reporting qualitative research.

**The rationale should briefly discuss the justification for choosing that theory, approach, method, or technique rather than other options available, the assumptions and limitations implicit in those choices, and how those choices influence study conclusions and transferability. As appropriate, the rationale for several items might be discussed together.

**Reference:**

O'Brien BC, Harris IB, Beckman TJ, Reed DA, Cook DA. **Standards for reporting qualitative research: a synthesis of recommendations.** *Academic Medicine*, Vol. 89, No. 9 / Sept 2014
DOI: 10.1097/ACM.0000000000000388



For peer review only - http://bmjopen.bmj.com/site/about/guidelines.xhtml

BMJ Open

# A qualitative study of practices and attitudes towards physical activity among pre-diabetic men and women in urban and rural Malawi

| | |
|---|---|
| Journal: | *BMJ Open* |
| Manuscript ID | bmjopen-2021-058261.R2 |
| Article Type: | Original research |
| Date Submitted by the Author: | 03-Nov-2022 |
| Complete List of Authors: | Banda, Jethro; Malawi Epidemiology and Intervention Research Unit<br>Bunn, Christopher; University of Glasgow, School of Social and Political Sciences, Institute of Health and Wellbeing; Malawi Epidemiology and Intervention Research Unit<br>Crampin, Amelia; London School of Hygiene and Tropical Medicine; Malawi Epidemiology and Intervention Research Unit<br>Gill, Jason; University of Glasgow, Institute of Cardiovascular and Medical Sciences<br>Gray, Cindy; University of Glasgow, School of Social and Political Sciences, Institute of Health and Wellbeing |
| <b>Primary Subject Heading</b>: | Public health |
| Secondary Subject Heading: | Global health, Qualitative research |
| Keywords: | General diabetes < DIABETES & ENDOCRINOLOGY, PUBLIC HEALTH, QUALITATIVE RESEARCH |
| | |

SCHOLARONE™
Manuscripts

*I, the Submitting Author has the right to grant and does grant on behalf of all authors of the Work (as defined in the below author licence), an exclusive licence and/or a non-exclusive licence for contributions from authors who are: i) UK Crown employees; ii) where BMJ has agreed a CC-BY licence shall apply, and/or iii) in accordance with the terms applicable for US Federal Government officers or employees acting as part of their official duties; on a worldwide, perpetual, irrevocable, royalty-free basis to BMJ Publishing Group Ltd ("BMJ") its licensees and where the relevant Journal is co-owned by BMJ to the co-owners of the Journal, to publish the Work in this journal and any other BMJ products and to exploit all rights, as set out in our licence.*

*The Submitting Author accepts and understands that any supply made under these terms is made by BMJ to the Submitting Author unless you are acting as an employee on behalf of your employer or a postgraduate student of an affiliated institution which is paying any applicable article publishing charge ("APC") for Open Access articles. Where the Submitting Author wishes to make the Work available on an Open Access basis (and intends to pay the relevant APC), the terms of reuse of such Open Access shall be governed by a Creative Commons licence – details of these licences and which Creative Commons licence will apply to this Work are set out in our licence referred to above.*

*Other than as permitted in any relevant BMJ Author's Self Archiving Policies, I confirm this Work has not been accepted for publication elsewhere, is not being considered for publication elsewhere and does not duplicate material already published. I confirm all authors consent to publication of this Work and authorise the granting of this licence.*

For peer review only

# A qualitative study of practices and attitudes towards physical activity among pre-diabetic men and women in urban and rural Malawi

Mr Jethro Banda, Malawi Epidemiology and Intervention Research Unit, Lilongwe, Malawi. Jethro.Banda@meiru.mw

Dr Christopher Bunn PhD, School of Social and Political Sciences, Institute of Health and Wellbeing, University of Glasgow, UK and Malawi Epidemiology and Intervention Research Unit, Lilongwe, Malawi. Christopher.Bunn@glasgow.ac.uk

Professor Amelia Crampin MBChB DTM&H MSc FFPHM, Malawi Epidemiology and Intervention Research Unit, Lilongwe, Malawi, Institute of Health and Wellbeing, University of Glasgow, UK and London School of Hygiene and Tropical Medicine, London, UK. Mia.Crampin@lshtm.ac.uk

Professor Jason MR Gill PhD, Institute of Cardiovascular and Medical Sciences, University of Glasgow, UK. Jason.Gill@glasgow.ac.uk

Professor Cindy M Gray* PhD, School of Social and Political Sciences, Institute of Health and Wellbeing, University of Glasgow, UK. cindy.gray@glasgow.ac.uk

*Corresponding author

For peer review only

## ABSTRACT

**Objectives** Given the decline in physical activity levels in Malawi, like other Sub-Saharan African countries, and its implication for non-communicable disease (NCD) prevention, this study aimed to compare and contrast accounts of practices and attitudes towards physical activity among Malawian men and women (previously identified as having pre-diabetes) in urban and rural settings.

**Setting** Two communities: one urban (Lilongwe) and one rural (Karonga)

**Participants** 14 men (urban N=6, rural N=8) and 18 women (urban N=9, rural N=9) classified as pre-diabetic during their participation in an NCD survey 3-5 years previously.

**Design** A qualitative focus group study (N=4) and thematic analysis, with the ecological model used as a framework to characterize the types of physical activity people engaged in and the factors influencing them.

**Results** Participants reported undertaking different types of physical activity across all ecological model domains (household, occupational, transport, recreational). Rural participants reported more vigorous physical activities than urban participants, and women reported more household activities than men. Many participants recognized a need to promote physical activity in Malawi, and the health benefits of doing so, including the importance of physical activity in helping them stay strong to maintain physical functioning. Barriers to physical activity included competing priorities (especially urban men), societal expectations around wealth, use of motorized transport, lack of accessible facilities for women, aging and ill health.

**Conclusions** Physical activity is declining in Malawi as working and transport practices change in response to economic development, making promotion of


For peer review only

alternative forms of physical activity a public health priority. Multi-level interventions emphasizing the personal benefits/value of physical activity for all ages, and routine and group-based exercising, as well as investment in accessible recreational facilities (including for women) and active travel infrastructure should be considered to improve physical activity levels in Malawi.

**Keywords** Physical activity, facilitators, barriers, interventions, ecological model, qualitative, Sub-Saharan Africa, Malawi.

**Strengths and limitations of this study**

- Conducting separate focus groups with men and women in urban and rural settings allowed systematic comparison of urban/rural and gender differences in physical activity.
- Participants had previously been told they were at high risk of developing diabetes and may been more informed about the importance of physical activity for their health than the general adult population of Malawi.
- Whilst focus groups enabled participants to build on each other's accounts, no examination of the extent to which the group context influenced individual responses (e.g. conformity) was undertaken.

For peer review only

## INTRODUCTION

Lack of physical activity is thought to be the fourth leading risk factor for premature mortality globally.[1] The World Health Organization (WHO) recommends that adults undertake 150 minutes of moderate-intensity or 75 minutes of vigorous-intensity physical activity a week for optimal health.[2] However, a quarter of adults fail to meet these guidelines worldwide.[2] In Sub-Saharan Africa, physical activity levels have historically been high, but increased urbanization and adoption of Westernized lifestyles mean physical inactivity is increasing.[3-6] In Malawi, the most recent WHO country profiles published in 2011 and 2018 demonstrate that the prevalence of physical inactivity rose from 10% to 14% in the eight years to 2016.[7,8] In addition, some studies have reported lower physical activity levels in Malawi's urban areas,[9] with one population survey reporting that 24% of urban residents were inactive compared with 9% of rural residents.[10] Gender differences are less clear: while population surveys report that men in Malawi are generally more physically active than women;[10 11] some studies in specific urban and rural settings show that women are more active than men.[12 13]

The prevalence of non-communicable diseases (NCD), such as diabetes, cardiovascular disease, chronic respiratory disease and cancer, is on the rise in Sub-Saharan Africa.[14 15] Deaths from NCD in low and middle income countries are forecast to increase from 31.5 million in 2016 to 41.9 million in 2030 and to 56.6 million by 2045.[16] Recent estimates suggest that 32% of deaths in Malawi are associated with NCD.[1] Although being physically active helps both prevention and management of NCD,[17-19] currently Malawi has no physical activity public health campaigns.[1]

Promotion of behaviour change for physical activity requires detailed understanding of current local practices and attitudes, as well as the different contexts in which physical



For peer review only - http://bmjopen.bmj.com/site/about/guidelines.xhtml

activity is undertaken. However, only a handful of qualitative studies exploring practices and attitudes towards physical activity in Sub-Saharan Africa have been conducted. Interviews and focus group discussions with urban women in Ghana revealed a desire for more social (including group-based) physical activity, and that lack of time and concerns about injury (e.g., doing age-appropriate exercises) were other barriers to being physically active.[20] Three studies with women in urban South Africa[21-23] showed that although participants were aware of the physical and mental health benefits of physical activity (including weight loss); lack of time, money and information, perceptions of local crime, and negative influences from other people, including abuse from men for wearing "exercise" clothing, were barriers to their being more physically active. Focus groups with men and women with suspected type 2 diabetes, suspected pre-diabetes or who were obese in urban and rural Uganda suggested that people were willing to become more active to improve their health, and some described doing this by embedding physical activity within their normal routines.[24] This included washing, cooking, walking and cycling in urban areas, and more vigorous activities such as gardening, bush clearing, fetching water, splitting firewood and pounding food in rural areas. However, any gender differences were not explored.

Two qualitative studies on physical activity have been conducted in Malawi in men and women with diabetes. In one study, interviews and focus groups with diabetes patients attending health education classes at a rural private hospital revealed that people were aware of the importance of both routine and leisure (recreational) physical activity for their health; however, no gender comparisons were reported.[25] In a separate study, focus group discussions with patients in urban and semi-urban areas reported both positive and negative social influences. Specifically, while ridicule from other people



For peer review only - http://bmjopen.bmj.com/site/about/guidelines.xhtml

hindered their participation in some types of physical activity, encouragement from health workers and family members (including taking part in household activities) helped them remain active.[26]

Despite declining physical activity levels[6 15] and the implication of this for NCD prevention and management,[17-19] there is currently very little evidence about the practices and attitudes of adults in Malawi towards physical activity. As physical activity (and its determinants) may differ according to gender and place of residence,[2 27-30] the aim of this qualitative study was to compare and contrast accounts of practices and attitudes towards physical activity among Malawian men and women in urban and rural settings who had previously been identified as having pre-diabetes. This new evidence will be useful in informing the development of effective physical activity interventions in Malawi.

## METHODS

### Study design

To elicit accounts of physical activity in everyday life among urban and rural Malawians living with pre-diabetes, we used a focus group-based research design. Focus groups were chosen to enable participants to develop rich accounts of physical activity in their communities in an interactive setting that encouraged them to elaborate and examine the narratives offered by fellow participants.[31] This approach was framed within a pragmatist philosophy,[32] which positions participants' narratives as neither objects of knowledge which enable prediction nor as the 'truth' of those the participants. Rather, the narratives are seen as offering insight into the 'vocabularies' of physical activity that are used by participants and which can be engaged with when seeking change.

### Setting and recruitment



For peer review only - http://bmjopen.bmj.com/site/about/guidelines.xhtml

Data collection took place in Lilongwe (urban) and Karonga (rural) in March 2019. Participants were recruited from a sample of adults previously identified (during participation in an NCD survey between 2013 and 2016)[12] as having pre-diabetes. All were part of a larger MRC-funded study (MR/R019428/1; NHSRC protocol 18/01/1951) investigating links between nutritional intake and physical, metabolic and inflammatory phenotypes in pre-diabetic (n=50) and normoglycaemic (n=50) men and women. During the informed consent process for this larger study, participants were asked if they would be willing to participate in a focus group if selected. No refusals were recorded during this process, and participants from the 'pre-diabetic' sub-group were purposively sampled to achieve variation in age.

**Participants**

Four focus groups were held with men and women separately in the urban and rural locations. They included a total of 32 participants (urban N=15, 6 men, 9 women; rural N=17, 8 men, 9 women). At the time of the current study, some participants reported progression from their pre-diabetic status at the last NCD survey to diabetes and/or hypertension.

**Participant and public involvement**

At the start of the MRC-funded study, stakeholder engagement meetings with community leaders, community members, healthcare professionals, policy makers and patient group were held in both rural and urban locations to raise awareness of the research and gather feedback on the design and implementation of data collection procedures, including the focus group discussions reported here.

**Data collection and management**



For peer review only - http://bmjopen.bmj.com/site/about/guidelines.xhtml

The focus groups lasted between 90 and 120 minutes and were audio recorded with participant consent. A semi-structured topic guide (see Supplementary materials) was developed informed by our pragmatist approach[32] to focus discussion around participants' personal accounts of physical activity in their daily lives, their understandings of physical activity and health (including the benefits of being physically active), perceived barriers and facilitators to being physically active (both personal and among others in their community) and their perceptions of changes in physical activity over time. The topic guide was developed in English by CB, CMG and Hazel Namadingo (HN), all experienced qualitative researchers, then translated into Chichewa for urban focus groups and into Chitumbuka for rural focus groups by fluent Chichewa and Chitumbuka speakers. The urban focus groups were conducted by HN; the rural focus groups were conducted by Cecelia Nyirenda, an experienced field worker. The facilitators were fluent in the local languages and familiar with the issues under discussion.

The audio recordings, together with field notes taken during the focus groups, were transcribed directly into English by experienced field workers fluent in Chichewa and Chitumbuka. HN (native Chichewa speaker) and JB (native Chichewa speaker and fluent in Chitumbuka) then quality assured the transcripts while listening to the audio recordings, and made changes where necessary.

For peer review only

### Data analysis

The quality-assured transcripts were analyzed using a thematic codebook approach.[33] JB, CMG and CB read through the transcripts line by line to familiarize themselves with the data and identify initial codes relating to participants' accounts, knowledge, barriers and facilitators of physical activity, and any emerging areas of interest (such as changes in physical activity over time in Malawi). The three authors then met to



For peer review only - http://bmjopen.bmj.com/site/about/guidelines.xhtml

discuss and combine their initial codes into broad semantic themes to reflect participants' accounts of physical activity. JB applied the broad themes to all transcripts using NVivo11 software to organize the data, and an iterative approach to identify all data within each theme and consider whether the themes adequately reflected the meanings represented in the data or needed refined (e.g., by coding additional potentially relevant extracts for inclusion in the analysis as 'Other'). The resultant coded transcripts were reviewed by CMG, any disagreements resolved through discussion and all extracts coded as 'Other' jointly examined to decide whether they represented a new broad theme, belonged to an existing theme or could be discarded. All were either assigned to an existing theme or discarded. JB then further refined the broad themes into sub-themes to produce a detailed narrative account of each broad theme, which included careful reading, re-reading and detailed notetaking using an adapted one sheet of paper (OSOP) approach[34] to compare similarities and differences in accounts between men and women in urban and rural settings, and to capture interactions within the focus groups. The ecological model of physical activity[35 36] was then applied as a framework to characterize the different types of physical activity participants engaged in. Again, an adapted OSOP approach[34] was used to compare men's/women's and urban/rural accounts. The findings presented below are illustrated by extracts with anonymised participant identifiers that include gender, age range (30-39, 40-49, 50-59, 60-69), setting (urban/rural) and any self-reported health conditions at the time of the current study.

## RESULTS

As Table 1 shows, 50% (16/32) of participants were aged 50 and above. On average, urban participants were slightly younger (43.1 years, SD=12.3) than their rural



For peer review only - http://bmjopen.bmj.com/site/about/guidelines.xhtml

counterparts (51.6 years, SD=16.8), and length of stay was much shorter in the urban (17.9 years, SD=7.2) than the rural (36.2 years, SD=17.9) setting. Overall, 53% (17/32) had not been educated beyond primary school, but urban participants were better educated than rural participants (73%, 11/15 of urban participants had secondary school or higher education compared with only 24%, 4/17 of rural participants). Most urban participants (80%, 12/15) were either self-employed or salaried, whereas almost all rural participants (94%, 16/17) were involved in subsistence activities. Over half (66%, 21/32) were married, 25% (8/32, all women) reported being divorced/widowed and 9% (3/32, all men) had never married. The participants had all been identified as having pre-diabetes during the 2013-16 NCD survey; some (9%, 3/32: one urban woman, one rural woman and one rural man) reported progression to diabetes, and 34.4% (11/32: two of whom also reported diabetes) reported a subsequent hypertension diagnosis.

**Table 1 Focus group participant characteristics**

| | All (N=32) (%) | Urban (N=15) (46.9%) | | | Rural (N=17) (53.1%) | | |
|---|---|---|---|---|---|---|---|
| | | Total | Female | Male | Total | Female | Male |
| **Sex** | | | | | | | |
| Male | 14(43.8) | 6(40.0) | - | 6(100) | 8(47.1) | - | 8(100) |
| Female | 18(56.2) | 9(60.0) | 9(100) | - | 9(52.9) | 9(100) | - |
| **Age** | | | | | | | |
| Age (years)[a] | 47.6(15.3) | 43.1(12.3) | 46.7(11.9) | 37.7(11.7) | 51.6(16.8) | 54.4(10.9) | 48.5(22.1) |
| ≤35 | 8(25.0) | 5(33.3) | 3(33.3) | 2(33.3) | 3(17.7) | 0(0.0) | 3(37.5) |
| 36-49 | 8(25.0) | 4(26.7) | 2(22.2) | 2(33.3) | 4(23.5) | 3(33.3) | 1(12.5) |
| ≥50 | 16(50.0) | 6(40.0) | 4(44.5) | 2(33.3) | 10(58.8) | 6(66.7) | 4(50.0) |
| **Length of stay** | | | | | | | |
| Length of stay (years)[a] | 27.7(16.6) | 17.9(7.2) | 20.1(8.4) | 14.7(3.0) | 36.2(17.9) | 29.8(12.6) | 43.5(21.1) |
| 6-10 years | 3(9.4) | 2(13.3) | 2(22.2) | 0(0.0) | 1(5.9) | 1(11.1) | 0(0.0) |
| 11-15 years | 5(15.6) | 5(33.3) | 1(11.1) | 4(66.7) | 0(0.0) | 0(0.0) | 0(0.0) |
| 16-20 years | 5(15.6) | 3(20.0) | 1(11.1) | 2(33.3) | 2(11.8) | 2(22.2) | 0(0.0) |
| ≥ 20 years | 19(59.4) | 5(33.3) | 5(55.6) | 0(0.0) | 14(82.3) | 8(88.9) | 6(75.0) |
| **Highest education** | | | | | | | |
| Primary school | 17(53.1) | 4(26.7) | 4(44.4) | 0(0.0) | 13(76.5) | 8(88.9) | 5(62.5) |



For peer review only - http://bmjopen.bmj.com/site/about/guidelines.xhtml

| | | | | | | | |
|---|---|---|---|---|---|---|---|
| Secondary and higher | 15(46.9) | 11(73.3) | 5(55.6) | 6(100.0) | 4(23.5) | 1(11.1) | 3(37.5) |
| **Employment** | | | | | | | |
| Not working | 2(6.3) | 2(13.3) | 2(22.2) | 0(0.0) | 0(0.0) | 0(0.0) | 0(0.0) |
| Subsistence | 17(53.1) | 1(6.7) | 1(11.1) | 0(0.0) | 16(94.1) | 8(88.9) | 8(100.0) |
| Self-employed | 8(25.0) | 8(53.3) | 5(55.6) | 3(50.0) | 0(0.0) | 0(0.0) | 0(0.0) |
| Salaried | 5(15.6) | 4(26.7) | 1(11.1) | 3(50.0) | 1(5.9) | 1(11.1) | 0(0.0) |
| **Marital Status** | | | | | | | |
| Never married | 3(9.4) | 1(6.7) | 0(0.0) | 1(16.7) | 2(11.8) | 0(0.0) | 2(25.0) |
| Married | 21(65.6) | 11(73.3) | 6(66.7) | 5(83.3) | 10(58.8) | 4(44.4) | 6(75.0) |
| Widowed/ divorced | 8(25.0) | 3(20.0) | 3(33.3) | 0(0.0) | 5(29.4) | 5(55.6) | 0(0.0) |
| **Reported health condition** | | | | | | | |
| Pre-diabetic only | 20(62.5) | 11(73.3) | 5(55.6) | 6(100.0) | 9(52.9) | 5(55.6) | 4(50.0) |
| Diabetic only | 1(3.1) | 0(0.0) | 0(0.0) | 0(0.0) | 1(5.9) | 1(11.1) | 0(0.0) |
| Hypertensive only | 9(28.1) | 3(20.0) | 3(33.3) | 0(0.0) | 6(35.3) | 3(33.3) | 3(37.5) |
| Diabetic and hypertensive | 2(6.3) | 1(6.7) | 1(11.1) | 0(0.0) | 1(5.9) | 0(0.0) | 1(12.5) |

Data are presented as n (%), or [a] mean (SD)

Our analysis identified 10 broad themes reflecting participants' accounts of physical activity in their daily lives. Two of these described the context of physical activity in Malawi – 1) Types of physical activity undertaken; 2) Changes in physical activity over time. Six described factors that participants perceived as influencing physical activity – 3) Physical activity and health; 4) Motivations for physical activity; 5) Barriers to physical activity; 6) Technology and physical activity; 7) Social norms; 8) Social support. One described potential solutions – 9) Ways of improving physical activity. (A final theme – 10) Sources of information, is not elaborated in this paper). A detailed description of the nine themes reported here, together with example extracts, is provided in Supplementary materials (Table S1). The following sections present participants' accounts of physical activity in their daily lives. In particular, they focus on the context in relation to the types of physical activity people described doing and how these have changed over time, the factors influencing physical activity and potential ways to improve physical activity in Malawi.



For peer review only

For peer review only - http://bmjopen.bmj.com/site/about/guidelines.xhtml

### What do adults in Malawi do when they are physically active?

The ecological model constructs physical activity as influenced by interacting factors operating at different levels: individual, interpersonal, environmental (social, built and natural) and policy.[35] It further organizes physical activity around four active living domains: household, occupational, transport and recreational[36]. Our analysis of the types of physical activity undertaken showed that Malawian men and women in both urban and rural settings were active across all four ecological model domains (see also Supplementary materials Table S2). These are now elaborated in turn.

*Household physical activity*

Overall, women reported doing more household physical activity than men. In both urban and rural settings, women appeared to take the lead in housework and preparing meals:

> *As a woman you are supposed to do physical activities which are household chores, we sweep around the surrounding, we prepare breakfast, we mop the house, then we go to the market to buy relish and some other things when you come back, time is already up for lunch, you start preparing lunch…* (Female, 50-59, urban, hypertension reported)

Rural women also described carrying wood, collecting food from their farms for meals and pounding maize or cassava for flour. Likewise, rural men reported undertaking vigorous household activities, including digging and cutting wood:

> *As for me I am physically active in the morning, such as cutting wood, weeding [gardening] at home […] In the afternoon I rest and thereafter around 4 pm I start again clearing outside the house...* (Male, 70-79, rural, hypertension reported)



For peer review only - http://bmjopen.bmj.com/site/about/guidelines.xhtml

By comparison, urban men described less strenuous household activities, such as car washing and maintenance. Some also described involvement in household chores and cooking:

> *I sweep the surrounding, I mop, I cook.*
>
> Facilitator: *Do you stay alone?*
>
> *No, I live with my relatives, but I help them* [with] *household chores.* (Male, 40-49, urban, no health conditions reported)

*Occupational physical activity*

For rural participants, most work appeared to involve effortful physical activity. Both women and men reported daily involvement in farming activities, including planting, weeding and harvesting their crops. One woman said:

> *A person has a lot of work to do, like in this rainy season we do farming. Instead of sleeping you think of going to the garden to do farming activities […]. In the afternoon if you left some ridges which you didn't plant the seeds, you go back and plant the stems.* (Female, 50-59, rural, no health condition reported)

As this extract indicates, there was some overlap between household and occupational physical activities in the accounts of rural participants. Similarly, many rural men described fishing on Lake Malawi both to provide food for their families and to sell to make money. Like farming, fishing involves vigorous physical activity, including launching and rowing boats and landing catches. In contrast, urban men talked about running shops and market stalls, working in the service industry and having office jobs, which, as one man described, were often largely sedentary:



For peer review only - http://bmjopen.bmj.com/site/about/guidelines.xhtml

*Mostly I do my work while seated working on the computer, if I move around that means I'm just delivering files to my boss' office, when I knock off from work I use a car…* (Male, 50-59, urban, no health condition reported)

Many urban women participants did not work outside the home. The exceptions were two women who had their own businesses. One of these described how running her market stall involved heavy physical work:

*…I sell timber at [local] market, I cut the timber myself I bought an electric saw blade, I have a wheelbarrow I use it to carry sand when the rains fall, I push it myself as part of exercise.* (Female, 40-49, urban, no health condition reported)

*Transport-related physical activity*

Most rural participants reported that walking was still their main mode of getting from place to place. Many described walking to work (their farm or to go fishing), to the market and to visit friends or family. Urban participants also reported walking as part of their daily routines, but to a lesser extent than their rural counterparts. Many preferred to use public transport, taxis (including bicycle taxis) or their own cars instead of walking:

*I cannot go to some places by foot while I have a car. I would prefer to fill my car with fuel than walking, despite that we are making our bodies not to be physically active.* (Male, 50-59, urban, no health condition reported)

Importantly, despite motorised transport often being less available outside towns and cities in Malawi, some rural participants also described avoiding walking where possible:



For peer review only - http://bmjopen.bmj.com/site/about/guidelines.xhtml

306 *Nowadays it's different from the past, in the past our parents could walk by foot*
307 *from here to South Africa but currently if I am going to Jetty [less than 5 km] I*
308 *prefer to use a car.* (Male, 50-59, rural, hypertension reported)

309 *Recreational physical activity*

310 Both rural and urban participants reported involvement in a range of recreational
311 physical activities, including traditional dancing (apart from urban men); jogging (apart
312 from rural women), cycling (men only), playing with their children (mainly women, but
313 also mentioned by men) and team sports (e.g., netball for women and football for men).
314 Football was highly popular among both urban and rural men, and its importance as a
315 recreational activity is illustrated by this account from one younger rural man
316 describing how he and his friends often improvise so they can enjoy a game of football
317 after work:

318 *During rainy season we like playing football in a club but in the summer season*
319 *we do farming in marsh gardens and after that we play football using a ball*
320 *made from plastic papers and rubbers.* (Male, 20-29, rural, no health condition
321 reported)

322 A number of urban (but not rural) participants described going to the gym or attending
323 exercise classes. As one woman highlighted, involvement in formal types of exercising
324 helped to compensate for the largely inactive lifestyles that people living in towns and
325 cities often led:

326 *… here in town we find the gym to be our solution in making our bodies active*
327 *while in the villages they work hence making their bodies active....* (Female,
328 60-69, urban, hypertension reported)

329 *Decline in physical activity in Malawi over time*



For peer review only - http://bmjopen.bmj.com/site/about/guidelines.xhtml

In general, people living in rural areas reported doing more strenuous physical activity within the home and at work, and also walking more than those living in the city. However, most participants felt that physical activity levels within the general population were decreasing in Malawi. They were also aware that being less active placed them at a disadvantage compared to previous generations:

> *Our parents were walking long distances with heavy luggage on their heads, up to 10 km on foot, even farming they could do by themselves, without employing other people to help them but nowadays we can't manage all these. Our bodies are weak while our parents were strong.* (Female, 30-39, urban, no health condition reported)

For peer review only

**Factors influencing physical activity in Malawian adults**

The seemingly inevitable decline in household, occupational and transport-related physical activity, suggests the promotion of physical activity (including recreational) should become a public health priority in Malawi. In order to inform the development of effective physical activity interventions, the following sections explore factors that support and prevent people in Malawi from being physically active (see also Supplementary materials Table S3).

*Health*

Participants in all focus groups agreed that being physically active was important for their general health and wellbeing. Some participants described how their diagnosis as being prediabetic in the 2013-16 NCD survey had acted as a catalyst for them to become more active:

> *As for me I started to do exercises regularly when I was told that I am pre-diabetic and each time I go to the hospital diabetes [blood sugar] is high[er] - this*



For peer review only - http://bmjopen.bmj.com/site/about/guidelines.xhtml

*motivated me to do more exercises to reduce my BP and blood sugar level.* (Female, 50-59, urban, hypertension reported)

Discussions within all focus groups also raised the role of physical activity in losing weight. One man directly attributed the health improvements he had experienced following his diagnosis to the weight he had lost through being active:

> *Before I was diagnosed with diabetes, I was overweight, but after the physical activities – I do activities such as digging, working in rice farm, and I found that my weight has been reduced but also sugar level has gone down and I was told not to continue testing my sugar. So, I realize that it's because of exercises.* (Male, 60-69, rural, diabetes and hypertension reported)

Others described how being physically active helped with releasing tension and maintaining flexibility. Importantly, as the following extract illustrates, there was general agreement in all focus groups about the need to do physical activity to remain strong:

> *When a person is doing physical exercise, the body gains strength; if the body was weak and doesn't feel good, but after doing exercise the body feels well.* (Female, 60-69, rural, no health conditions reported)

> Facilitator: *Is there anything else on the reasons we are told to do physical exercise? […]*

> *… if you just stay idle, you don't have strength in your body* (Female, 60-69, rural, hypertension reported)

*Motivations and barriers to being physically active*

In addition to the consensus around health benefits, rural participants described necessity – having to provide and care for their families – as their main motivator for



For peer review only - http://bmjopen.bmj.com/site/about/guidelines.xhtml

being physically active. As one woman explained, livelihood and physical activity are still often inextricably linked in rural communities:

> *The reason we are physically active especially here on earth, it's because we want to eat. Without this, there won't be anything to eat*. (Female, 50-59, rural, diabetes reported)

Family and work responsibilities also featured in the urban focus group discussions, but here their negative impact on physical activity was the dominant discourse. For example, urban men agreed the multiple, competing pressures they experienced in their daily lives left little time room for recreational physical activity. As one participant suggested, making money is the number one priority for many men living in towns and cities:

> *Being physically active is a very good thing and it helps a lot of things in our bodies, but it seems here in town we are busy making money. Taking care of family and feeding our parents make us forget everything about ourselves, we can't have time to do exercises, which is very bad.* (Male, 50-59, urban, no health condition reported)

The move from active to more sedentary occupations and lifestyles, and the pressure to work hard to make money to provide for the family, were therefore major barriers to being physically active among urban participants. Other common barriers that were mentioned in all focus groups included poor diet, age and fear of injury, which as one rural participant explained prevented some older women from taking part in local recreational activities:

> *All these things we do at home because we are aged and we can't participate in netball which the young ladies are doing. We can't play with them because we fear to be pulled down.* (Female, 60-69, rural, hypertension reported)



For peer review only - http://bmjopen.bmj.com/site/about/guidelines.xhtml

In addition, the view that people who were experiencing ill-health should avoid being active was widespread, particularly, as the following extract illustrates, when illness was combined with age:

> *I was leaving everything for my father to do, while I was just staying, but after some time he was diagnosed with high blood pressure and other diseases. So I said to my father that he should not work anymore but I should be the one doing physical work while he should be resting.* (Male, 30-39, rural, no health condition reported)

However, some participants admitted they could not always blame external factors for their inactivity. Many talked about 'laziness' and 'developing laziness' as their only excuse:

> Facilitator: *What do you think would stop you from doing [exercises] regularly?*
>
> *Laziness.* (Female, 50-59, urban, no health conditions reported)
>
> *Same… laziness* (Female, 40-49, urban, no health conditions reported)

Interestingly, this tendency to describe oneself "lazy", which appeared to have negative connotations and to be associated with risk of disease and weakness, was evident across all focus groups, even among rural men and women, where daily physical activity levels were often extremely high.

*Technology*

The negative influence of technological advancements on physical activity emerged as an independent theme in our analysis. With the exception of the increased availability of motorized transport (which is discussed further in the next section), technology mainly seemed to be having an impact in rural areas. Rural men described how new tools and machinery had taken some of the hard work out of farming, and



For peer review only - http://bmjopen.bmj.com/site/about/guidelines.xhtml

rural women reported how being able to take their maize for processing at the local mill, instead of doing it themselves at home, was a symbol of their social standing:

> *…at present I can take 50 kgs of maize and start pounding but people will get surprised of me why I am doing such activity while am having maize mill closer to me, so instead of doing such activity I try hard to get money and take the maize to the maize mill.* (Female, 50-59, rural, diabetes reported)

*Social norms*

The societal value of wealth generation was discussed in all focus groups, and, as the previous extract suggests, having money in Malawi is associated with social expectations that further limit people's opportunities for physical activity. For example, those who can afford it tend to employ other people to do the physical work (e.g., household chores and farming) that they and their family previously had to do for themselves. This trend was particularly evident in the urban discussions:

> *… maybe the only difference [from people living in rural areas] is farming, they use their own hands… we living here in town we will search for piece-work [casual work] men to do that for us and then pay them later, that's why our life seem to be weak.* (Female, 30-39, urban, no health condition reported)

It was also evident that walking from place to place is not something that Malawians who can afford other forms of transport do through choice. One urban man clearly described how as he earned more money, the amount of physical activity he expended in getting to work gradually declined:

> *I was walking to and from [work], then I got a promotion and I started using public transport when going to work, because I had managed to get another promotion I started going to work using company car and now that I have my*

For peer review only

*own car I use it as well. I have completely forgotten walking, just because I have money and everything and this has contributed to my low level of physical activity.* (Male, 50-59, urban, no health condition reported)

Expectations of what other people would think or say emerged as a strong influence on the amount and types of physical activity people did, particularly among urban participants. One man described how being made fun of because of his weight had put him off cycling:

*I take my bike to the market, people laugh at me sometimes because I am overweight and when they see me riding a bicycle they say I will break the bike… because they're used in seeing me in the car. What people say it can discourage you from doing exercises*. (Male, 20-29, urban, no health condition reported)

Likewise, some urban women described how gender and age-related norms limited the types of physical activity that was appropriate for women to do, and how the predominantly [younger] male clientele in many gyms made them feel uncomfortable:

*In [local gym] women cannot manage it is full of the youths and men.* (Female, 40-49, urban, no health condition reported)

Children were also reported as having a negative influence on people's (mainly women's) physical activity. Some women recounted experiences of children laughing when they engaged in recreational physical activity. Others described strong social expectations that children should help with physical household chores:

*My neighbors asked me why I draw water by myself when I have children, I tell them it's part of my exercises.* (Female, 50-59, urban, no health conditions reported).



For peer review only - http://bmjopen.bmj.com/site/about/guidelines.xhtml

*Social support*

Despite children's negative impact on some aspects of physical activity, many participants (particularly women) spoke about how their children and grandchildren helped them be active by playing sports and other (traditional) games with them:

> *I invite my grandchildren to play Jingle* [traditional physical game] *because if I just sit down I feel like my legs are tied up, so I jump a little bit and my grandchildren also do the same.* (Female, 60-69, rural, no health condition reported)

Similarly, involvement in sports (e.g., football, netball) organized by local community groups, including churches, was seen as beneficial participants in all focus groups. One urban woman said that knowing other people were depending on her was far more motivational than doing physical activity alone:

> *I think doing on our own is what makes us fail to exercise. Whenever we feel tired we stop the routine, but creating groups in our locations can strengthen the desire of doing exercises. If we can just be 15 we can always be meeting, and one may feel bad to be absent a single day because you feel your friends will be missing your part if it is a team.* (Female, 40-49, urban, no health condition reported)

**Suggestions to help Malawian adults increase physical activity**

Our analysis shows that although other people often had a negative influence on people's physical activity, social forms of recreational physical activity were clearly helpful in encouraging people to be active in both urban and rural settings. Other suggestions to promote recreational and other types of physical activity were mainly targeted at an individual level. These included providing more information on the



For peer review only - http://bmjopen.bmj.com/site/about/guidelines.xhtml

benefits of physical activity, and making physical activity part of normal routine (in both urban and rural focus groups), promoting good time management (particularly in urban focus groups), and importantly, ensuring that people find activities they enjoy doing.

> *What the heart has liked, it can't be stopped. Whenever you wake up you will do it*. (Female, 40-49, rural, hypertension reported)

## DISCUSSION

This qualitative study demonstrates that men and women previously identified as having pre-diabetes in rural Malawi tend to engage in more vigorous physical activity in their daily lives than their urban counterparts. Recreational physical activities were practiced in both settings, but tended to focus around social interaction and having fun in the rural setting, whilst there was a move towards more structured forms of exercise, including the use of gym facilities, in the urban setting. Involvement in recreational physical activities reflected a recognition of the need to remain active as the amount of physical activity involved in everyday living decreases in Malawi. Indeed, the understanding that physical activity is important for maintaining good health was widespread. However, aging, ill health and fear of injury emerged as perceived individual level barriers to being active across all focus groups, and these were compounded by lack of time due to interpersonal level barriers such as competing family and work responsibilities, wealth accumulation and negative social influences, particularly in the urban setting.

Although quantitative studies have previously demonstrated differences in physical activity between urban and rural areas of Malawi,[9 10] to our knowledge, this is the first in-depth qualitative exploration of the factors underpinning urban/rural and gender differences in physical activity among Malawian adults. It suggests that the urban/rural



For peer review only - http://bmjopen.bmj.com/site/about/guidelines.xhtml

differences in part reflect the nature of work people in rural areas are involved in and necessity, with most rural occupations, such as farming and fishing, and household chores involving high levels of physical effort (although this may be changing with the advent of new technologies). In addition, the growth of motorized transportation in cities, already evident in more developed Sub-Saharan Africa countries like South Africa,[37] now also appears to be negatively impacting on physical activity in urban Malawi. However, it is important to note that social expectations surrounding wealth mean that a preference to use motorized transport rather than walking, where (financially) possible, was also evident among rural participants.

Gender differences in type of physical activity, in particular women doing more household physical activity than men, reflects the fact that the traditional gender divide (with women bearing the main responsibility for domestic and family activities) remains in Malawian society.[38] Interestingly, however, the role of physical activity in maintaining strength as people age was recognized by women as well as men. The importance of doing regular strength-based physical activity has long been recognized in WHO physical activity guidance,[39 40] and has gained prominence in the latest UK physical activity guidelines in an effort to increase uptake.[41] The value of strength based-exercises in maintaining functionality and avoiding frailty,[42] is likely to be more salient (and therefore potentially motivational) in Malawi, where as elsewhere in Sub-Saharan Africa, older people often play a central role in bringing up children and running small holdings, where younger family members have moved to seek work in cities.[43]

Both men and women in the current study reported a desire to lose weight as a motivator for physical activity. This finding contradicts much previous research suggesting that a larger body size is socially desirable among men and women in Malawi and other Sub-Saharan African countries,[44 45] and that a lower body weight is



For peer review only - http://bmjopen.bmj.com/site/about/guidelines.xhtml

often associated with HIV/AIDS and other diseases, stress and poverty.[24 46 47] The recognition that physical activity aids weight management may suggest that in Malawi, as elsewhere in Sub-Saharan Africa,[23 48] Westernization is leading to a shift from traditional weight preferences to a smaller body size. An alternative explanation may be the fact that our participants' exposure to health promotion information through involvement in the 2013-16 NCD survey encouraged our participants to provide views that would be socially acceptable from a public health perspective. Focus group with people with suspected diabetes in Uganda, who may also have been exposed to health messaging, similarly revealed negative views about obesity, whereas focus groups with people without diabetes viewed excess body weight as a sign of success.[24]

Many of the barriers to being physically active reported in our study were similar to those found in previous studies in Sub-Saharan Africa. These include negative social influences,[20-22 26] lack of time, [20 21 23] and fear of injury.[20] In South Africa, issues around personal safety and crime emerged as major barriers to physical activity, particularly for women.[21 22] However, our participants presented no such concerns. This may reflect the fact Malawi currently experiences lower crime rates than most other Sub-Saharan African countries,[49] but also that our participants were older and perhaps felt less at risk than the younger participants in the other studies may have done.[50-52]

Importantly, almost all participants recognized the need to improve physical activity in Malawi. Table 2 summarizes potential strategies to address barriers at different levels of the ecological model[35 36] for consideration in future interventions. The strategies reflect the barriers and opportunities identified by participants and previous evidence on the effectiveness of embedding physical activity within daily routines,[53] highlighting



For peer review only - http://bmjopen.bmj.com/site/about/guidelines.xhtml

**Table 2 Strategies to improve physical activity levels in Malawi mapped against individual, interpersonal, environment/organisational and policy level barriers**

| Ecological model level | Barrier/opportunity | Strategy |
|---|---|---|
| Individual | **Barriers**<br>• Ill health and fear of injury<br>• Age | 1. Information on health benefits of physical activity.[2,54]<br>2. Information on exercising safely, including as people age.[2,53,55] |
| | **Opportunities**<br>• Maintaining health and wellbeing<br>• Losing weight<br>• Maintaining strength | See 1 and 2 |
| | • Willingness to exercise at home<br>• Recognition of importance of enjoyment | 3. Information on easy to do exercises, which can be done alone without support.[53,55]<br>4. Promotion of a range of activities (including offering taster sessions) to allow people to find something they enjoy doing.[2,56] |
| Interpersonal | **Barriers**<br>• Discouragement (including gender-based) from others<br>• Wealth-related societal expectations<br>• Family responsibilities<br>• Time constraints due to competing priorities | 5. Information campaigns to normalize and promote the value of different forms of physical activity, including for both women and men.[2,54]<br>6. Encourage family involvement in physical activity to motivate each other.[54,57]<br>7. Promoting forms of physical activity that can fit into daily routines i.e., walking.[53,55,56]<br>See also 4 |
| | **Opportunities**<br>• Playing with children<br>• Recognition of the value of group-based activities | 8. Promote the benefits (including bonding) associated with exercising with children.[58]<br>9. Encourage group based physical activity.[53,55,56]<br>See also 4, 6 and 8 |
| Environment/ organizational | **Barriers**<br>• Urban gyms dominated by [young] men<br>• Fewer formal physical activity facilities in rural areas | 10. Set aside female only and other inclusive time slots in gyms.[55]<br>11. Invest in recreational infrastructure such as parks and gyms accessible by both men and women in rural, as well as urban, areas.[2,55,59]<br>12. Encourage people (including those in urban areas) to exercise outdoors using footpaths and parks.[59]<br>See also 5 |
| | **Opportunities**<br>• Churches, schools, community organizations | 13. Encourage church, school and community facilities to promote (social) physical activities.[2,55] |
| | • People engage in sedentary activities at workplaces | 14. Workplace initiatives to improve physical activity (e.g., walking breaks; gym membership) with incentives for participation.[2,53,54,56,59,60]<br>15. Encourage people to park a walking distance from workplace.[53] |
| Policy | **Barriers**<br>• Growth in use of private cars | 16. Improve quality and accessibility of public transport systems.[55]<br>17. Improve availability of footpaths and invest in cycling and other active transport infrastructure.[2,54-56,59,61] |



For peer review only - http://bmjopen.bmj.com/site/about/guidelines.xhtml

573 the personal benefits/value of physical activity,[2 54] group based exercising,[57] and
574 promoting workplace based physical activity.[59 60 62]

575 As Malawi continues to develop economically,[63 64] it is likely that (in part due to the
576 societal expectations associated with personal wealth) requirements for occupational,
577 transport and household physical activity will reduce,[65] meaning that recreational
578 physical activity will become increasingly important to help people maintain healthy
579 levels of physical activity.[59] The current study revealed that people in Malawi are
580 already involved in a range of recreational physical activities. However, urban
581 residents described engaging more in structured, facility-dependent exercising
582 (although there was evidence that some groups felt excluded from gyms), whilst
583 physical activity facilities were not mentioned by rural participants. These findings
584 suggest that work needs to be done in ensuring equality of opportunity in relation to
585 physical activity in Malawi. This would include both investment in accessible physical
586 activity facilities in rural areas, but also the promotion of less formal activities, such as
587 dancing, walking and being active with family members,[21 66-69] in urban areas, to
588 ensure that everyone can find a form of physical activity they enjoy.

589 This study had a number of strengths. First, its design (separate focus groups with
590 men and women in urban and rural settings) allowed systematic comparison of the
591 similarities and differences in men and women's experiences of, and attitudes
592 towards, physical activity in urban and rural Malawi. In addition, the team approach
593 to identifying and defining the analytic themes enhanced the robustness (validity) of
594 our results.[70] However, it was not without limitations. Importantly, the participants
595 had been told they were at high risk of developing diabetes following their
596 participation in previous health research. All had been advised to attend their local
597 health clinic or hospital, where they may have received information about the

For peer review only

importance of physical activity for their health. They were also older and some were dealing with other health conditions, so may have been more likely than the general adult population of Malawi to try to include different forms of physical activity in their daily lives. In addition, whilst focus groups were chosen to support the development of rich accounts of physical activity by enabling participants to build on each other's accounts, there is potential that they may also adjust their own responses to conform to what they perceive as the views of other group members, thus limiting the richness of the data collected. However, the fieldworkers who conducted the focus groups did not take any contextual fieldnotes that would have supported a meaningful analysis of the extent to which the group setting influenced members' responses in this way.[71]

## CONCLUSION

This study provides detailed insights into the physical activity views and practices of men and women in urban and rural Malawi, which are important to inform future interventions to increase physical activity. The reported decline in routine physical activity is likely to continue as Malawi, like other countries in the Sub-Saharan Africa region, undergoes social and economic transition, highlighting the need to promote recreational forms of physical activity. The findings suggest that in addition to information campaigns to raise awareness of the benefits of physical activity for all ages, the importance of finding forms of physical activity that people enjoy and the value of (and opportunities for) being physically active with other people, including children and grandchildren, should also be promoted. Finally, public investment in recreational facilities in rural and urban areas that are fully accessible for women, as well as men, and in active travel infrastructure may also be required to ensure that healthy levels of physical activity continue to be part of everyday life in Malawi.



For peer review only - http://bmjopen.bmj.com/site/about/guidelines.xhtml

**Acknowledgments**

The authors would like to thank: Hazel Namadingo for her input while employed by MEIRU in designing the topic guide, conducting the focus groups in Lilongwe and quality assuring all transcripts; Cecelia Nyirenda for conducting the focus groups and undertaking the transcription and translation in Karonga; and Maisha Nyasulu for the transcription and translation in Lilongwe.

**Contributors**

CMG, CB, JMG and AC designed and conducted the study and acquired the data. JB, CMG and CB conceived and designed the analyses. JB and CMG interpreted the data and contributed to drafting the manuscript. All authors contributed to revising the manuscript critically for important intellectual content and all authors approved the version of the manuscript to be published.

**Funding**

This study was funded by the Medical Research Council (MR/R019428/1). The funder had no influence on the analysis plan, results presented or decision to publish.

**Competing interests**

None declared.

**Participant consent for publication**

Not required.

**Ethics approval**

The study was approved by the National Health Sciences Research Committee in Malawi (18/01/1951) and University of Glasgow College of Medical, Veterinary and

For peer review only

645 Life Sciences Ethics Committee (200170173) in the UK. All participants provided written informed consent prior to their participation in the focus groups.

647 **Data availability statement**

648 Data are available on request from the corresponding author.

For peer review only



For peer review only - http://bmjopen.bmj.com/site/about/guidelines.xhtml

## REFERENCES

1. World Health Organization. Noncommunicable Diseases Progress Monitor 2020: World Health Organization, 2020.

2. World Health Organization. Physical Activity Factsheet. 2018. Available: https://www.who.int/en/news-room/fact-sheets/detail/physical-activity. [accessed January 2020].

3. Darling AM, Sunguya B, Ismail A, et al. Gender differences in nutritional status, diet and physical activity among adolescents in eight countries in sub-Saharan Africa. *Trop Med Int Health* 2020;**25**(1):33-43.

4. Delisle H, Ntandou-Bouzitou G, Agueh V, et al. Urbanisation, nutrition transition and cardiometabolic risk: the Benin study. *Br J Nutr* 2012;**107**(10):1534-44.

5. Kirunda BE, Wamani H, Fadnes LT, et al. Objectively Assessed Physical Activity and Associated Factors Among Adults in Peri-Urban and Rural Eastern Uganda: A Population-based Study. *J Phys Act Health* 2016;**13**(11):1243-54.

6. Barr AL, Young EH, Sandhu MS. Objective measurement of physical activity: improving the evidence base to address non-communicable diseases in Africa. *BMJ Glob Health* 2018;**3**(5):e001044.

7. World Health Organization. Noncommunicable diseases country profiles 2011: World Health Organization, 2011.

8. World Health Organization. Noncommunicable diseases country profiles 2018: World Health Organization, 2018.

9. Divala OH, Amberbir A, Ismail Z, et al. The burden of hypertension, diabetes mellitus, and cardiovascular risk factors among adult Malawians in HIV care: consequences for integrated services. *BMC Public Health* 2016;**16**(1):1243-43.



For peer review only - http://bmjopen.bmj.com/site/about/guidelines.xhtml

10. Msyamboza KP, Ngwira B, Dzowela T, et al. The burden of selected chronic non-communicable diseases and their risk factors in Malawi: nationwide STEPS survey. *PLoS One* 2011;**6**(5):e20316.

11. World Health Organization. Insufficient physical activity: prevalence of insufficient physical activity among adults. 2018. Available: http://gamapserver.who.int/gho/interactive_charts/ncd/risk_factors/physical_inactivity/atlas.html?indicator=i1&date=Male. [accessed March 2020].

12. Price AJ, Crampin AC, Amberbir A, et al. Prevalence of obesity, hypertension, and diabetes, and cascade of care in sub-Saharan Africa: a cross-sectional, population-based study in rural and urban Malawi. *Lancet Diabetes Endocrinol* 2018;**6**(3):208-22.

13. Soares ALG, Banda L, Amberbir A, et al. Sex and area differences in the association between adiposity and lipid profile in Malawi. *BMJ Glob Health* 2019;**4**(5):e001542-e42.

14. Bigna JJ, Noubiap JJ. The rising burden of non-communicable diseases in sub-Saharan Africa. *Lancet Glob Health* 2019;**7**(10):e1295-e96.

15. Gowshall M, Taylor-Robinson SD. The increasing prevalence of non-communicable diseases in low-middle income countries: the view from Malawi. *Int J Gen Med* 2018;**11**:255-64.

16. World Health Organization. Projections of mortality and causes of death, 2016 to 2060. 2018. Available: https://www.who.int/healthinfo/global_burden_disease/projections/en/. [accessed January 2020].

17. Anderson E, Durstine JL. Physical activity, exercise, and chronic diseases: A brief review. *Sports Med Health Sci* 2019;**1**(1):3-10.



For peer review only - http://bmjopen.bmj.com/site/about/guidelines.xhtml

18. Warburton DER, Nicol CW, Bredin SSD. Health benefits of physical activity: the evidence. *Can Med Assoc J* 2006;**174**(6):801.

19. Milton K, Macniven R, Bauman A. Review of the epidemiological evidence for physical activity and health from low- and middle-income countries. *Glob Public Health* 2014;**9**(4):369-81.

20. Tuakli-Wosornu YA, Rowan M, Gittelsohn J. Perceptions of physical activity, activity preferences and health among a group of adult women in urban Ghana: a pilot study. *Ghana Med J* 2014;**48**(1):3-13.

21. Watson ED, Norris SA, Draper CE, et al. "Just because you're pregnant, doesn't mean you're sick!" A qualitative study of beliefs regarding physical activity in black South African women. *BMC Pregnancy Childbirth* 2016;**16**(1):174-74.

22. Ware LJ, Prioreschi A, Bosire E, et al. Environmental, Social, and Structural Constraints for Health Behavior: Perceptions of Young Urban Black Women During the Preconception Period-A Healthy Life Trajectories Initiative. *J Nutr Educ Behav* 2019;**51**(8):946-57.

23. Phillips EA, Comeau DL, Pisa PT, et al. Perceptions of diet, physical activity, and obesity-related health among black daughter-mother pairs in Soweto, South Africa: a qualitative study. *BMC Public Health* 2016;**16**(1):750.

24. Mayega RW, Etajak S, Rutebemberwa E, et al. 'Change means sacrificing a good life': perceptions about severity of type 2 diabetes and preventive lifestyles among people afflicted or at high risk of type 2 diabetes in Iganga Uganda. *BMC Public Health* 2014;**14**(1):864.

25. Ogunrinu T, Gamboa-Maldonado T, Ngewa RN, et al. A qualitative study of health education experiences and self-management practices among patients with type 2



For peer review only - http://bmjopen.bmj.com/site/about/guidelines.xhtml

diabetes at Malamulo Adventist Hospital in Thyolo District, Malawi. *Malawi Med J* 2017;**29**(2):118-23.

26. Mphwanthe G, Carolan M, Earnesty D, et al. Perceived barriers and facilitators to diet and physical activity among adults diagnosed with type 2 diabetes in Malawi. *Glob Public Health* 2021;**16**(3):469-84.

27. Malambo P, Kengne AP, Lambert EV, et al. Prevalence and socio-demographic correlates of physical activity levels among South African adults in Cape Town and Mount Frere communities in 2008-2009. *Arch Public Health* 2016;**74**:54-54.

28. Guthold R, Louazani SA, Riley LM, et al. Physical activity in 22 African countries: results from the World Health Organization STEPwise approach to chronic disease risk factor surveillance. *Am J Prev Med* 2011;**41**(1):52-60.

29. Hoare E, Stavreski B, Jennings GL, et al. Exploring Motivation and Barriers to Physical Activity among Active and Inactive Australian Adults. *Sports (Basel)* 2017;**5**(3):47.

30. Chaabane S, Chaabna K, Abraham A, et al. Physical activity and sedentary behaviour in the Middle East and North Africa: An overview of systematic reviews and meta-analysis. *Sci Rep* 2020;**10**(1):9363.

31. Leung FH, Savithiri R. Spotlight on focus groups. *Canadian family physician Medecin de famille canadien* 2009;**55**(2):218-9.

32. Morgan DL. Paradigms Lost and Pragmatism Regained: Methodological Implications of Combining Qualitative and Quantitative Methods. *Journal of Mixed Methods Research* 2007;**1**(1):48-76.

33. Braun V, Clarke V, Hayfield N, et al. Thematic Analysis. In: Liamputtong P, ed. Handbook of Research Methods in Health Social Sciences. Singapore: Springer Singapore, 2019:843-60.



For peer review only - http://bmjopen.bmj.com/site/about/guidelines.xhtml

34. Ziebland S, McPherson A. Making sense of qualitative data analysis: an introduction with illustrations from DIPEx (personal experiences of health and illness). *Medical education* 2006;**40**(5):405-14.

35. Bauman AE, Reis RS, Sallis JF, et al. Correlates of physical activity: why are some people physically active and others not? *Lancet* 2012;**380**(9838):258-71.

36. Sallis JF, Owen N, Fisher EB. Ecological Models of Health Behaviours. In: Glanz K, Rimer BK, Viswanath K, eds. Health Behaviour and Health Education: Theory, Research and Practice. 4th ed. San Francisco: Jossey-Bass, 2008:465-85.

37. Luke R. Car ownership perceptions and intentions amongst South African students. *J Transp Geogr* 2018;**66**:135-43.

38. National Statistical Office, ICF. Malawi Demographic and Health Survey 2015-16. Zomba, Malawi, and Rockville, Maryland, USA: NSO and ICF, 2017.

39. World Health Organization. Global Recommendations on Physical Activity for Health. Geneva: World Health Organization, 2010.

40. World Health Organization. WHO guidelines on physical activity and sedentary behaviour. Geneva: World Health Organisation, 2020.

41. Department of Health and Social Care. UK Chief Medical Officers' Physical Activity Guidelines. UK: Department of Health and Social Care, 2019:66.

42. Hillsdon M, Foster C. What are the health benefits of muscle and bone strengthening and balance activities across life stages and specific health outcomes? *J Frailty Sarcopenia Falls* 2018;**3**(2):66-73.

43. Aboderin IAG, Beard JR. Older people's health in sub-Saharan Africa. *Lancet* 2015;**385**(9968):e9-e11.

44. Flax VL, Thakwalakwa C, Phuka JC, et al. Body size preferences and food choice among mothers and children in Malawi. *Matern Child Nutr* 2020;**16**(4):e13024.

For peer review only

45. Okoro EO, Oyejola BA, Etebu EN, et al. Body size preference among Yoruba in three Nigerian communities. *Eat Weight Disord* 2014;**19**(1):77-88.

46. Matoti-Mvalo T, Puoane T. Perceptions of body size and its association with HIV/AIDS. *South Afr J Clin Nutr* 2011;**24**(1):40-45.

47. Puoane T, Tsolekile L, Steyn N. Perceptions about body image and sizes among Black African girls living in Cape Town. *Ethn Dis* 2010;**20**(1):29-34.

48. Benkeser RM, Biritwum R, Hill AG. Prevalence of overweight and obesity and perception of healthy and desirable body size in urban, Ghanaian women. *Ghana Med J* 2012;**46**(2):66-75.

49. Institute for Economics and Peace. Global Peace Index 2020: Measuring Peace in a Complex World. Sydney, 2020.

50. Kayira J. Malawi: Rape, defilement cases horrify a nation. 2020. Available: https://face2faceafrica.com/article/malawi-rape-defilement-cases-horrify-a-nation. [accessed August 2021].

51. Masiye M. Defilement, Rape Up to 43% in Eastern Region. 2020. Available: https://zodiakmalawi.com/nw/national-news/66-news-in-southern-region/2893-defilement-rape-up-to-43-in-eastern-region. [accessed August 2021].

52. Sapuli D. Malawi has over 2100 child rapists in its prisons. 2020. Available: https://malawi24.com/2020/10/30/malawi-has-over-2100-child-rapists-in-its-prisons/. [accessed August 2021].

53. Centers for Disease Control and Prevention. Overcoming Barriers to Physical Activity. 2020. Available: https://www.cdc.gov/physicalactivity/basics/adding-pa/barriers.html. [accessed September 2020].

54. Tuso P. Strategies to Increase Physical Activity. *Perm J* 2015;**19**(4):84-88.




For peer review only - http://bmjopen.bmj.com/site/about/guidelines.xhtml

55. Heath GW, Parra DC, Sarmiento OL, et al. Evidence-based intervention in physical activity: lessons from around the world. *Lancet* 2012;**380**(9838):272-81.

56. Ferguson-Stegall L, Robb JD. Effective Strategies to Increase Physical Activity in the Working Years. *ACSMs Health Fit J* 2019;**23**(5).

57. Brandt CJ, Clemensen J, Nielsen JB, et al. Drivers for successful long-term lifestyle change, the role of e-health: a qualitative interview study. *BMJ Open* 2018;**8**(3):e017466.

58. Paquette D, Dumont C. Is father–child rough-and-tumble play associated with attachment or activation relationships? *Early Child Development and Care* 2013;**183**(6):760-73.

59. Guthold R, Stevens GA, Riley LM, et al. Worldwide trends in insufficient physical activity from 2001 to 2016: a pooled analysis of 358 population-based surveys with 1.9 million participants. *Lancet Glob Health* 2018;**6**(10):e1077-e86.

60. Centers for Disease Control and Prevention. Worksite Physical Activity. September 28, 2017 2017. Available: https://www.cdc.gov/physicalactivity/worksite-pa/index.htm. [accessed September 2020].

61. Brown DR, Heath GW, Martin SL, editors. *Promoting Physical Activity: A Guide for Community Action*. 2 ed. Champaign, IL: Human Kinetics, 2010.

62. World Health Organization. Global action plan on physical activity 2018–2030: more active people for a healthier world: World Health Organization, 2018.

63. National Planning Commission. Malawi's vision: An inclusively wealthy and self-reliant nation. Lilongwe, Malawi: National Planning Commission, 2020.

For peer review only



For peer review only - http://bmjopen.bmj.com/site/about/guidelines.xhtml

64. Government of Malawi. Malawi Growth and Development Strategy III: Building a Productive, Competitive and Resilient Nation. Lilongwe, Malawi: Government of Malawi, 2017.

65. Ng SW, Popkin BM. Time use and physical activity: a shift away from movement across the globe. *Obes Rev* 2012;**13**(8):659-80.

66. Chaabane S, Chaabna K, Doraiswamy S, et al. Barriers and Facilitators Associated with Physical Activity in the Middle East and North Africa Region: A Systematic Overview. *J Environ Res Public Health* 2021;**18**(4).

67. Harrison AL, Taylor NF, Shields N, et al. Attitudes, barriers and enablers to physical activity in pregnant women: a systematic review. *J Physiother* 2018;**64**(1):24-32.

68. Saligheh M, McNamara B, Rooney R. Perceived barriers and enablers of physical activity in postpartum women: a qualitative approach. *BMC Pregnancy Childbirth* 2016;**16**(1):131.

69. Beune EJAJ, Haafkens JA, Agyemang C, et al. Inhibitors and enablers of physical activity in multiethnic hypertensive patients: qualitative study. *J Hum Hypertens* 2010;**24**(4):280-90.

70. Morse JM. Critical Analysis of Strategies for Determining Rigor in Qualitative Inquiry. *Qualitative health research* 2015;**25**(9):1212-22.

71. Carey MA, Smith MW. Capturing the Group Effect in Focus Groups: A Special Concern in Analysis. *Qualitative health research* 1994;**4**(1):123-27.

For peer review only



For peer review only - http://bmjopen.bmj.com/site/about/guidelines.xhtml

## Supplementary materials

## Physical activity focus group topic guide

1. Tell me about when you are physically active/do exercise in your daily life. What sort of things do you do? Where? Why? (Prompt for work, travel and leisure activity)
2. How would you say your activity levels compare with your parents/grandparents? Why is this? (Prompt changes to work, leisure and travel – moving to different place)
3. Have your own activity levels changed over the years? Why is this?
4. What are the benefits to you of being physically active? (Prompt: fitness, strength, flexibility) how important are these to you/other people in your community? Why is this?
5. What do you understand by exercise?
6. Do you ever do exercise? What do you do and where? Who do you do it with? What motivates you to do exercise?
7. Tell me about any social/community occasions which involve physical activity/exercise?
8. What do you know about physical activity and health? ([Prompt: Benefits and risks of being physically active, including in relation to diabetes) How do you know this? (Prompt: where did this knowledge come from?)
9. How could you do more physical activity in your daily life? What might stop you doing this?

Demonstrate and lead an exercise session including resistance exercises. Ask the participants what they think of them as they do them.

10. Afterwards, ask: which of these exercises might you do again (if any)? Why have you chosen these? What would help you do these regularly? What would stop you doing them regularly?
11. Which of the exercises would you not like to do again? Why is this?

For peer review only

**Table S1: Detailed description and example extracts from the nine broad themes reported**

| Theme | Description | Example extracts |
|---|---|---|
| *Context* | | |
| 1. Types of physical activity undertaken | Activities participants reported doing that made them physically active or inactive | *The physical work which I usually do is, I like going to the lake and launch the fish nets. When I come back home, I make sure that outside the house is clean, but if it's not clean I tell the children to sweep the ground. Sometimes when we are in rainy season, I wake up early in the morning going to the farm garden, I do weeding – a little bit in maize garden because I don't do for a long time. Usually my work is fishing, I got in the morning going to the lake and arrange the fishing nets and go into the lake.* (Male, 76, rural, hypertension reported)<br><br>*In the morning I take my bath then I prepare to go to work, and mostly I do my work while seated working on the computer, if I move around that means I'm just delivering file to my boss's office, when I knock off from work I use a car off I go home.* (Male, 50, urban, no health conditions reported) |
| 2. Changes in physical activity over time | How physical activity has changed from generation to generation. | *in the past people were digging manually by using a hoe while nowadays we are using animal plough whenever we want to cultivate any kind of crop. The other thing is, they* [past generations] *were carrying things on their head while nowadays we carry everything in an oxcart or in a vehicle … they like building structures while nowadays we like modern structures by hiring other people to do for us while we are sitting down. …* (Male, 25, rural, no health conditions reported) |
| *Influences* | | |
| 3. Physical activity and health | How participants perceive that being physically active influences people's health | *If I stay idle, diabetes disease will affect me because there is nothing I am doing. Any kind of disease can affect you if you are just staying idle but if I am physically active, I might be free from diseases and be strong*. (Female, 60, rural, hypertension reported) |
| 4. Motivations | Participants' reasons for undertaking different physical activities | *The reason we are physically active especially here on earth, it's because we want to eat. Without this, there won't be anything to eat*. (Female, 56, rural, diabetes reported) |
| 5. Barriers to physical activity | Factors hindering involvement in physical activity | *It's what we said that sometimes its laziness because you can plan to do the exercise, but you may end up doing other things*. (Female, 60, rural, hypertension reported)<br><br>*[the] gym is too far from where I live, I live [in one place] and gym is in [another place], mostly I fail to go to gym, and when I* |

For peer review only

| Theme | Description | Example extracts |
|---|---|---|
| | | *go to gym am supposed to pay a certain fee which makes me just give up on gym, but if I had all the needed weights at home I could have been doing exercises at home*. (Male, 38, urban, no health condition reported) |
| 6. Technology and physical activity | How technological advancements affect physical activity | *Technology is pushing us to be…. the same with those who live in rural areas…. let's talk of phones, even maize mills are everywhere nowadays, they no longer use mortar and pestle*. (Female, 34, urban, no health condition reported) |
| 7. Social norms | How other people's views/commonly held opinions and expectations influence physical activity | *… My neighbor's asked me why I draw water by myself when I have children, I tell them it's part of my exercises*. (Female, 54, urban, no health conditions reported)<br>*There are some other exercises that women cannot manage to do like stretching your legs you cannot manage that…* (Female, 58, urban, hypertension reported) |
| 8. Social support | People and social institutions providing support for physical activities | *At our church we have our own team as [name of church] women we play against the youths for practices, but when we have a match, we play against another [name of church] church*. (Female, 59, urban, hypertension reported) |
| *Solutions* | | |
| 9. Ways of improving physical activity | Suggested ways of improving physical activity | *I think a person should join more sports groups, working can be one of them and cycling can be one of them; you can also join groups which can motivate you to be moving or running and make you sweat.* (Male, 25, rural, no health conditions reported) |

For peer review only

**Table S2 Types of physical activity described by participants within the household, occupational, transport and recreational domains of the ecological model**

| Household* | Who | | | | Occupational* | Who | | | | Transport | Who | | | | Recreational | Who | | | |
|---|---|---|---|---|---|---|---|---|---|---|---|---|---|---|---|---|---|---|---|
| | wr | wu | mr | mu | | wr | wu | mr | mu | | wr | wu | mr | mu | | wr | wu | mr | mu |
| Chores (sweeping, mopping) | x | x | - | x | Farming (planting, weeding, harvesting) | x | - | x | - | Walking | x | x | x | x | Dance | x | x | x | - |
| Food shopping | x | x | x | - | Looking after livestock | - | - | x | - | Cycling | - | - | - | x | Team sports | x | x | x | x |
| Collecting farm produce for meals | x | - | - | - | Fishing on lake | - | - | x | - | Public transport | x | - | x | x | Playing with children | x | x | x | x |
| Cooking | x | x | - | x | Running shops/ market stalls | - | x | - | x | (Bicycle) taxi | - | - | x | x | Jogging | - | x | x | x |
| Food processing (pounding maize) | x | - | - | - | Service industry | - | - | - | x | Personal car | - | x | x | x | Cycling | - | - | x | x |
| Washing clothes | x | x | - | - | Office job | - | - | - | x | Transport goods (by car/ox cart) | - | x | x | - | Gym/exercise classes | - | x | - | x |
| Fetching water | x | x | - | - | | | | | | | | | | | Doing exercises at home | x | x | - | x |
| Cutting wood | - | - | x | - | | | | | | | | | | | | | | | |
| Carrying wood | x | - | - | - | | | | | | | | | | | | | | | |
| Gardening | - | x | x | x | | | | | | | | | | | | | | | |
| Moulding bricks | - | - | x | - | | | | | | | | | | | | | | | |
| Digging pit latrines | - | - | x | - | | | | | | | | | | | | | | | |
| Car washing, maintenance | - | - | - | x | | | | | | | | | | | | | | | |
| Feeding livestock | - | x | - | - | | | | | | | | | | | | | | | |

*In the rural setting there appeared to be some overlap between household and occupational physical activities. wr = rural women, wu = urban women, mr = rural men, mu = urban men

For peer review only

**Table S3: Broad themes and sub-themes summarising influences and their impact on participants' physical activity, mapped to ecological model levels and domains**

| Theme | Sub-theme | Who | | | | Ecological Model | | Impact (+ve/-ve) |
|---|---|---|---|---|---|---|---|---|
| | | wr | wu | mr | mu | Level | Domain | |
| 3) Health | Recognition of health/wellbeing benefits | x | x | x | x | Individual | General | +ve |
| 3) Health | Weight loss | x | x | x | x | Individual | General | +ve |
| 3) Health | Recognition of physical strength benefits | x | x | x | x | Individual | General | +ve |
| 4) Motivations | Family responsibilities | x | - | - | - | Interpersonal | Household | +ve |
| 4) Motivations | Work responsibilities | x | - | x | - | Interpersonal | Occupational | +ve |
| 5) Barriers | Work responsibilities | - | x | - | x | Interpersonal | Recreational | -ve |
| 5) Barriers | Family responsibilities | x | x | x | x | Interpersonal | Recreational | -ve |
| 5) Barriers | Lack of time | - | x | - | x | Individual | Recreational | -ve |
| 5) Barriers | Sense of urgency | - | x | - | x | Individual | General | -ve |
| 5) Barriers | Age | x | x | x | x | Individual | General | -ve |
| 5) Barriers | Ill health | x | x | x | x | Individual | General | -ve |
| 5) Barriers | Fear of injury | x | x | x | x | Individual | General | -ve |
| 5) Barriers | Poor diet | x | x | x | x | Individual | General | -ve |
| 5) Barriers | Lack of exercise knowledge | x | - | - | - | Individual | Recreational | -ve |
| 5) Barriers | Laziness | x | x | x | x | Individual | General | -ve |
| 6) Technology | Maize mills | x | x | - | - | Environment | Household | -ve |
| 6) Technology | Farming tools/machinery | - | - | x | - | Environment | Occupational | -ve |
| 7) Social norms | Pressure to make money | - | - | - | x | Interpersonal | Occupational | -ve |
| 7) Social norms | Wealth | x | x | x | x | Interpersonal | Household/Transport | -ve |
| 7) Social norms | Availability of motorised transport | x | x | x | x | Environment | Transport | -ve |
| 7) Social norms | People making fun of you (e.g., cycling) | - | - | - | x | Interpersonal | Transport | -ve |
| 7) Social norms | Gender (e.g., at gym) | - | x | - | - | Interpersonal | Recreational | -ve |
| 7) Social norms | Expectations that children will help | x | x | x | - | Interpersonal | Household | -ve |
| 7) Social norms | Children making fun of you | x | x | - | - | Interpersonal | Recreational | -ve |
| 8) Social support | Playing with children | x | x | x | x | Interpersonal | Recreational | +ve |
| 8) Social support | Team sports/exercising with others | x | x | x | x | Interpersonal | Recreational | +ve |

wr = rural women, wu = urban women, mr = rural men, mu = urban men

For peer review only

# Standards for Reporting Qualitative Research (SRQR)*

http://www.equator-network.org/reporting-guidelines/srqr/

## Title and abstract

| | Page/line no(s). |
|---|---|
| **Title** - Concise description of the nature and topic of the study Identifying the study as qualitative or indicating the approach (e.g., ethnography, grounded theory) or data collection methods (e.g., interview, focus group) is recommended | Page 1, lines 1-2 |
| **Abstract** - Summary of key elements of the study using the abstract format of the intended publication; typically includes background, purpose, methods, results, and conclusions | Page 2-3, lines 20-47 |

## Introduction

| | |
|---|---|
| **Problem formulation** - Description and significance of the problem/phenomenon studied; review of relevant theory and empirical work; problem statement | Pages 4-6, lines 64-115 |
| **Purpose or research question** - Purpose of the study and specific objectives or questions | Page 6, lines 116-124 |

## Methods

| | |
|---|---|
| **Qualitative approach and research paradigm** - Qualitative approach (e.g., ethnography, grounded theory, case study, phenomenology, narrative research) and guiding theory if appropriate; identifying the research paradigm (e.g., postpositivist, constructivist/ interpretivist) is also recommended; rationale** | Page 6 lines 127-135<br>Page 8, lines 161-162 |
| **Researcher characteristics and reflexivity** - Researchers' characteristics that may influence the research, including personal attributes, qualifications/experience, relationship with participants, assumptions, and/or presuppositions; potential or actual interaction between researchers' characteristics and the research questions, approach, methods, results, and/or transferability | Page 8, lines 167-178 |
| **Context** - Setting/site and salient contextual factors; rationale** | Page 7, lines 137-143 |
| **Sampling strategy** - How and why research participants, documents, or events were selected; criteria for deciding when no further sampling was necessary (e.g., sampling saturation); rationale** | Page 7, lines 138-146 |
| **Ethical issues pertaining to human subjects** - Documentation of approval by an appropriate ethics review board and participant consent, or explanation for lack thereof; other confidentiality and data security issues | Page 7, lines 143-146;<br>Page 8, lines 160-161;<br>Page 9, lines 202-205;<br>Page 29, lines 641-644 |
| **Data collection methods** - Types of data collected; details of data collection procedures including (as appropriate) start and stop dates of data collection and analysis, iterative process, triangulation of sources/methods, and modification of procedures in response to evolving study findings; rationale** | Pages 6, lines 127-131;<br>Page 7, line 137;<br>Page 8, lines 160-173 |
| **Data collection instruments and technologies** - Description of instruments (e.g., interview guides, questionnaires) and devices (e.g., audio recorders) used for data collection; if/how the instrument(s) changed over the course of the study | Supplementary material<br>Page 8, lines 160-170 |



For peer review only - http://bmjopen.bmj.com/site/about/guidelines.xhtml

| | |
|---|---|
| **Units of study** - Number and relevant characteristics of participants, documents, or events included in the study; level of participation (could be reported in results) | Page 7, lines 138-152;<br>Pages 9-11, lines 207-221;<br>Table 1 |
| **Data processing** - Methods for processing data prior to and during analysis, including transcription, data entry, data management and security, verification of data integrity, data coding, and anonymization/de-identification of excerpts | Page 8, lines 174-178;<br>Page 9, lines 202-205 |
| **Data analysis** - Process by which inferences, themes, etc., were identified and developed, including the researchers involved in data analysis; usually references a specific paradigm or approach; rationale** | Pages 8-9, lines 180-202 |
| **Techniques to enhance trustworthiness** - Techniques to enhance trustworthiness and credibility of data analysis (e.g., member checking, audit trail, triangulation); rationale** | Pages 8-9, lines 180-202 |

**Results/findings**

| | |
|---|---|
| **Synthesis and interpretation** - Main findings (e.g., interpretations, inferences, and themes); might include development of a theory or model, or integration with prior research or theory | Pages 11-23, lines 224-503;<br>Supplementary materials Tables S2 and S3 |
| **Links to empirical data** - Evidence (e.g., quotes, field notes, text excerpts, photographs) to substantiate analytic findings | Pages 12-23, lines 238-503;<br>Supplementary materials, Table S1 |

**Discussion**

| | |
|---|---|
| **Integration with prior work, implications, transferability, and contribution(s) to the field -** Short summary of main findings; explanation of how findings and conclusions connect to, support, elaborate on, or challenge conclusions of earlier scholarship; discussion of scope of application/generalizability; identification of unique contribution(s) to scholarship in a discipline or field | Pages 23-27, lines 504-588 |
| **Limitations** - Trustworthiness and limitations of findings | Page 3, lines 50-60;<br>Pages 27-28, lines 589-607 |

**Other**

| | |
|---|---|
| **Conflicts of interest** - Potential sources of influence or perceived influence on study conduct and conclusions; how these were managed | Page 29, line 638 |
| **Funding** - Sources of funding and other support; role of funders in data collection, interpretation, and reporting | Page 29, lines 634-636 |

*The authors created the SRQR by searching the literature to identify guidelines, reporting standards, and critical appraisal criteria for qualitative research; reviewing the reference lists of retrieved sources; and contacting experts to gain feedback. The SRQR aims to improve the transparency of all aspects of qualitative research by providing clear standards for reporting qualitative research.
**The rationale should briefly discuss the justification for choosing that theory, approach, method, or technique rather than other options available, the assumptions and limitations implicit in those choices, and how those choices influence study conclusions and transferability. As appropriate, the rationale for several items might be discussed together.

For peer review only



For peer review only - http://bmjopen.bmj.com/site/about/guidelines.xhtml

**Reference:**

O'Brien BC, Harris IB, Beckman TJ, Reed DA, Cook DA. **Standards for reporting qualitative research: a synthesis of recommendations.** *Academic Medicine*, Vol. 89, No. 9 / Sept 2014
DOI: 10.1097/ACM.0000000000000388

For peer review only



For peer review only - http://bmjopen.bmj.com/site/about/guidelines.xhtml
